# Supplementary material for: Novel Oxadiazole-Quinoxalines as Hybrid Scaffolds with Antitumor Activity
Source: Int J Mol Sci. 2025 Feb 8;26(4):1439. doi: 10.3390/ijms26041439 (PMC11855764; doi:10.3390/ijms26041439)
Supplement: Supplementary file 1 [file ijms-26-01439-s001.zip › ijms-3446425-supplementary.pdf]

Supplementary Material

Novel oxadiazole-quinoxalines as hybrid scaffolds with antitumor activity

Paola Corona[1,*], Stefania Gessi[2], Roberta Ibba[1], Stefania Merighi[2], Prisco Mirandola[3], Gérard A. Pinna[1], Manuela Nigro[2], Giulia Pozzi[3], Battistina Asproni[1], Alessia Travagli[2], Sandra Piras[1], Antonio Carta[1], Paola Caria[4] and Gabriele Murineddu[1]

[1]*Department of Medicine, Surgery and Pharmacy, University of Sassari, 07100 Sassari, Italy; ribba@uniss.it (R.I); pinger@uniss.it (G.A.P.); asproni@uniss.it (B.A.); piras@uniss.it (S.P.); acarta@uniss.it (A.C.); muri@uniss.it (G.M.)*

[2]*Department of Translational Medicine, University of Ferrara, 44121 Ferrara, Italy; gss@unife.it (S.G.); mhs@unife.it (S.M.); ngrmnl@unife.it (M.N.); trvlss@unife.it (A.T.)*

[3]*Department of Medicine and Surgery, University of Parma, Via Gramsci 14, 43126 Parma, Italy; prisco.mirandola@unipr.it (P.M.); giulia.pozzi@unipr.it (G.P.)*

[4]*Department of Biomedical Sciences, University of Cagliari, Cittadella Universitaria, 09042 Monserrato, Cagliari, Italy; paola.caria@unica.it (P.C.)*

* Correspondence: *pcorona@uniss.it (P.C.)*

Contents

Figure S1 – S6. NOESY spectra of isomers **34 – 39**

Figure S7 – S31. ^{1}H- and ^{13}C-NMR spectra of compounds **4 – 28**

Figure S32 – S56. One dose assays of compounds **4 – 28**

Figure S57 – S59. 5-Dose assays of compounds **24 – 26**

Figure S60 – S62. Mean graphs of compounds **24 – 26**

Figure S63 – S65. Dose response curves of compound **24 – 26**

Table S1. Percentage of cytotoxicity of compounds **4, 5, 6, 8, 9, 10, 11, 13, 14, 23, 24, 25** and **26** (10 μM) on NB4 cells viability.

Table S2. Percentage of cytotoxicity for compounds **4, 8, 9, 24** and **26** on NB4 cells viability at 100 nM and 1 μM concentrations.

Table S3. Percentage of cytotoxicity of compounds **4, 5, 6, 8, 9, 10, 11, 13, 14, 23, 24, 25** and **26** (10 μM) on JURKAT cells viability.

Table S4. Percentage of cytotoxicity of compounds **4, 5, 6, 8, 9, 10, 11, 13, 14, 23, 24, 25** and **26** (10 μM) on SH-SY5Y cells viability.

Table S5. Percentage of cytotoxicity of compounds **4, 5, 6, 8, 9, 10, 11, 13, 14, 23, 24, 25** and **26** (10 μM) on A375 cells viability.

Table S6. Percentage of cytotoxicity of compounds **4, 5, 6, 8, 9, 10, 11, 13, 14, 23, 24, 25** and **26** (10 μM) on MAHLAVU cells viability.

Table S7. Percentage of cytotoxicity of compounds **11, 24, 25** and **26** (10 μM) on lymphocytes cells viability.

Figure S1. NOESY spectrum of isomer **34**

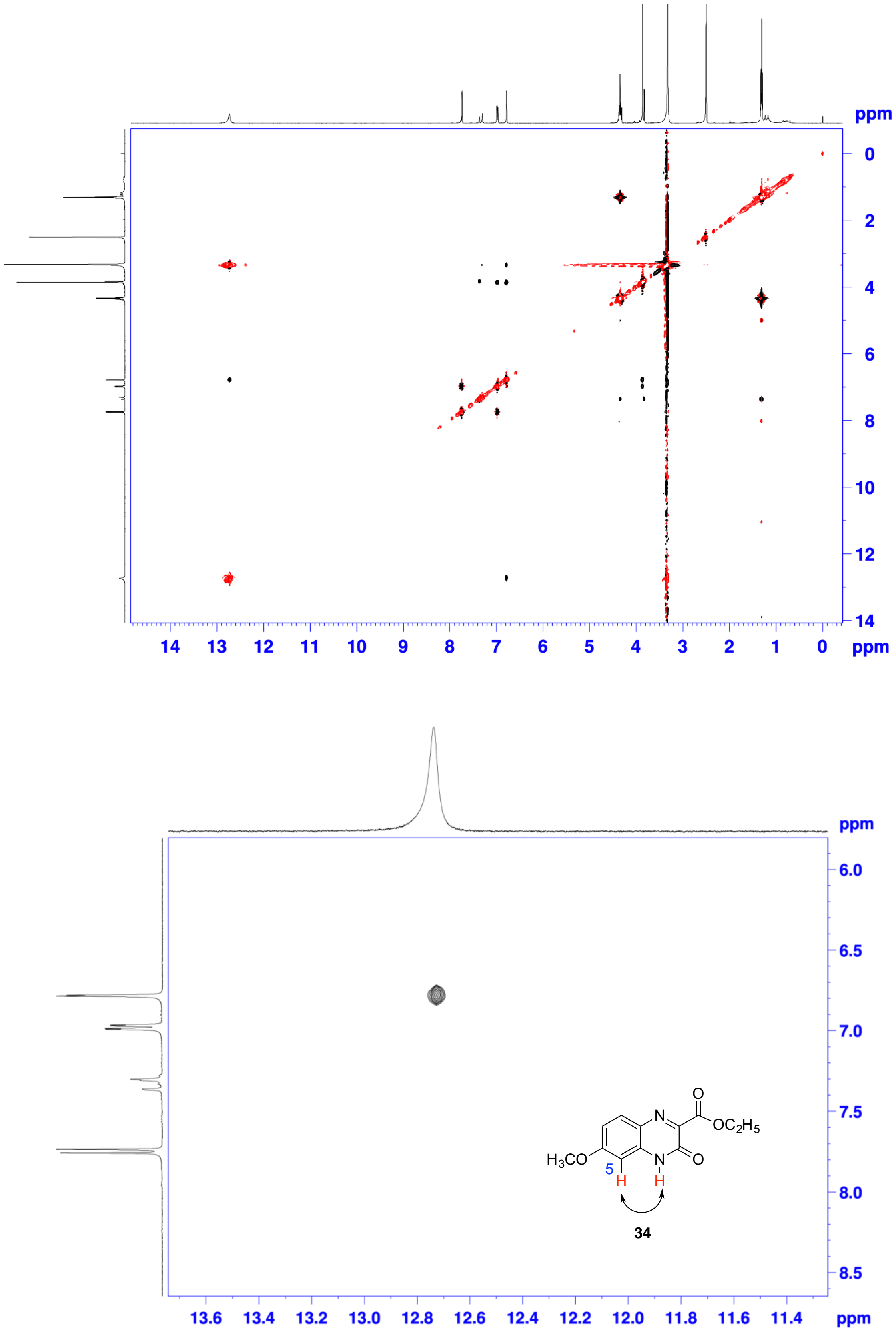

Figure S2. NOESY spectrum of isomer **35**

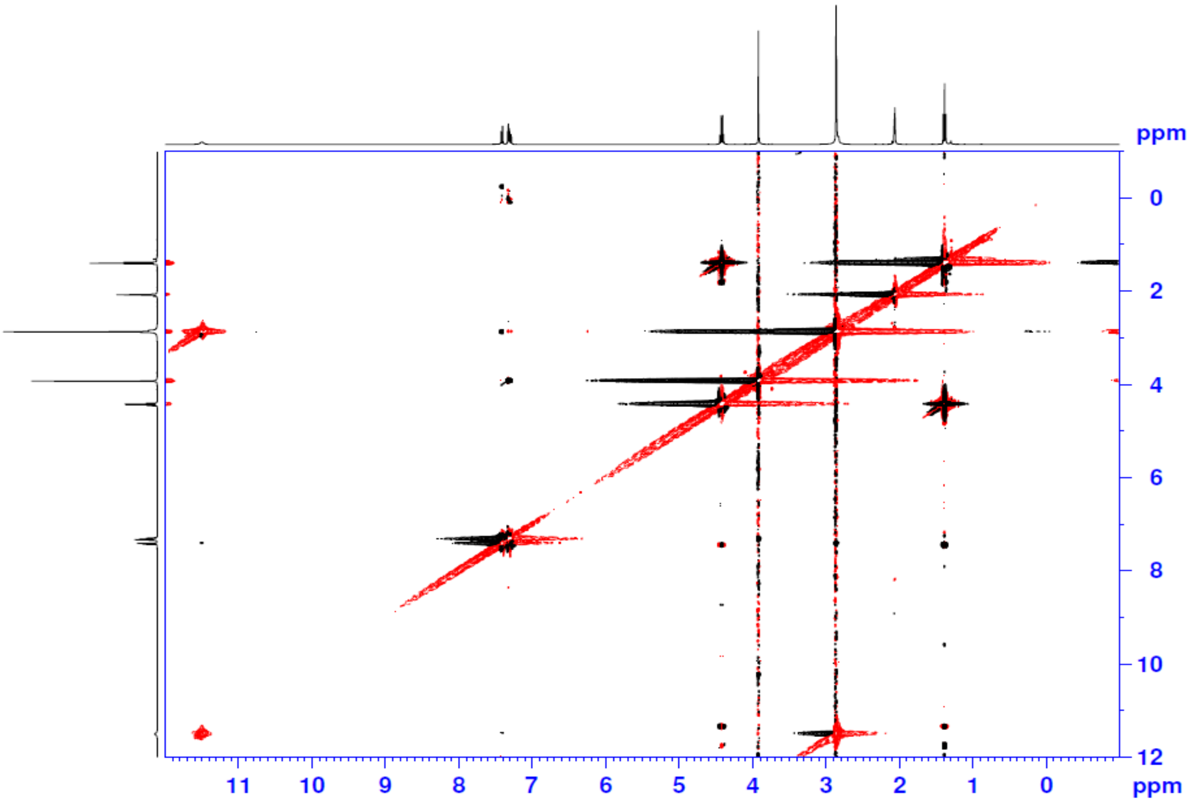

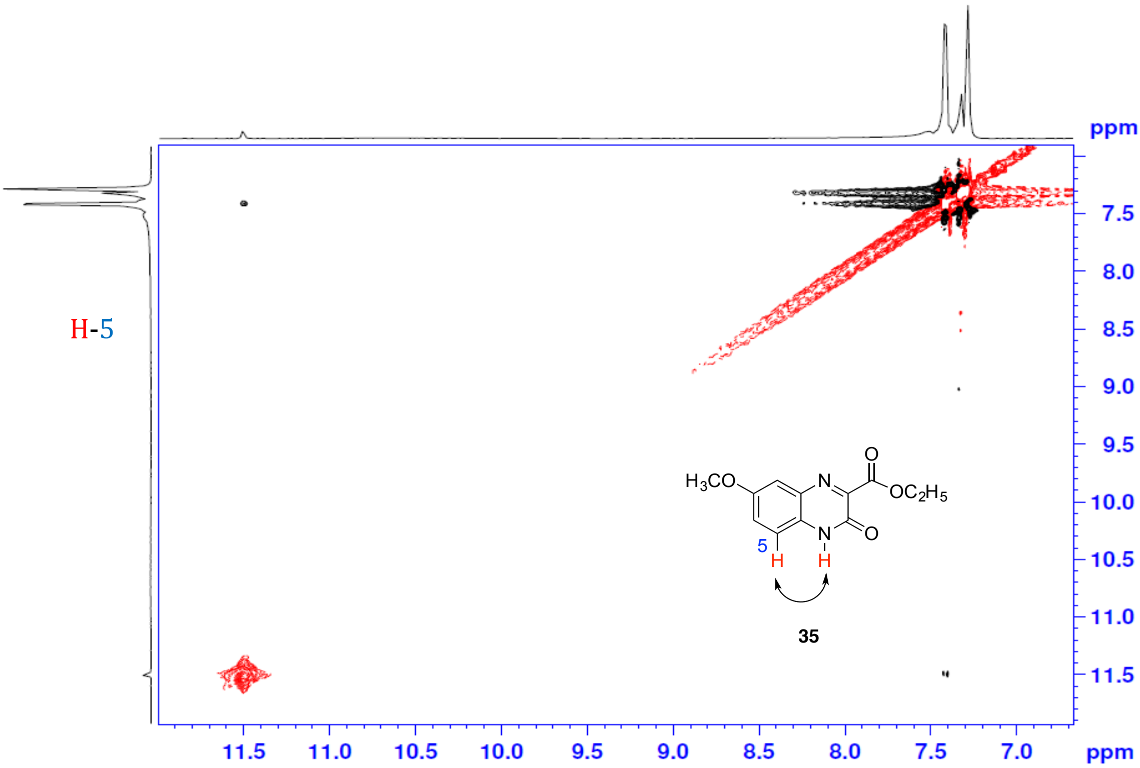

Figure S3. NOESY spectrum of isomer **36**

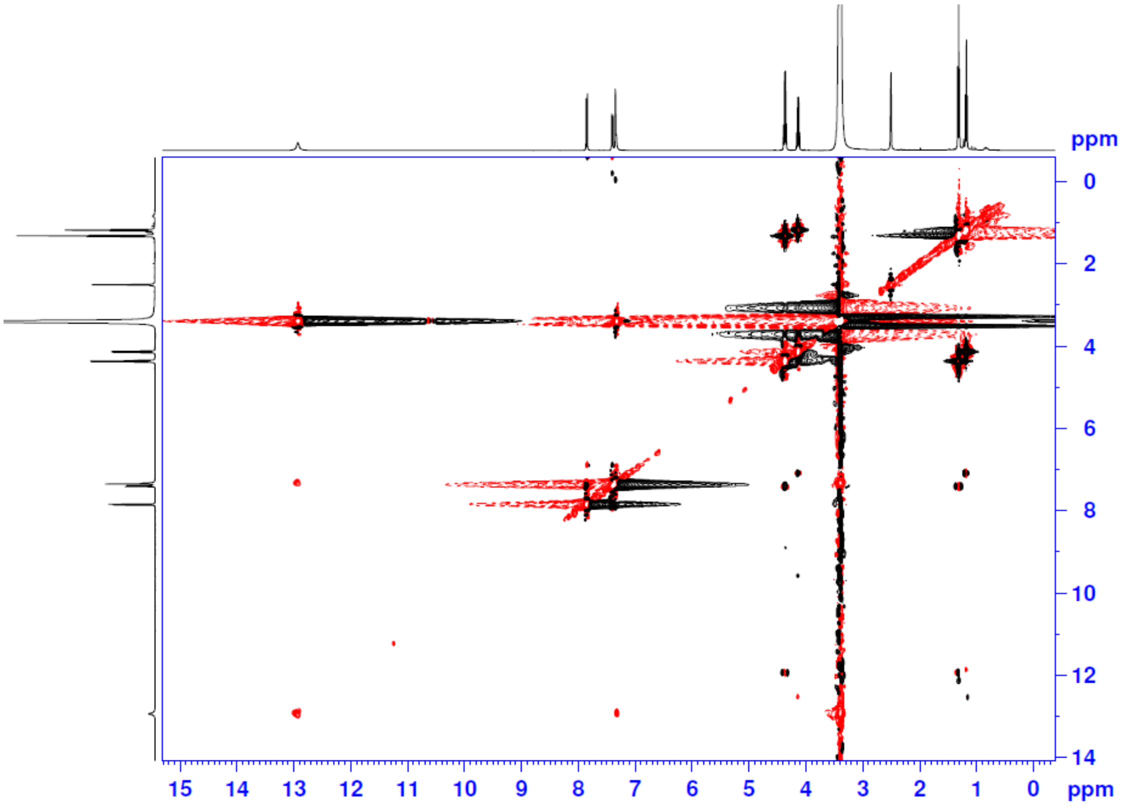

1NM x noesy sw=15

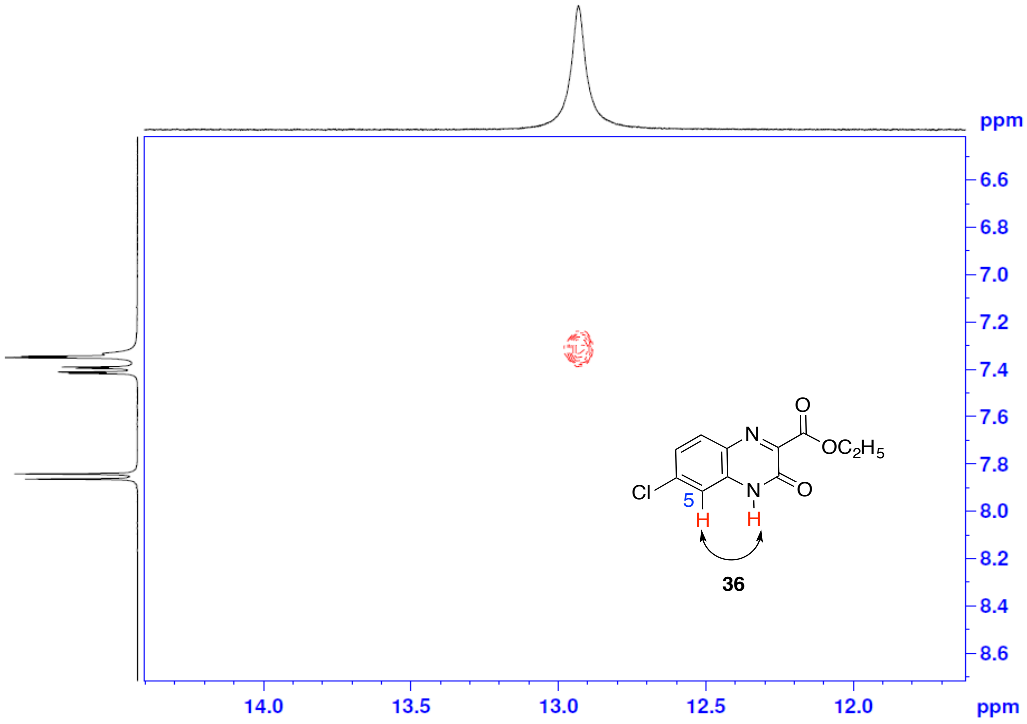

Figure S4. NOESY spectrum of isomer **37**

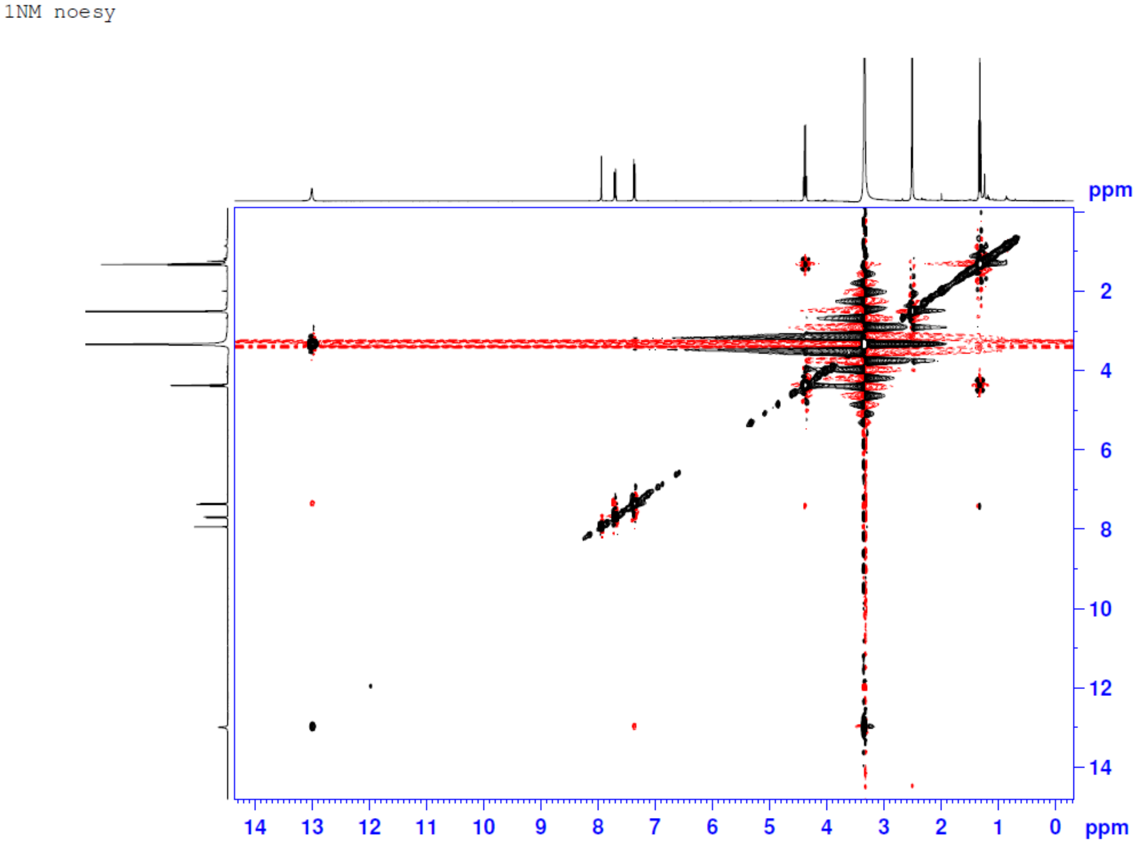

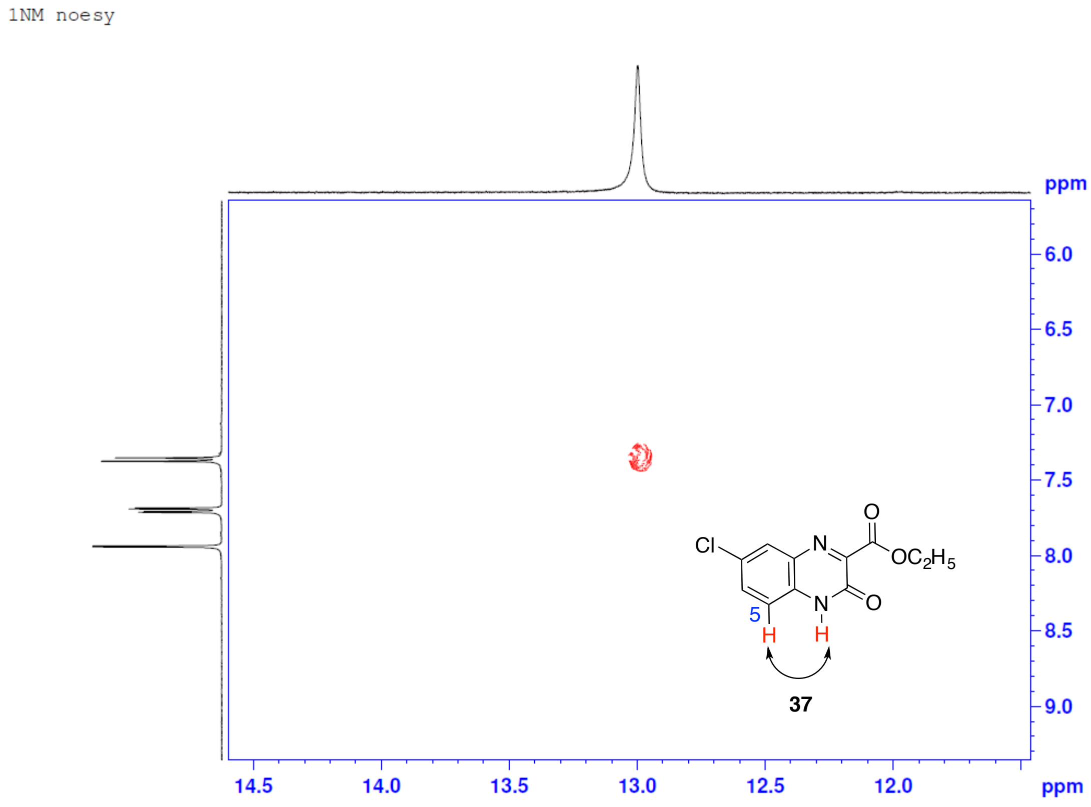

Figure S5. NOESY spectrum of isomer **38**

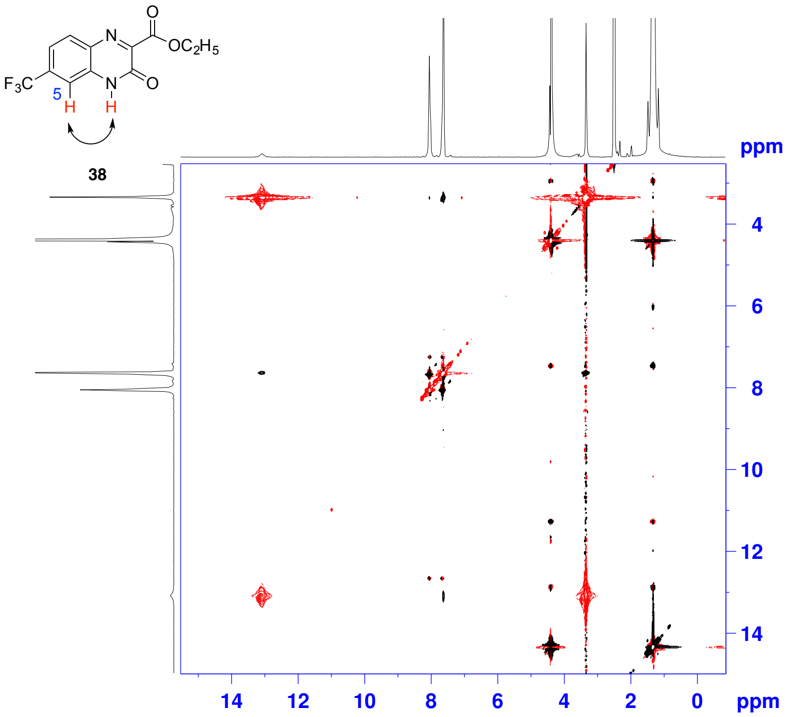

Figure S6. NOESY spectrum of isomer **39**

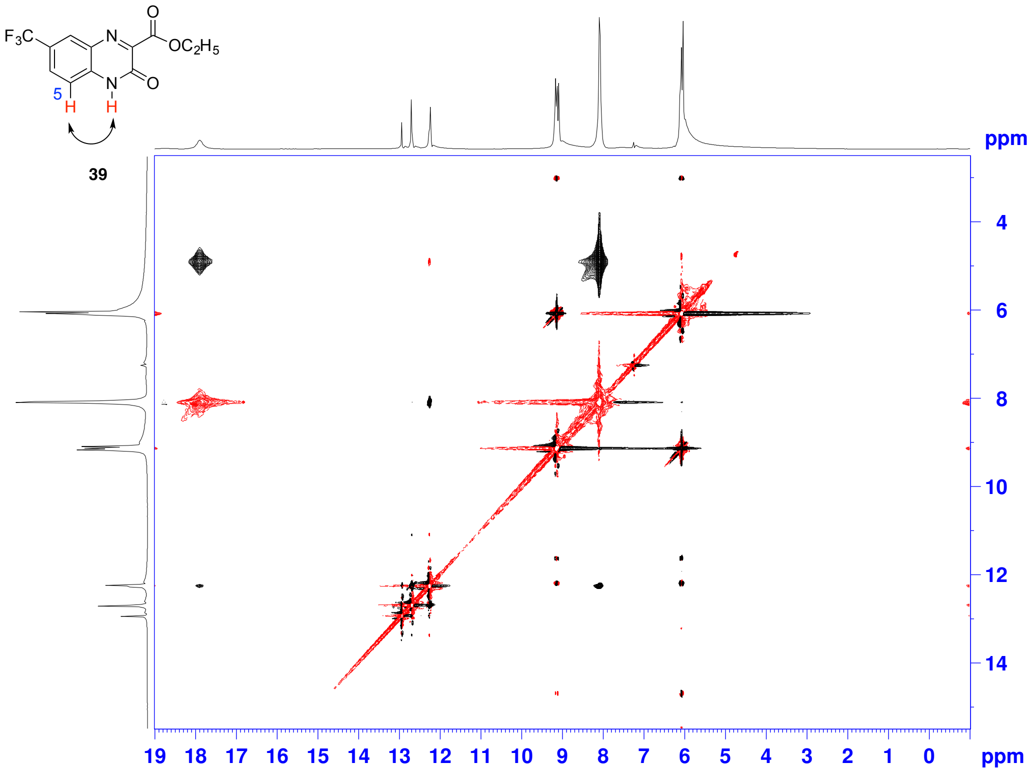

Figure S7. ^{1}H- and ^{13}C-NMR spectra of compound **4**

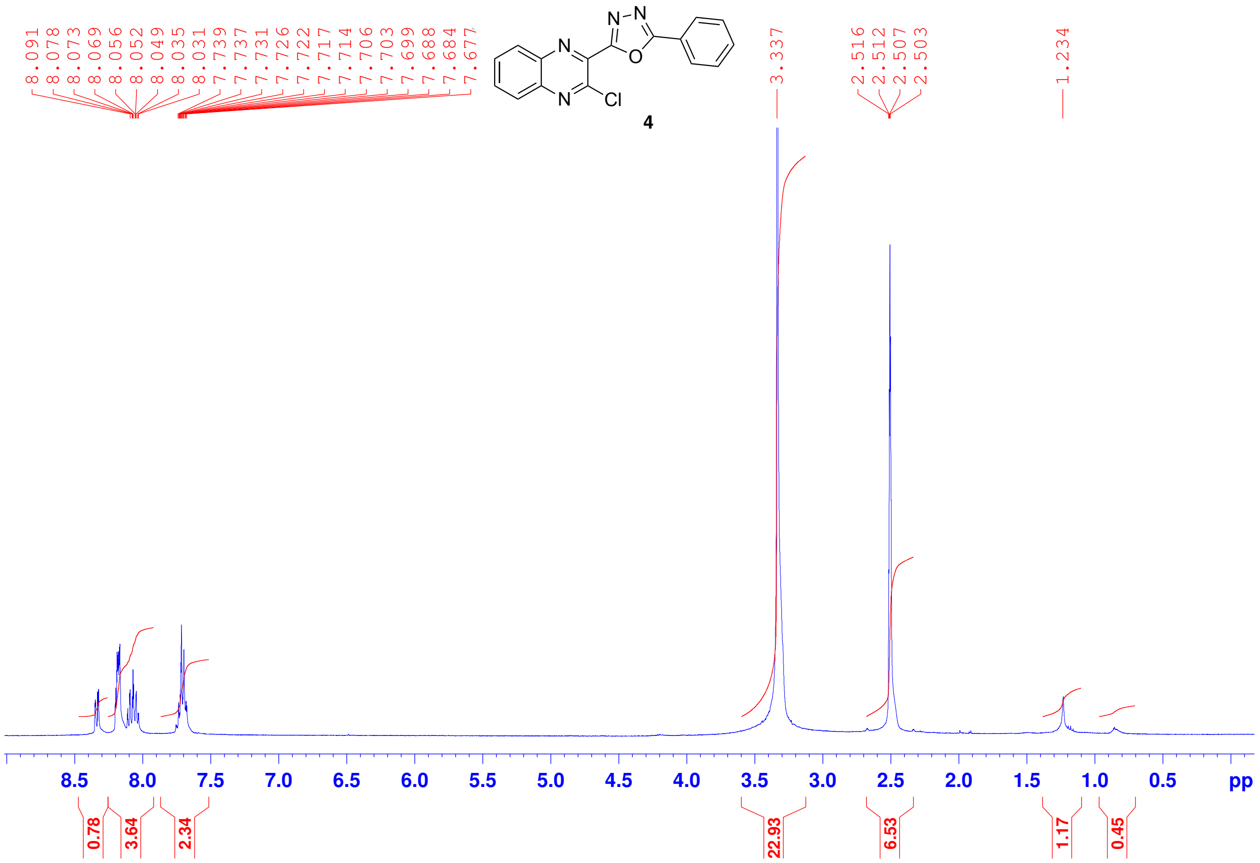

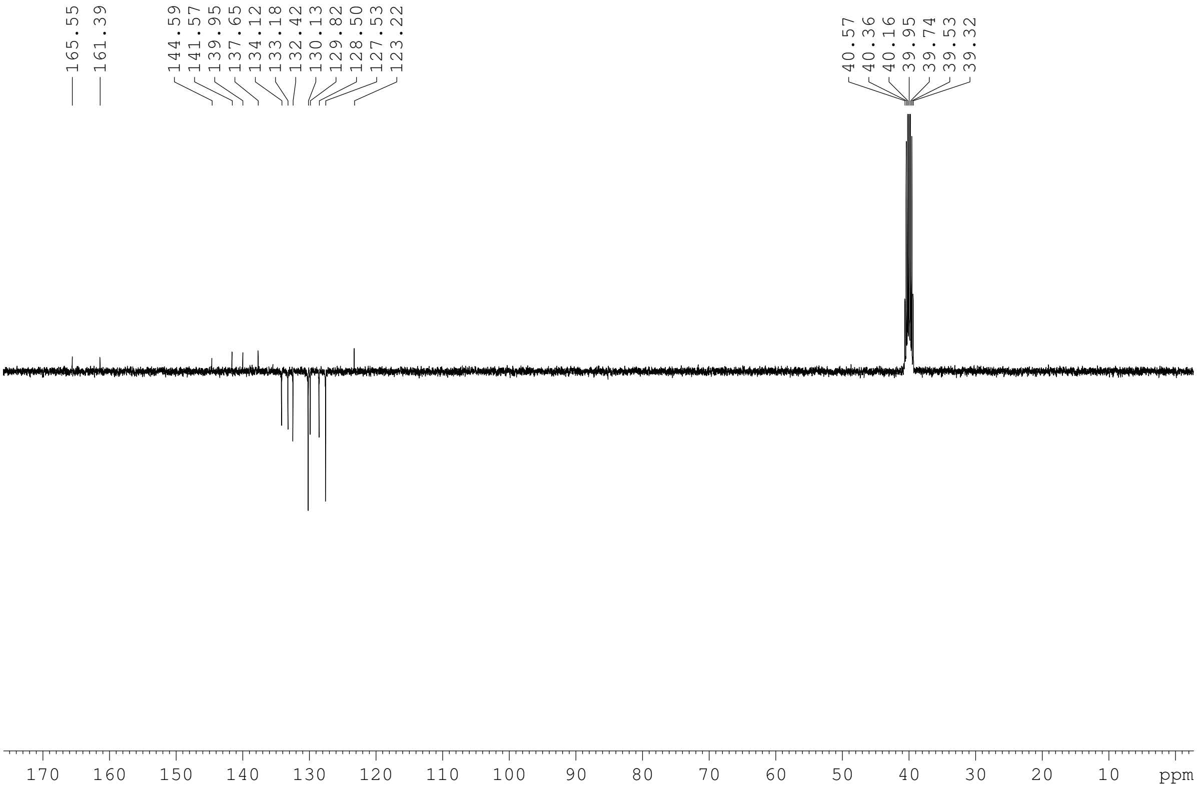

Figure S8. ^{1}H- and ^{13}C-NMR spectra of compound **5**

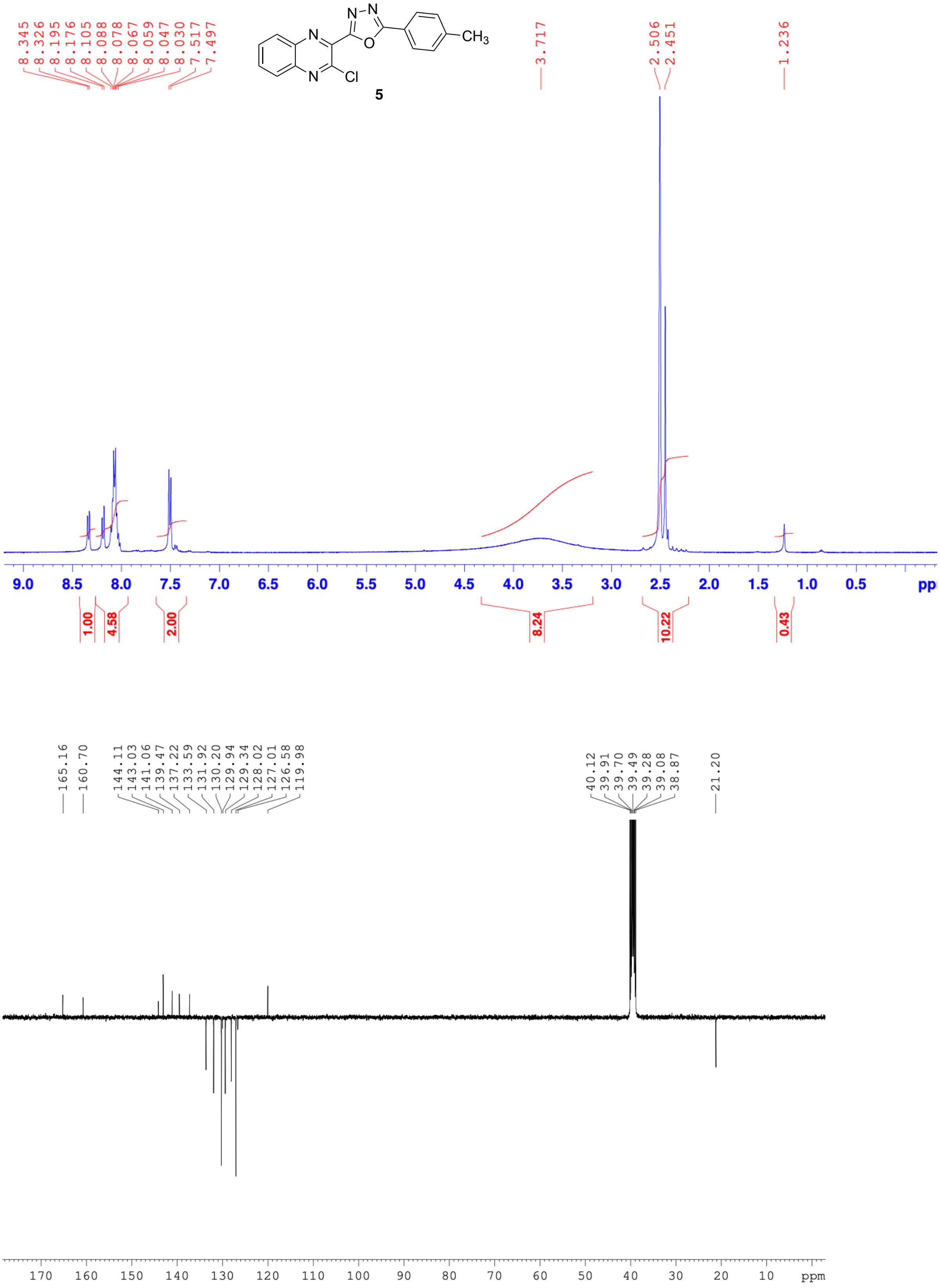

Figure S9. ^{1}H- and ^{13}C-NMR spectra of compound **6**

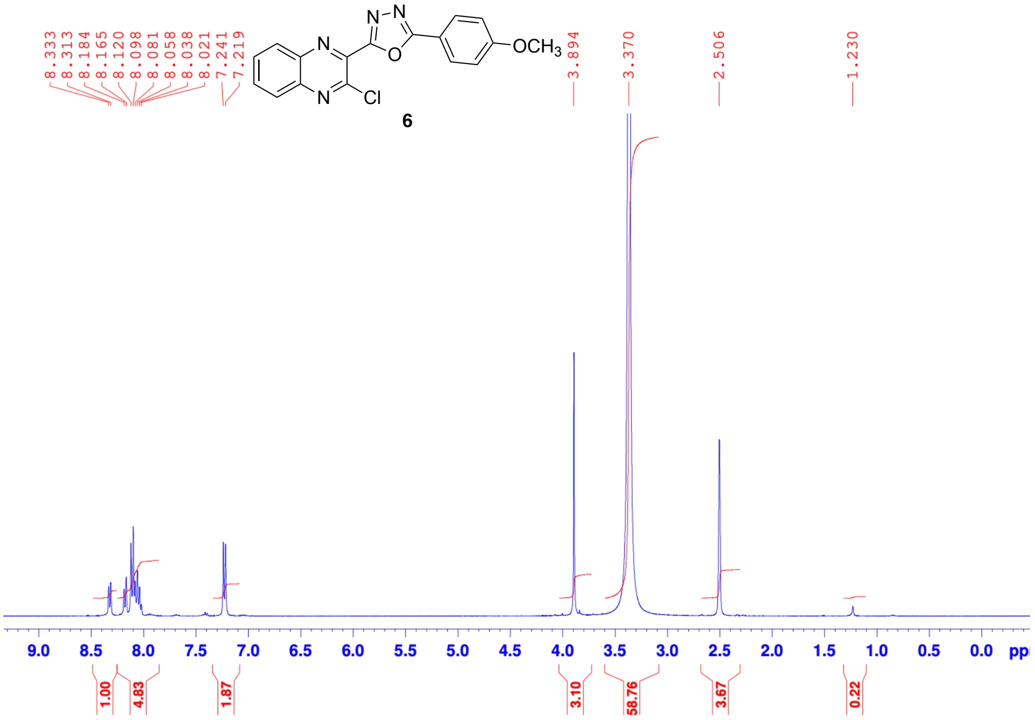

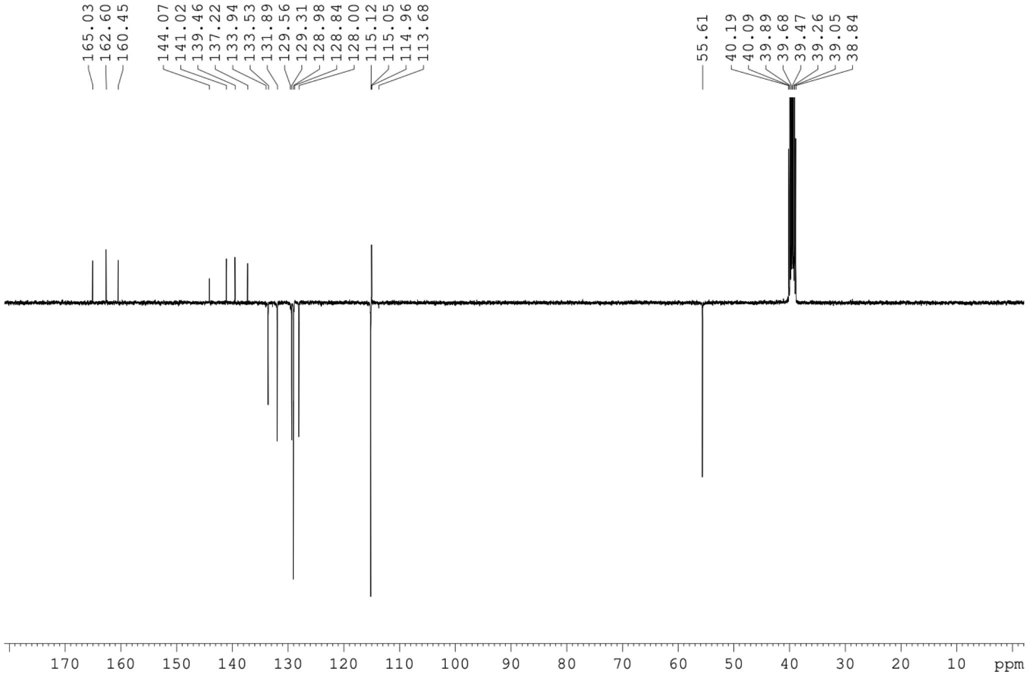

Figure S10. ^{1}H- and ^{13}C-NMR spectra of compound **7**

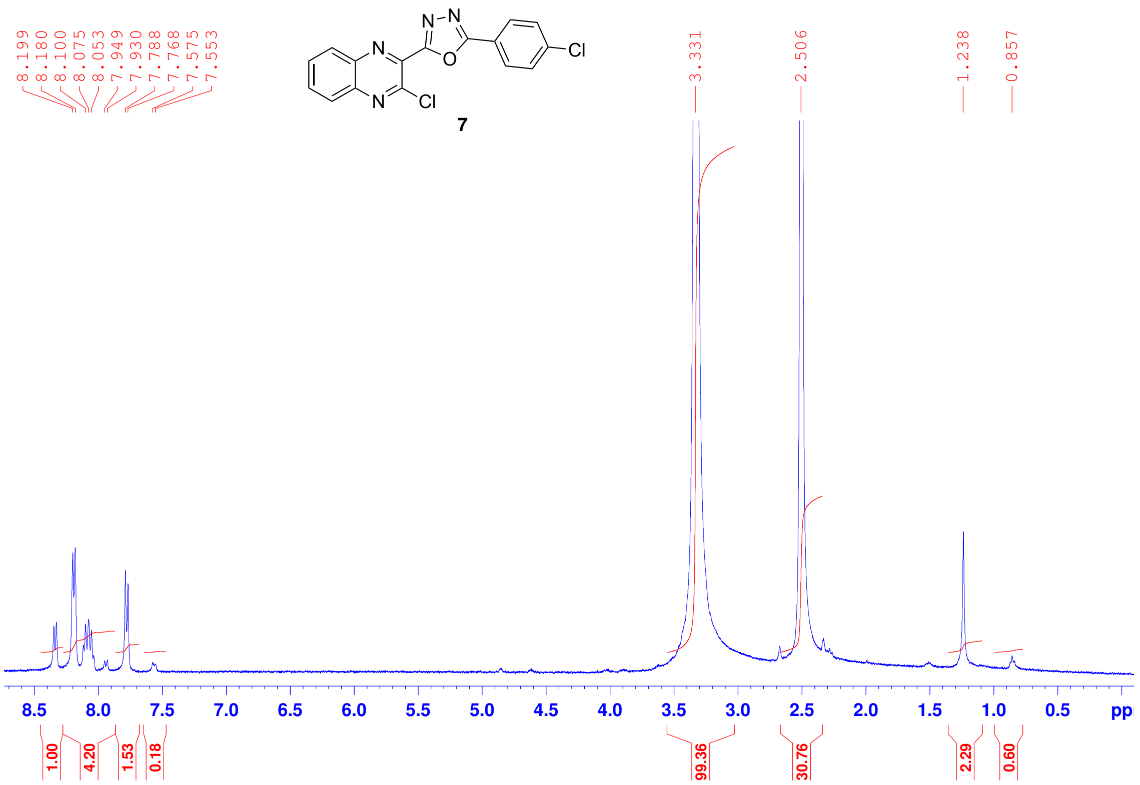

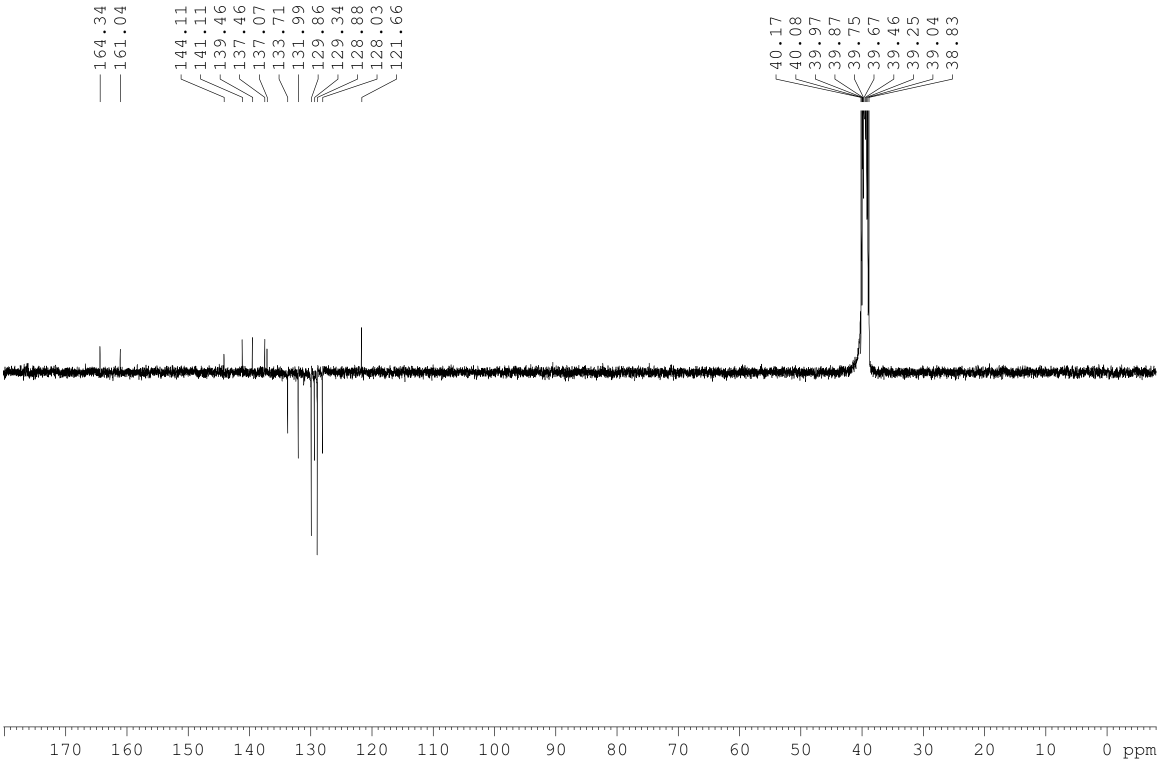

Figure S11. ^{1}H- and ^{13}C-NMR spectra of compound **8**

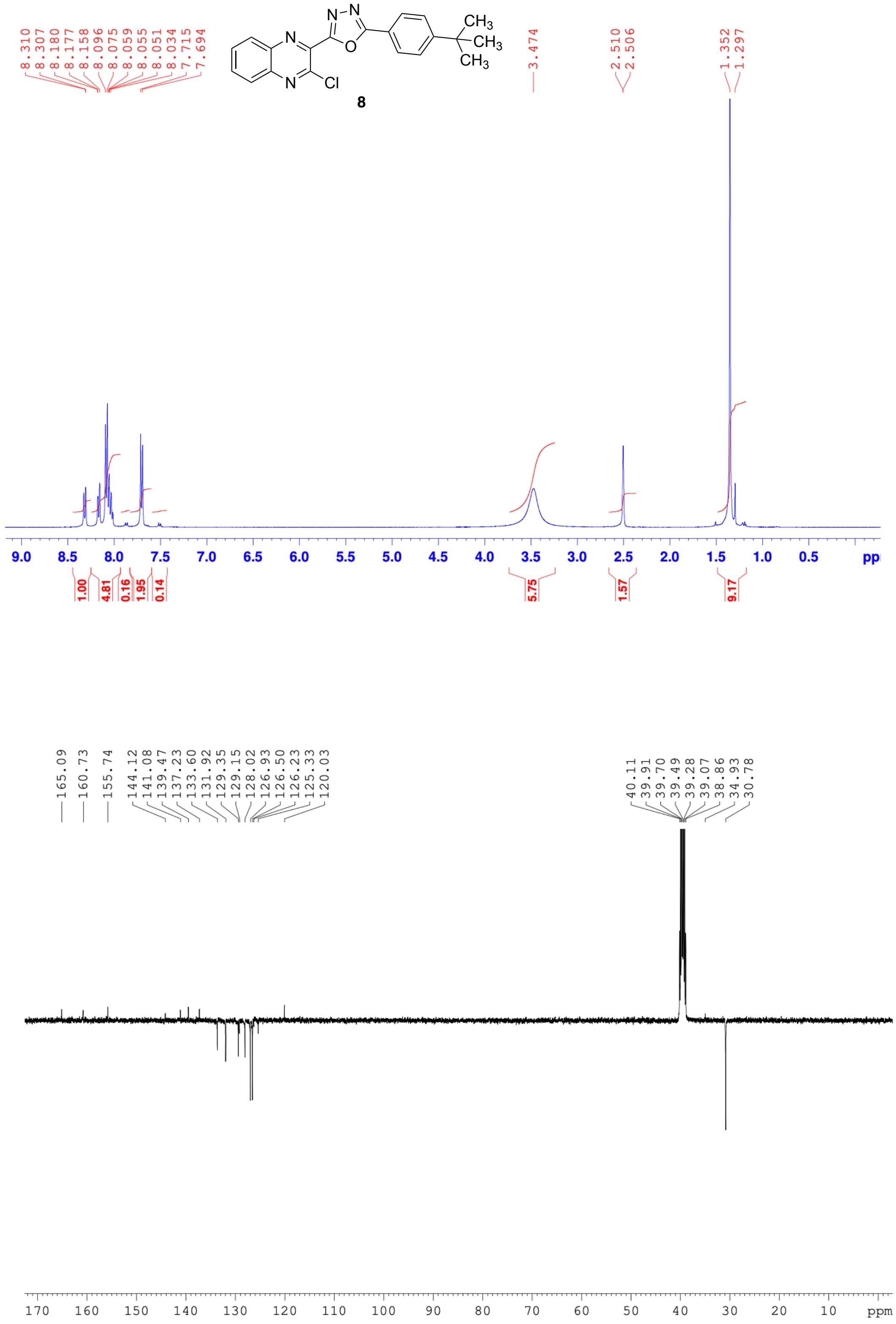

Figure S12. ^{1}H- and ^{13}C-NMR spectra of compound **9**

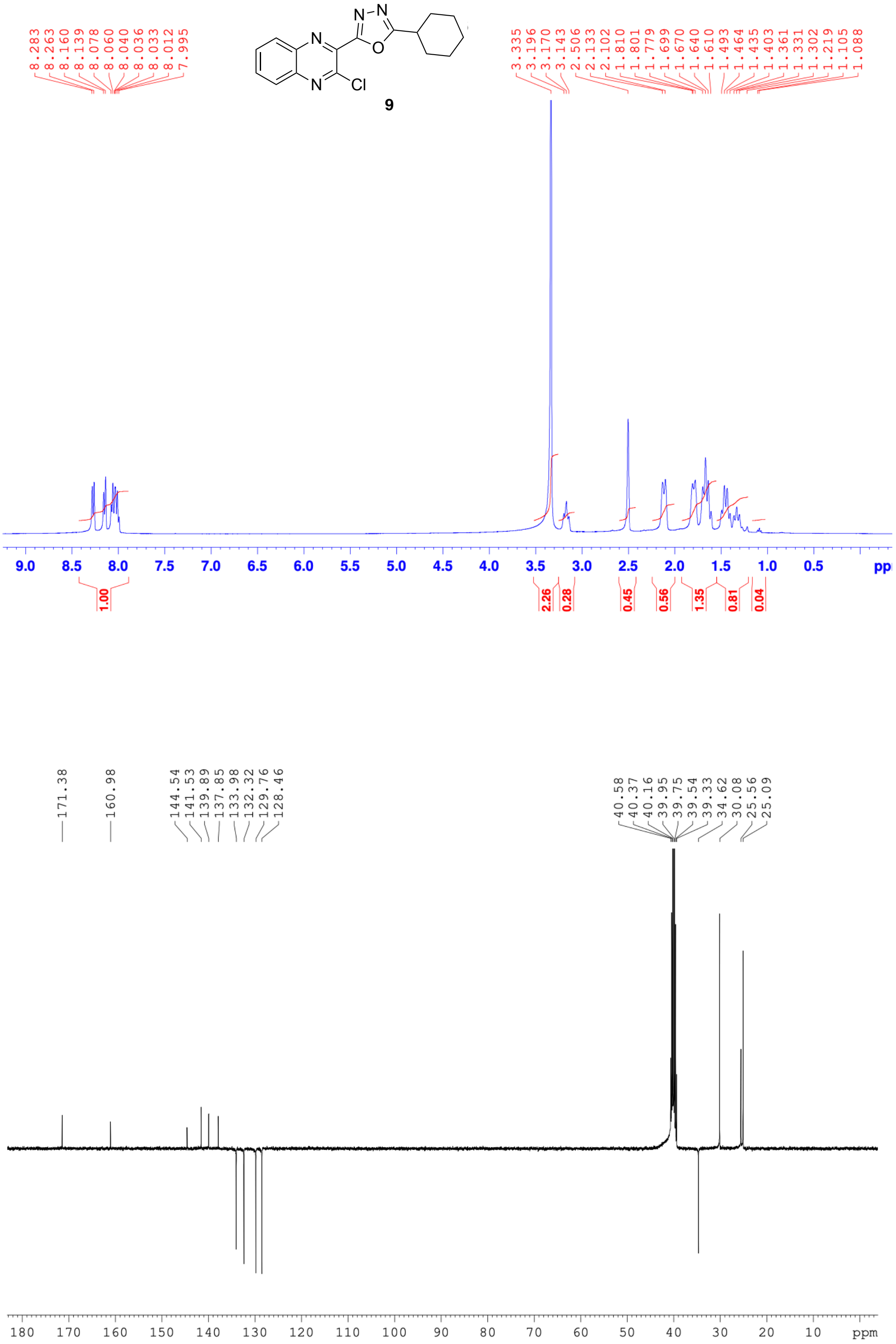

Figure S13. ^{1}H- and ^{13}C-NMR spectra of compound **10**

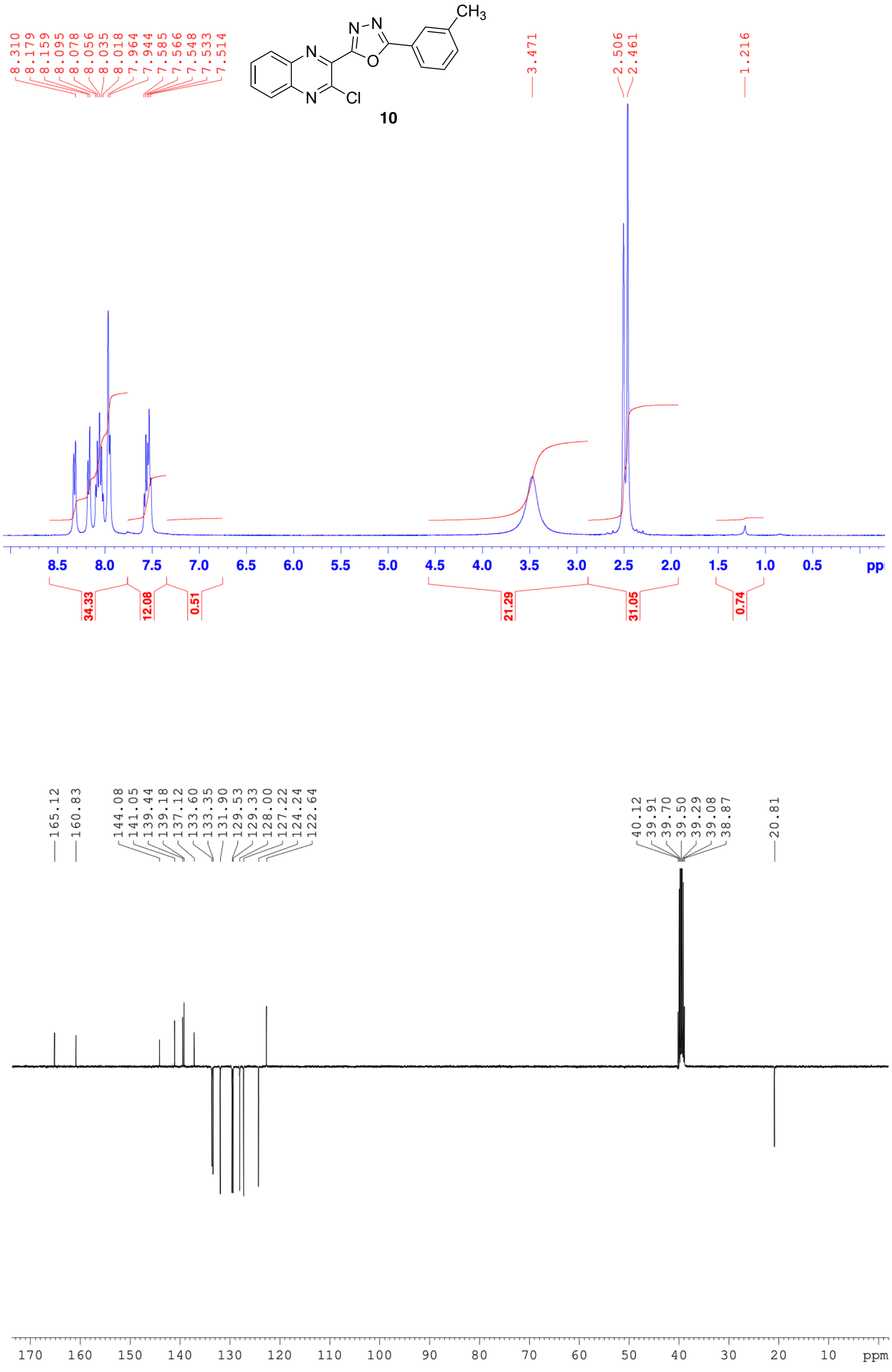

Figure S14. ¹H- and ¹³C-NMR spectra of compound **11**

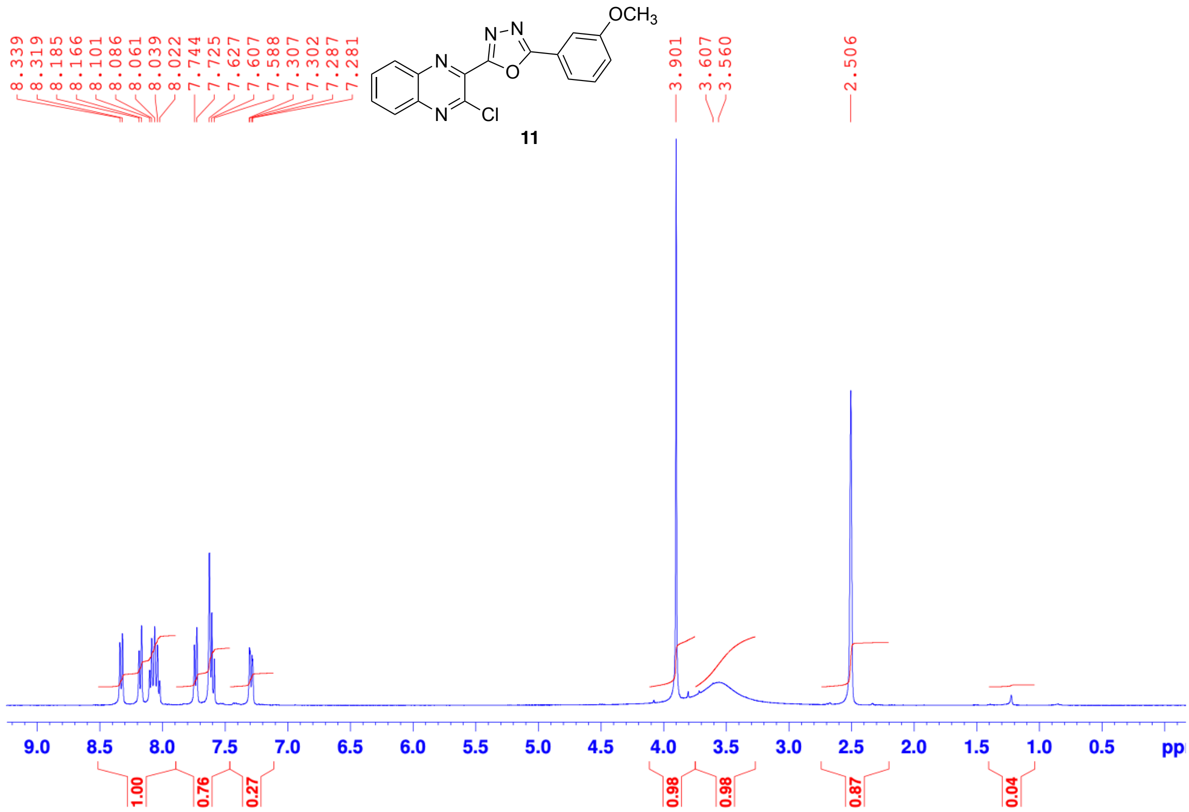

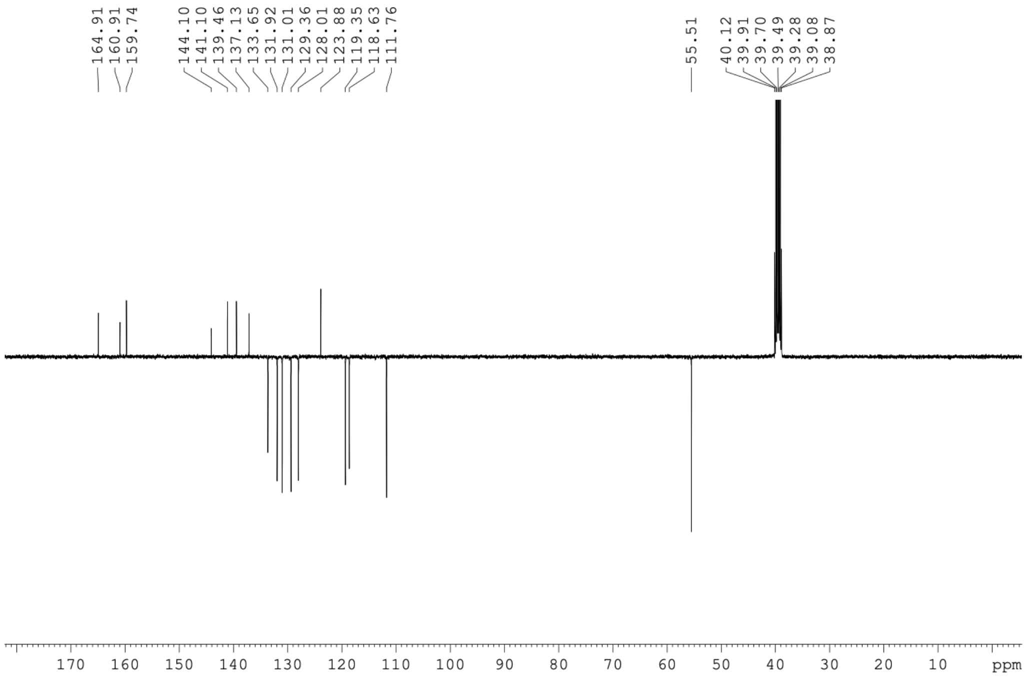

Figure S15. ^{1}H- and ^{13}C-NMR spectra of compound **12**

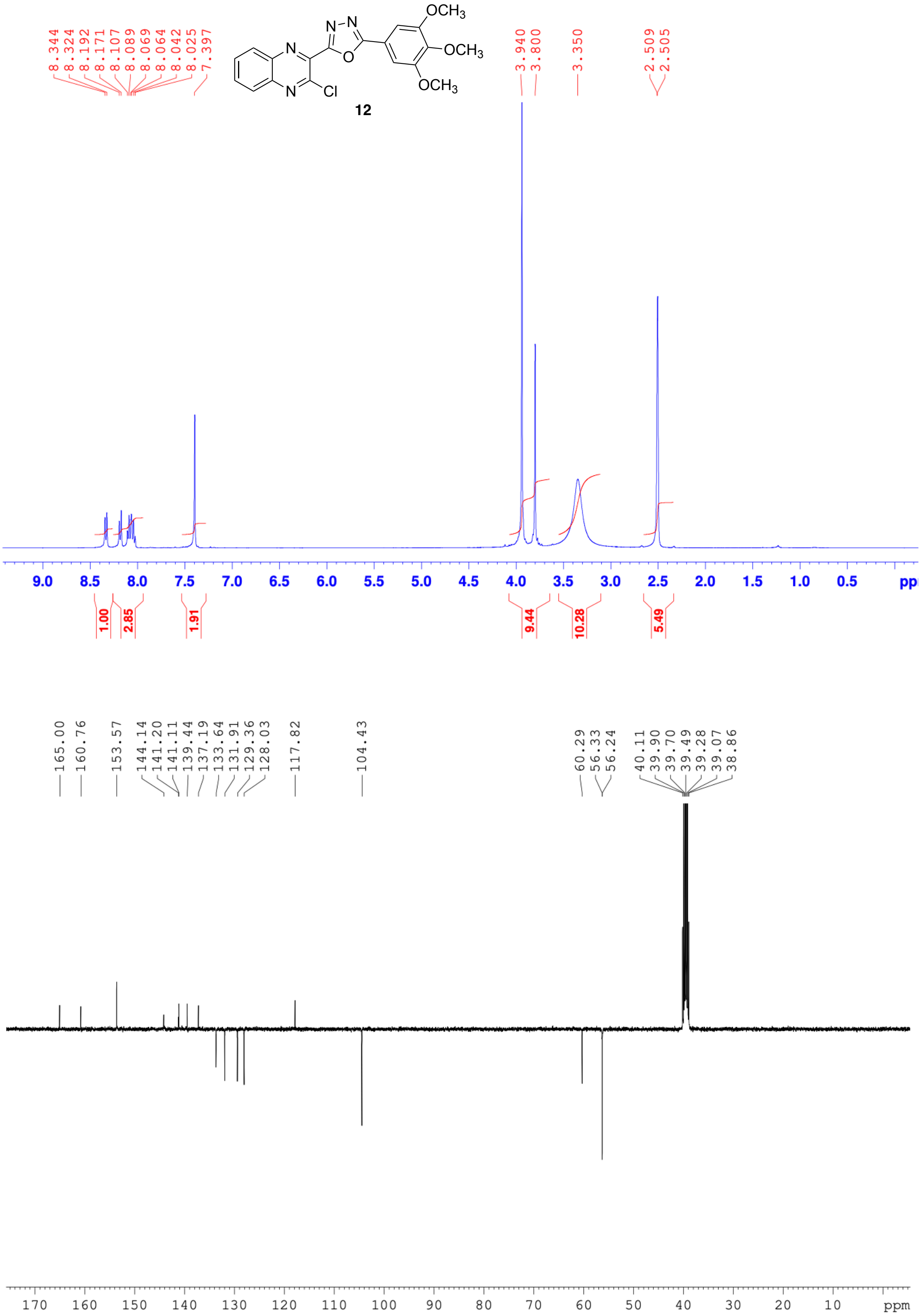

Figure S16. ^{1}H- and ^{13}C-NMR spectra of compound **13**

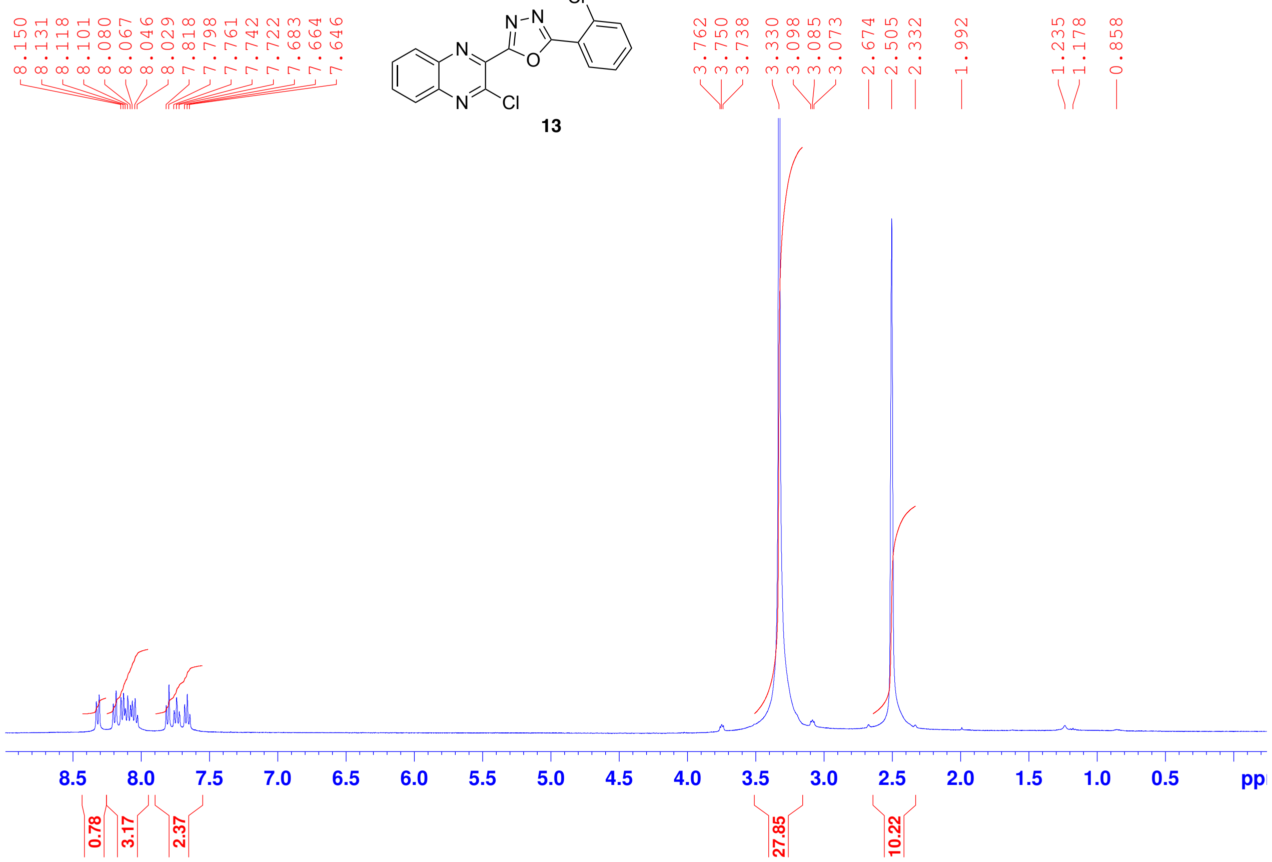

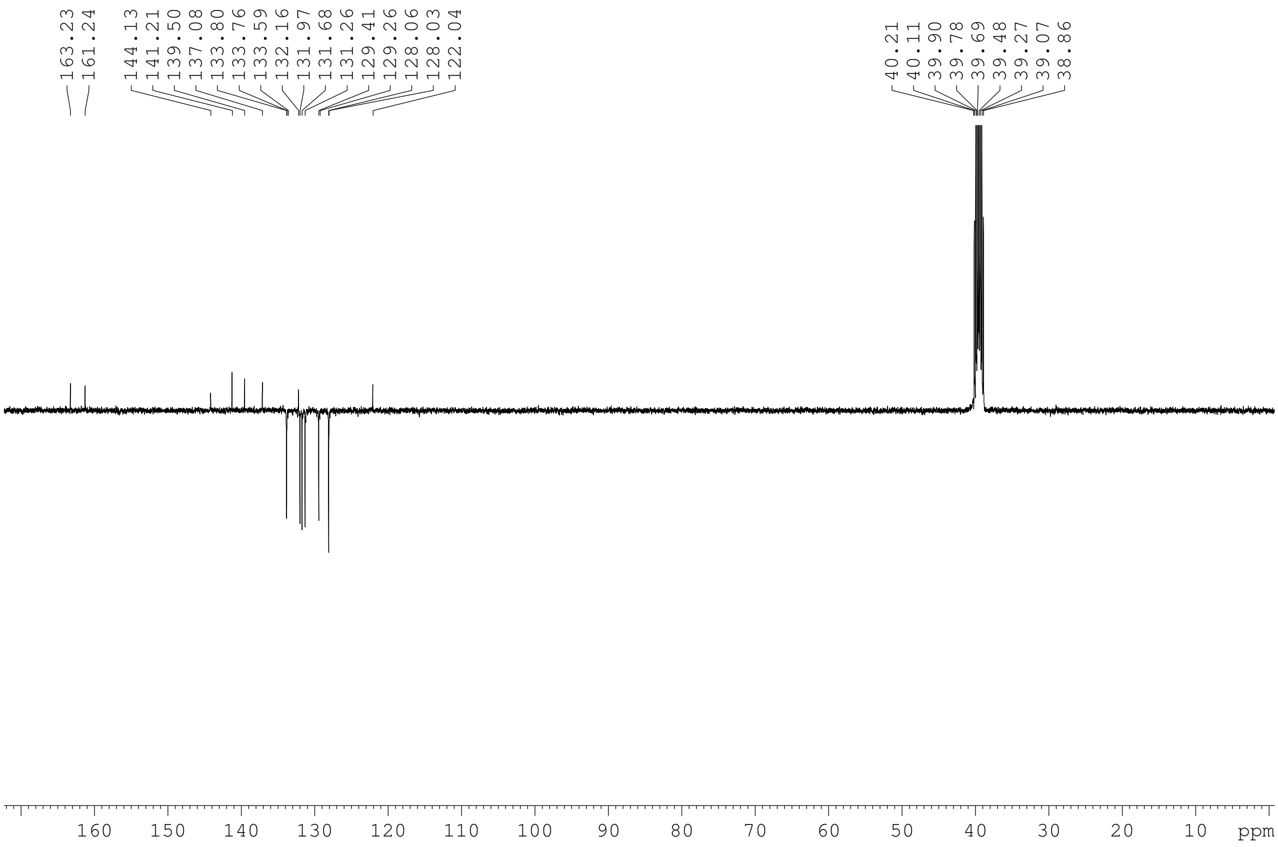

Figure S17. ^{1}H- and ^{13}C-NMR spectra of compound **14**

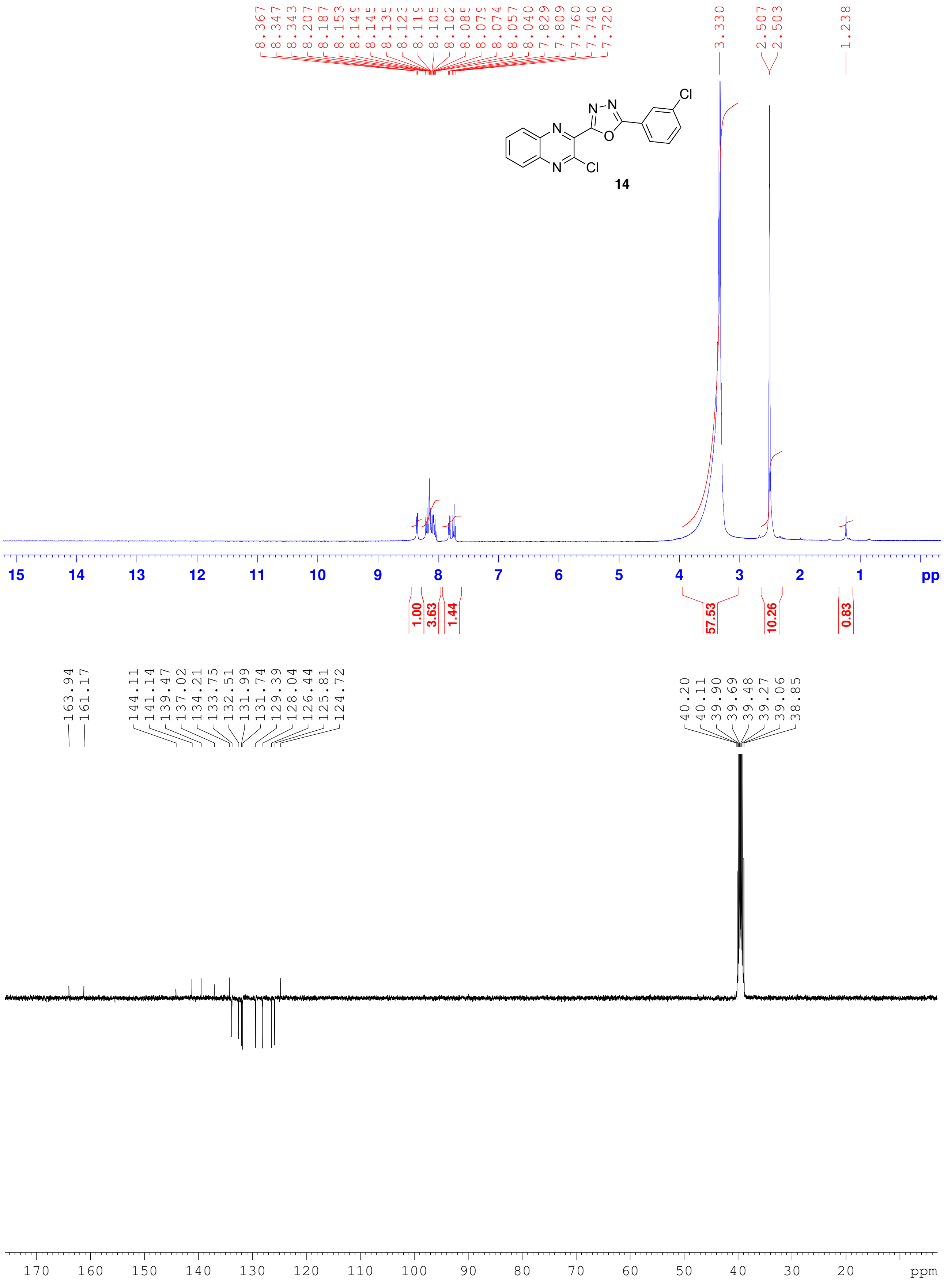

Figure S18. ^{1}H- and ^{13}C-NMR spectra of compound **15**

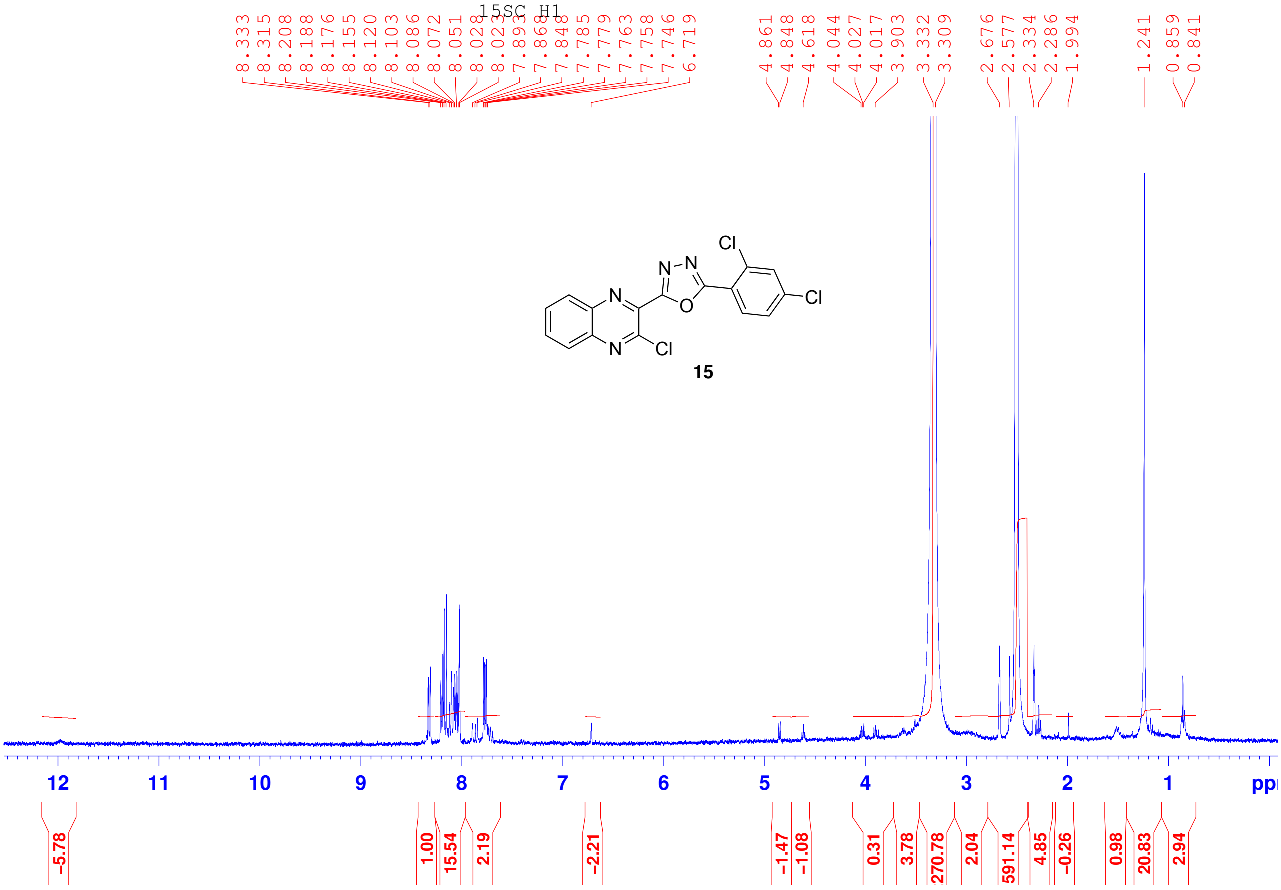

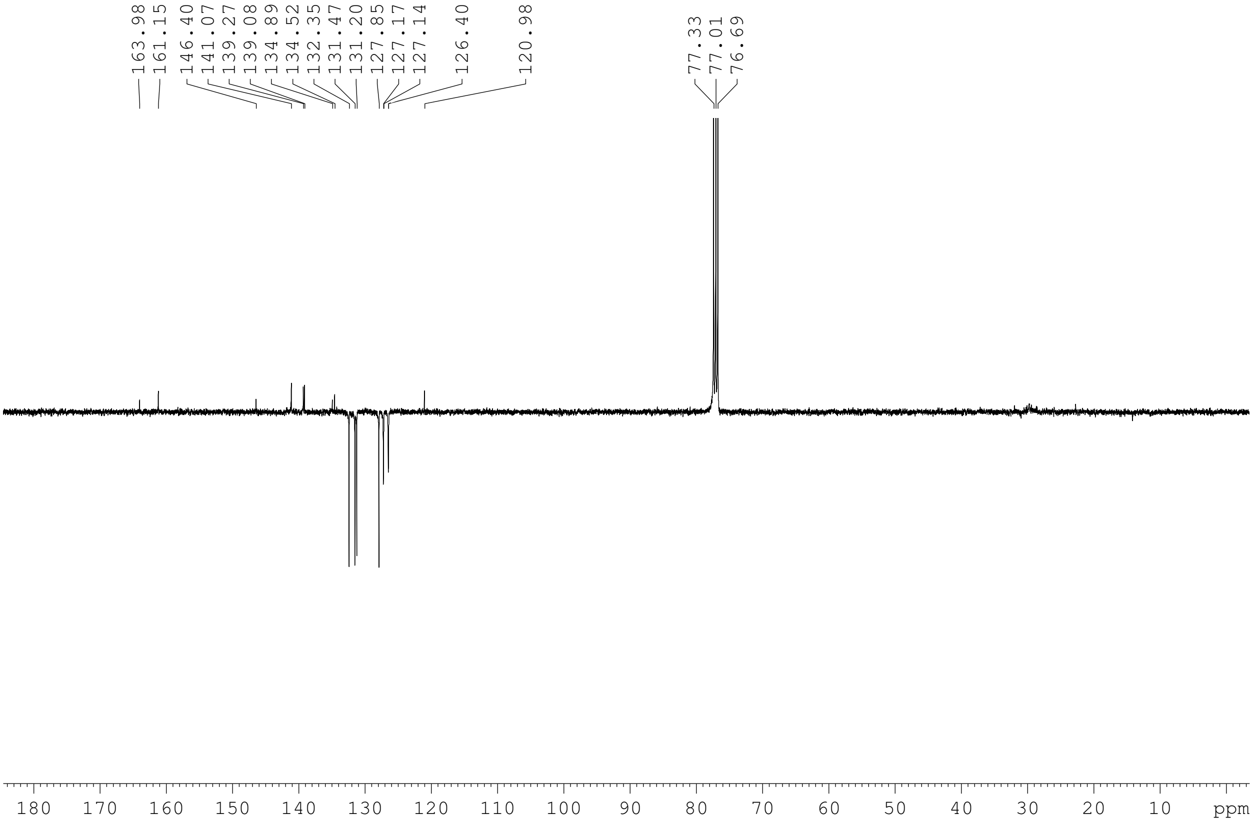

Figure S19. ^{1}H- and ^{13}C-NMR spectra of compound **16**

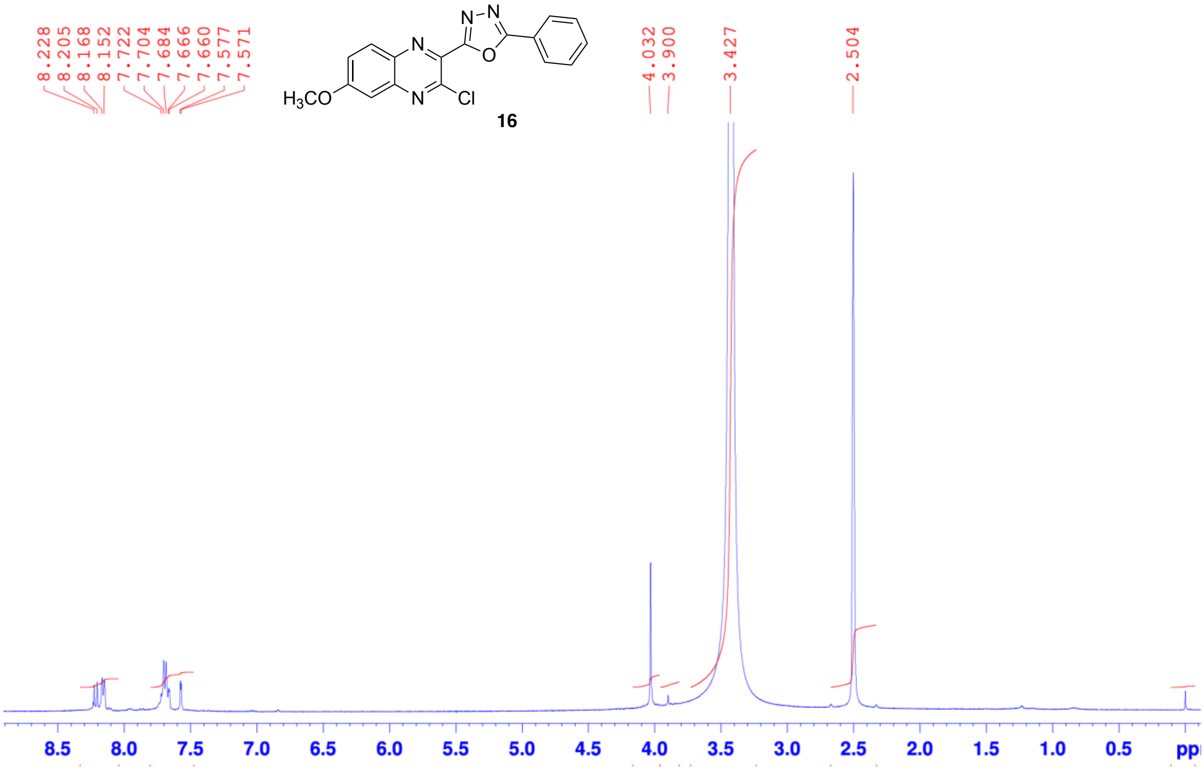

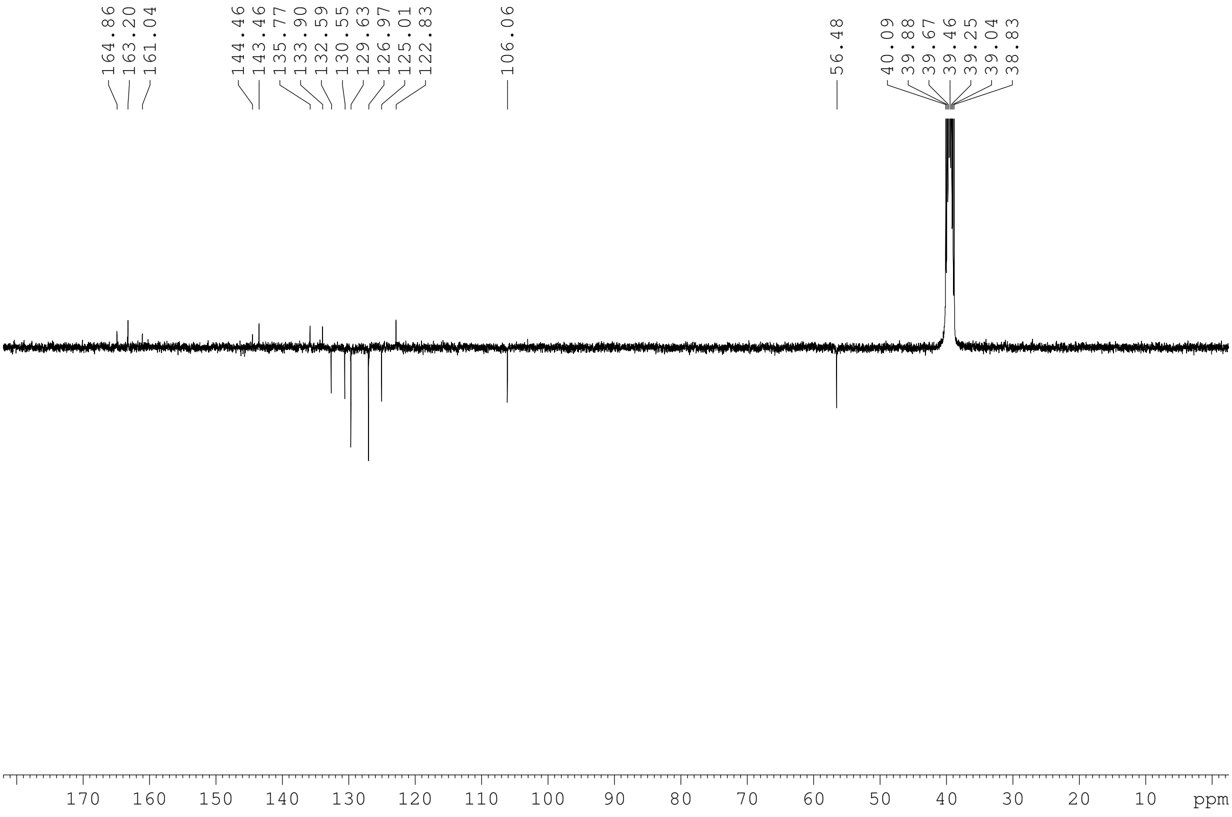

Figure S20. ^{1}H- and ^{13}C-NMR spectra of compound **17**

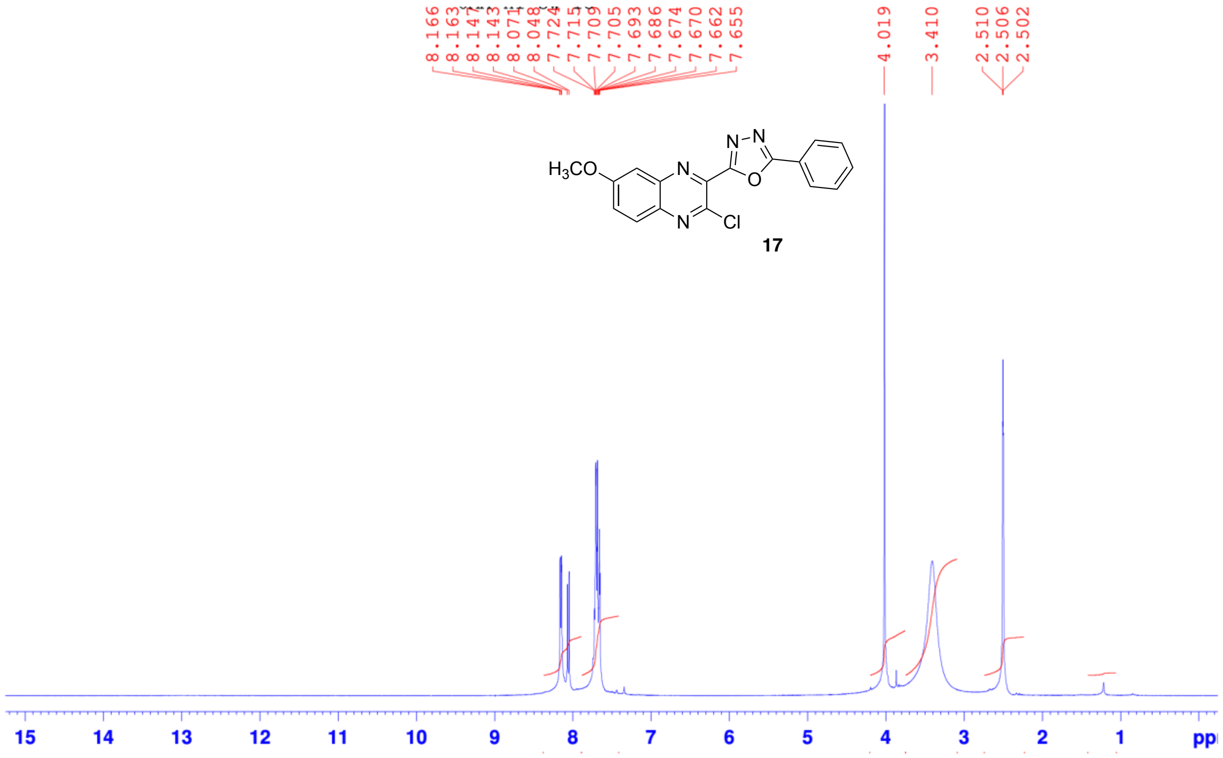

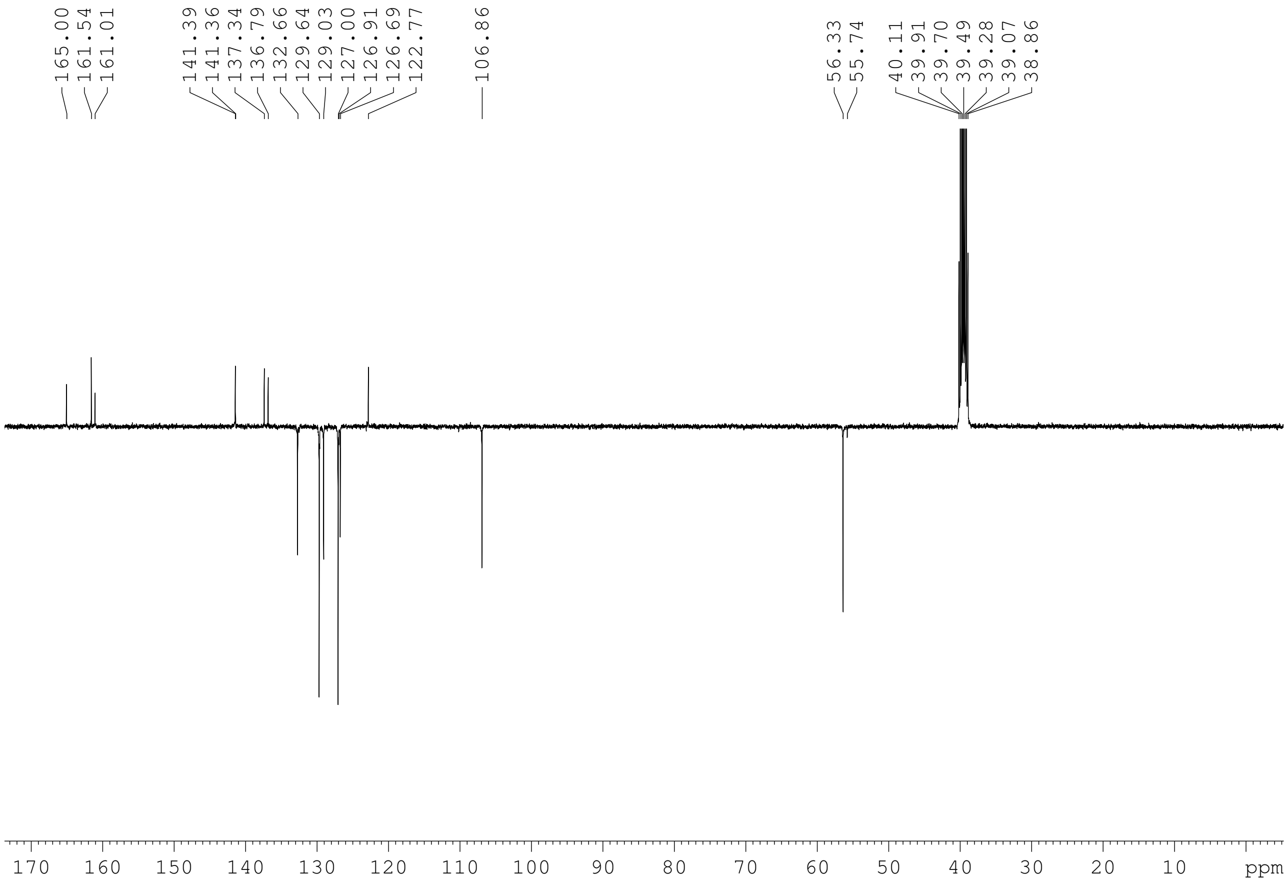

Figure S21. ^{1}H- and ^{13}C-NMR spectra of compound **18**

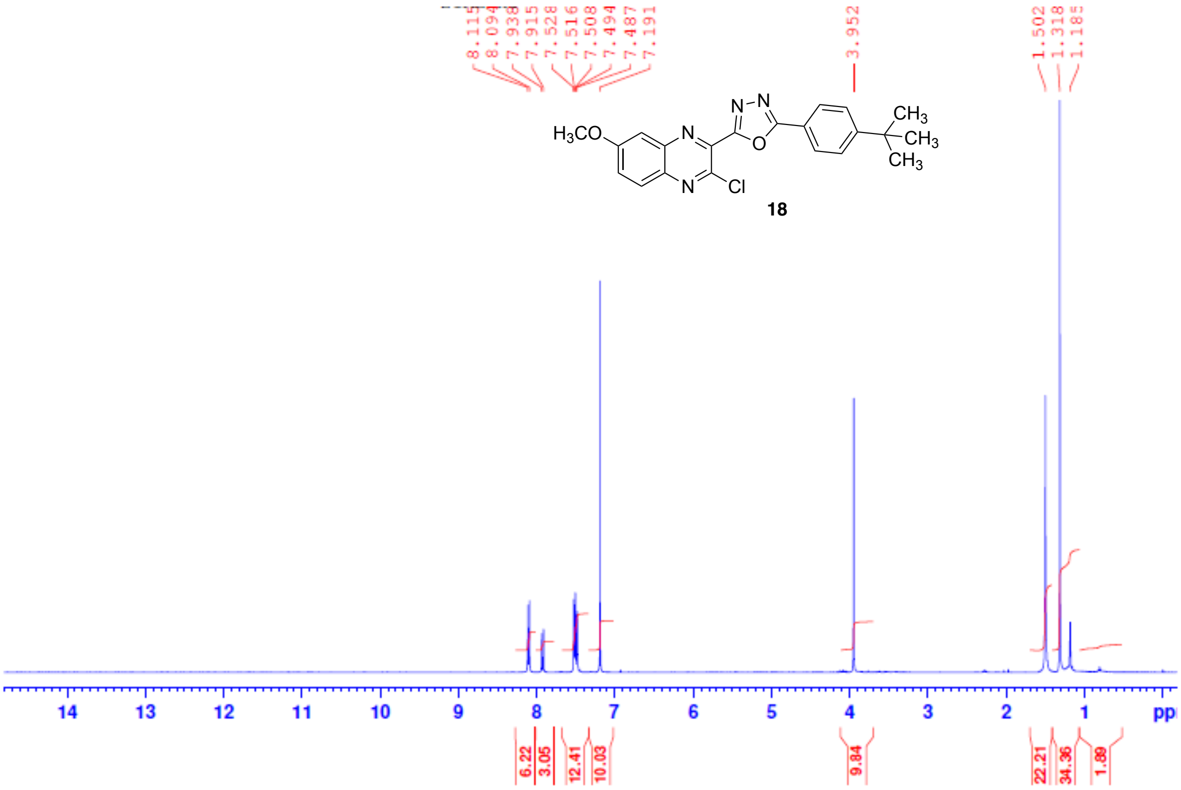

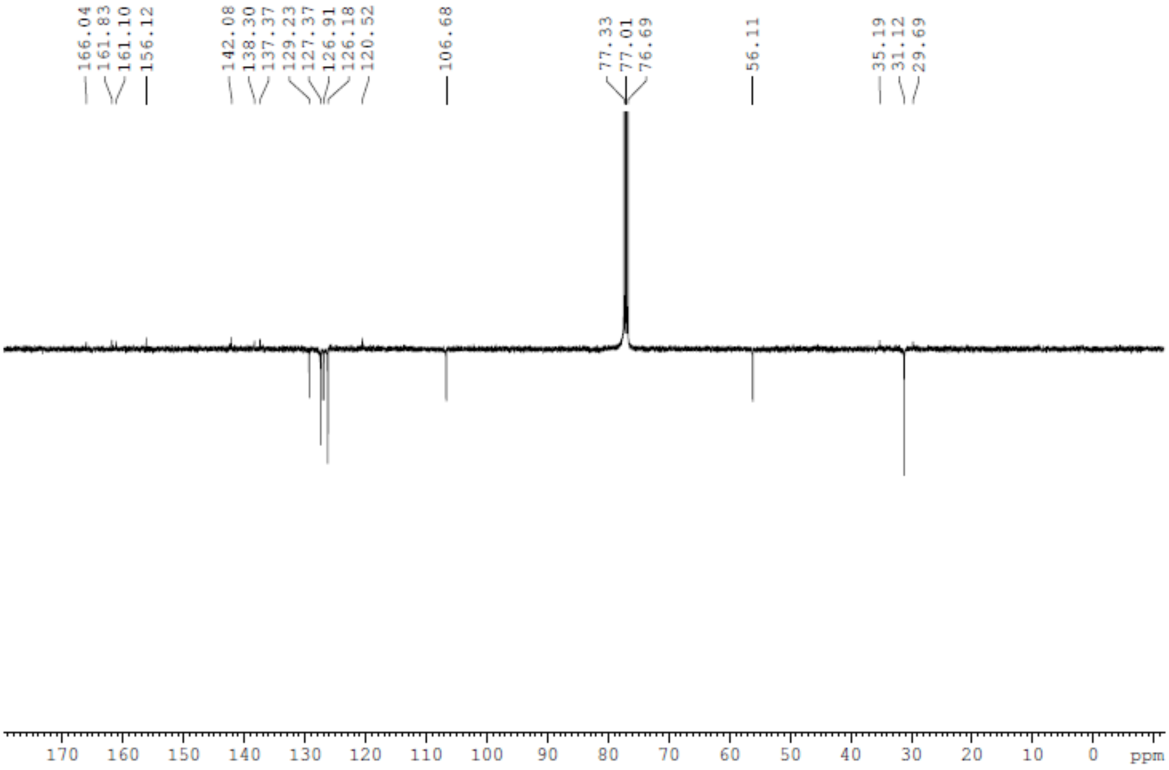

Figure S22. ^{1}H- and ^{13}C-NMR pectra of compound **19**

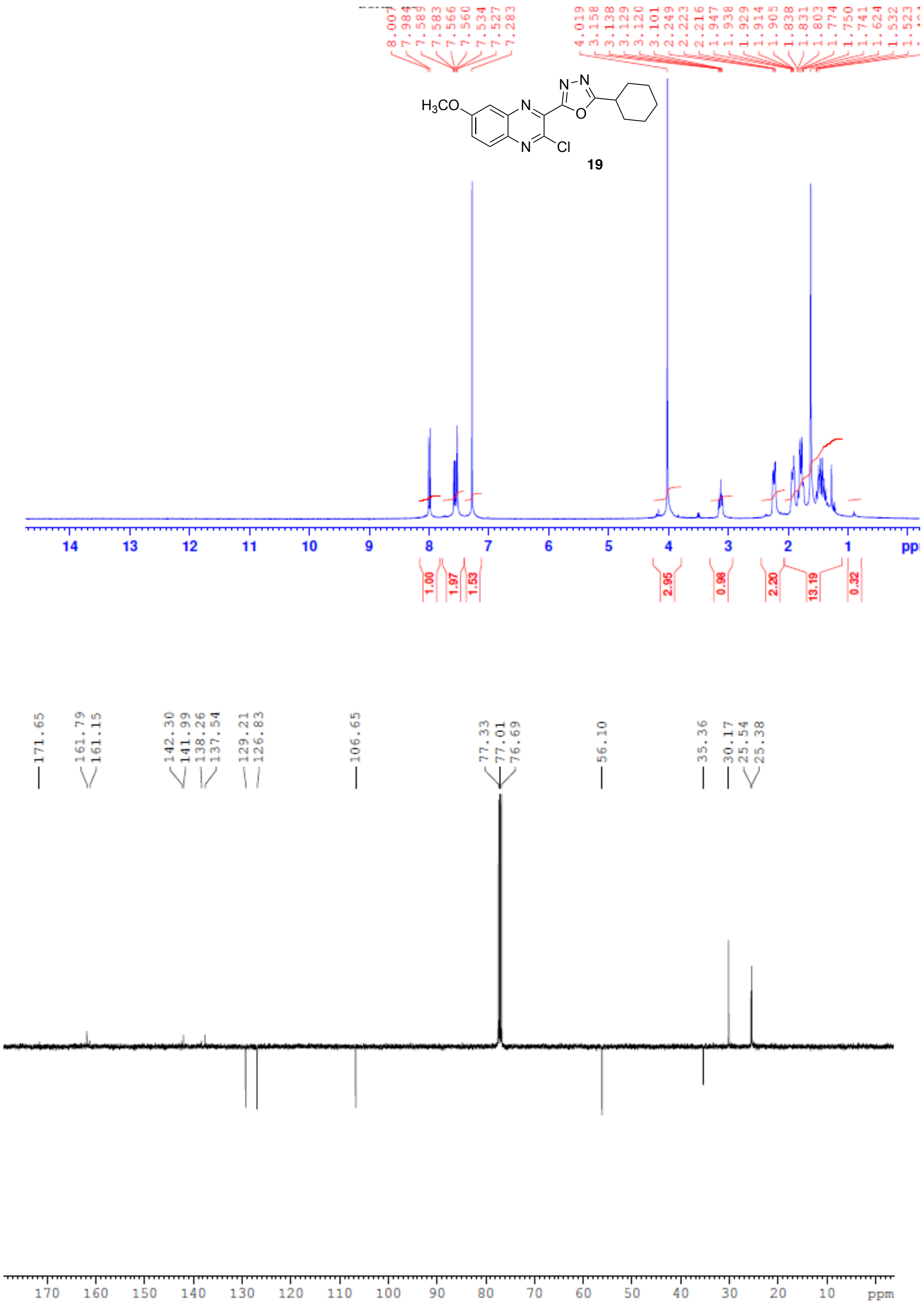

Figure S23. ^{1}H- and ^{13}C-NMR spectra of compound **20**

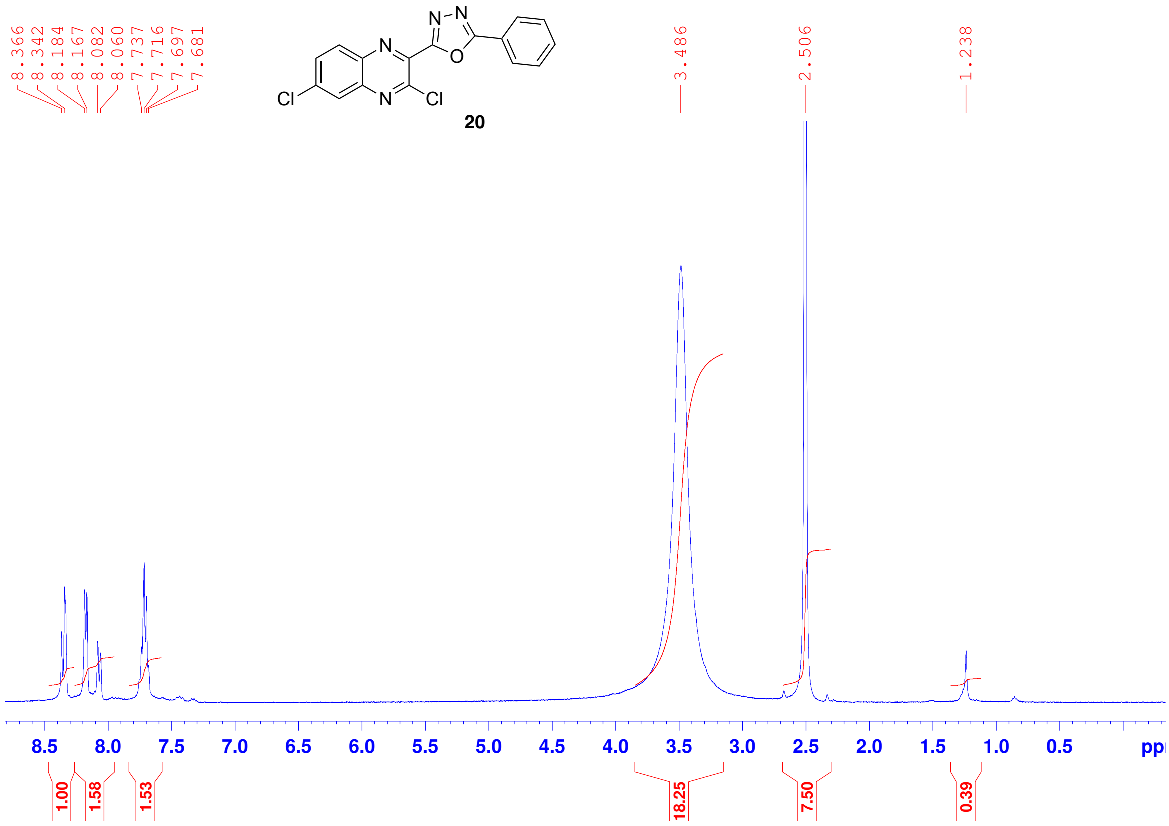

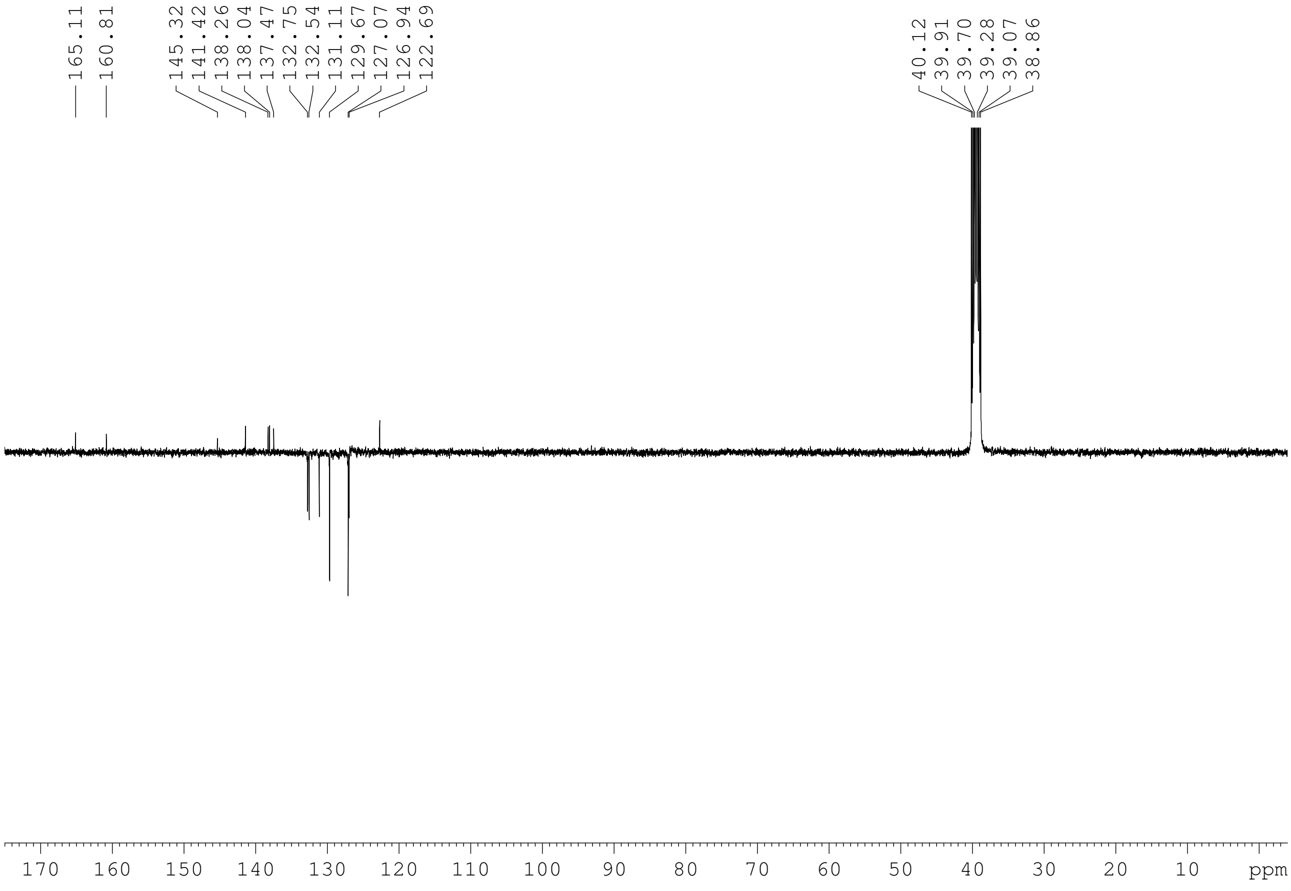

Figure S24. ^{1}H- and ^{13}C-NMR spectra of compound **21**

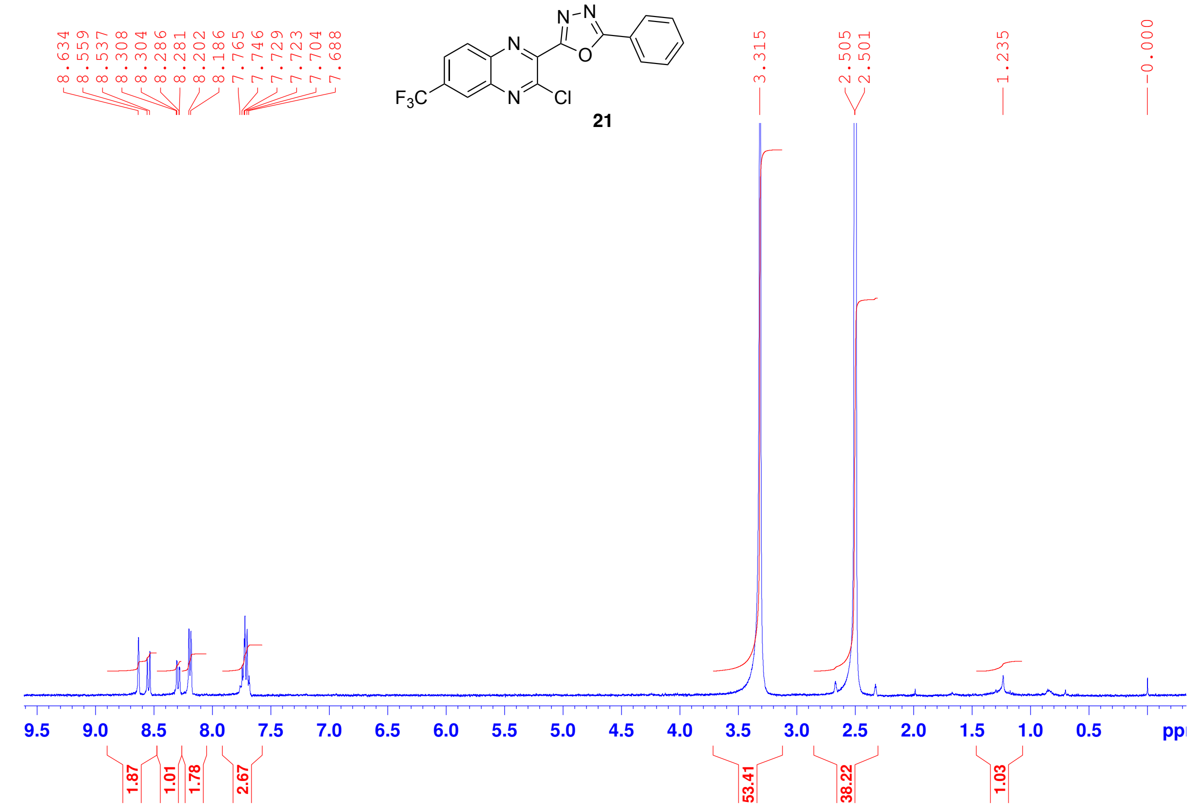

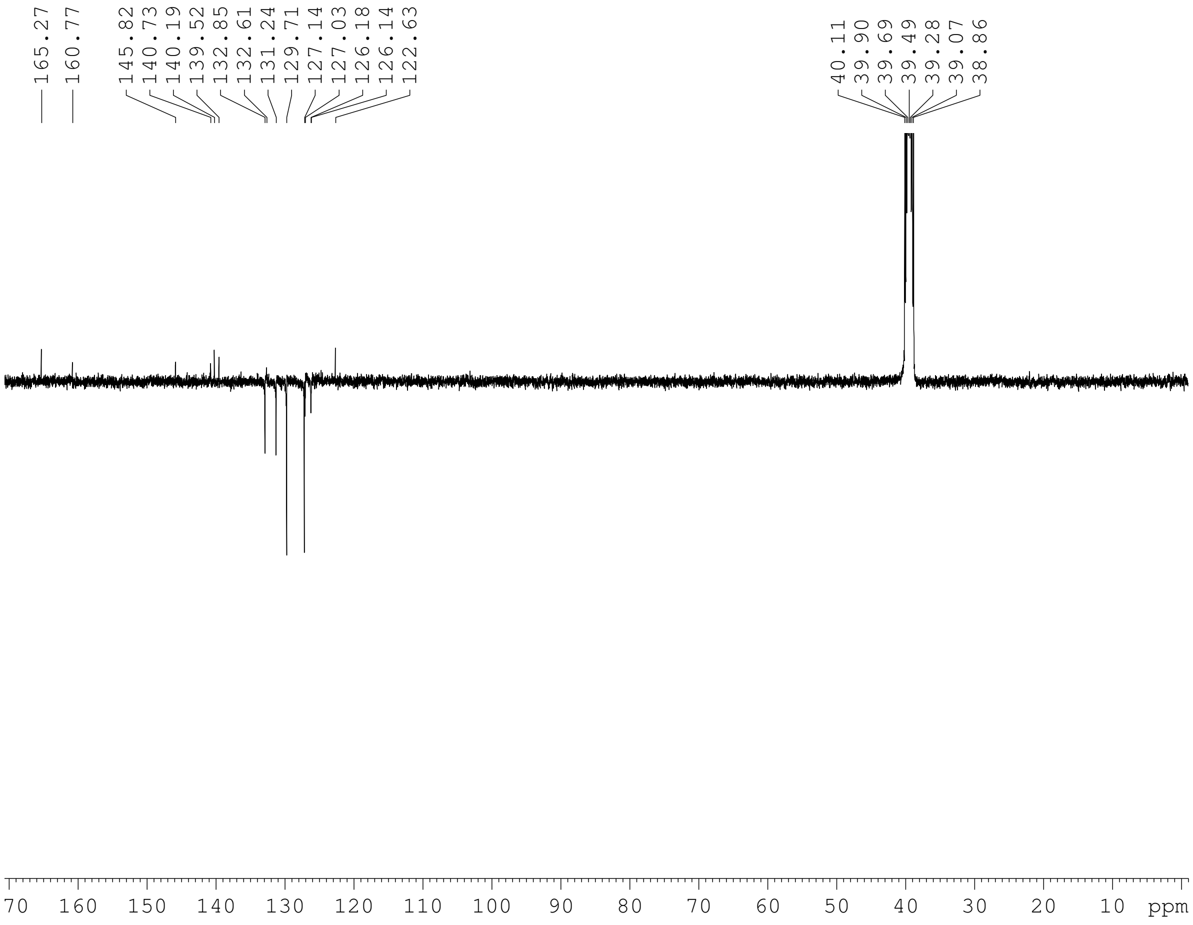

Figure S25. ^{1}H- and ^{13}C-NMR spectra of compound **22**

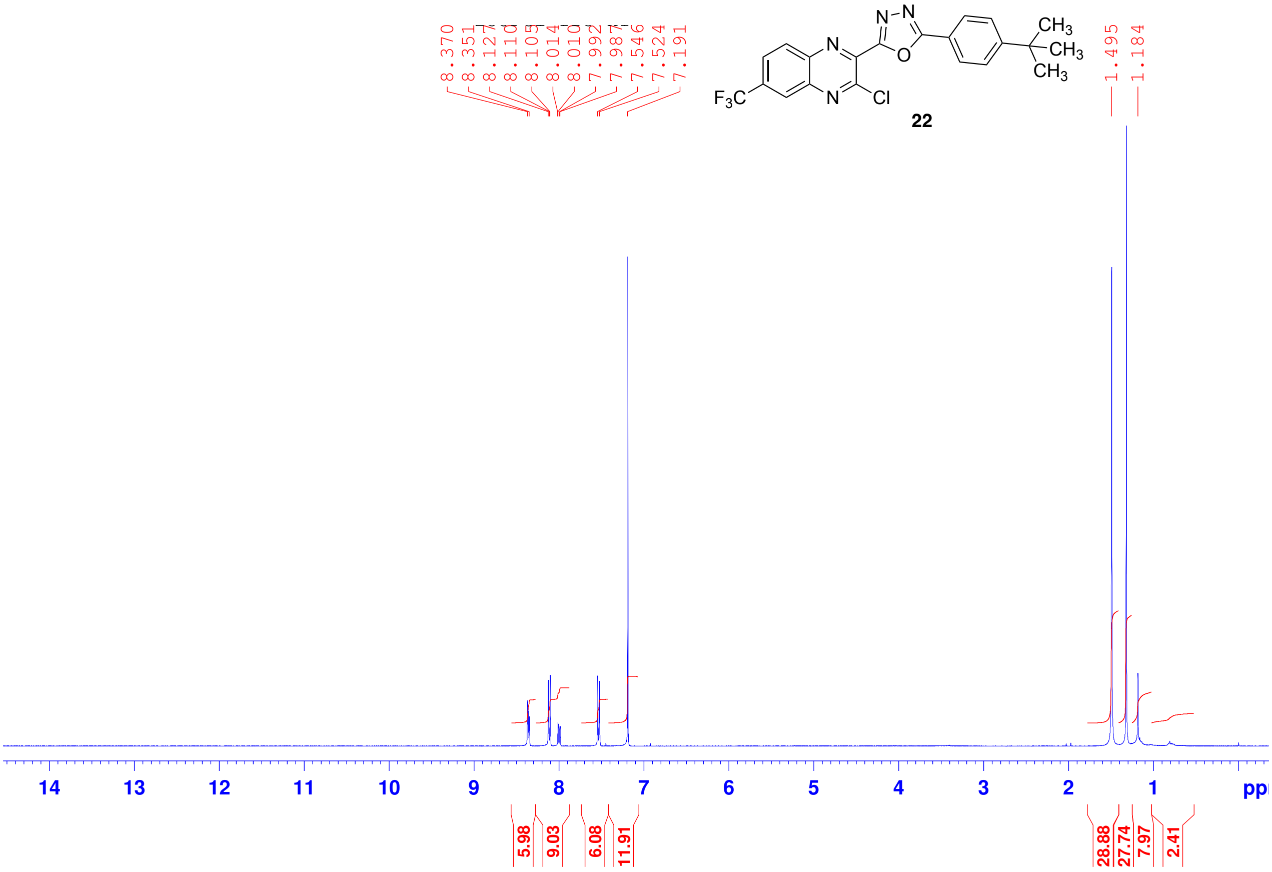

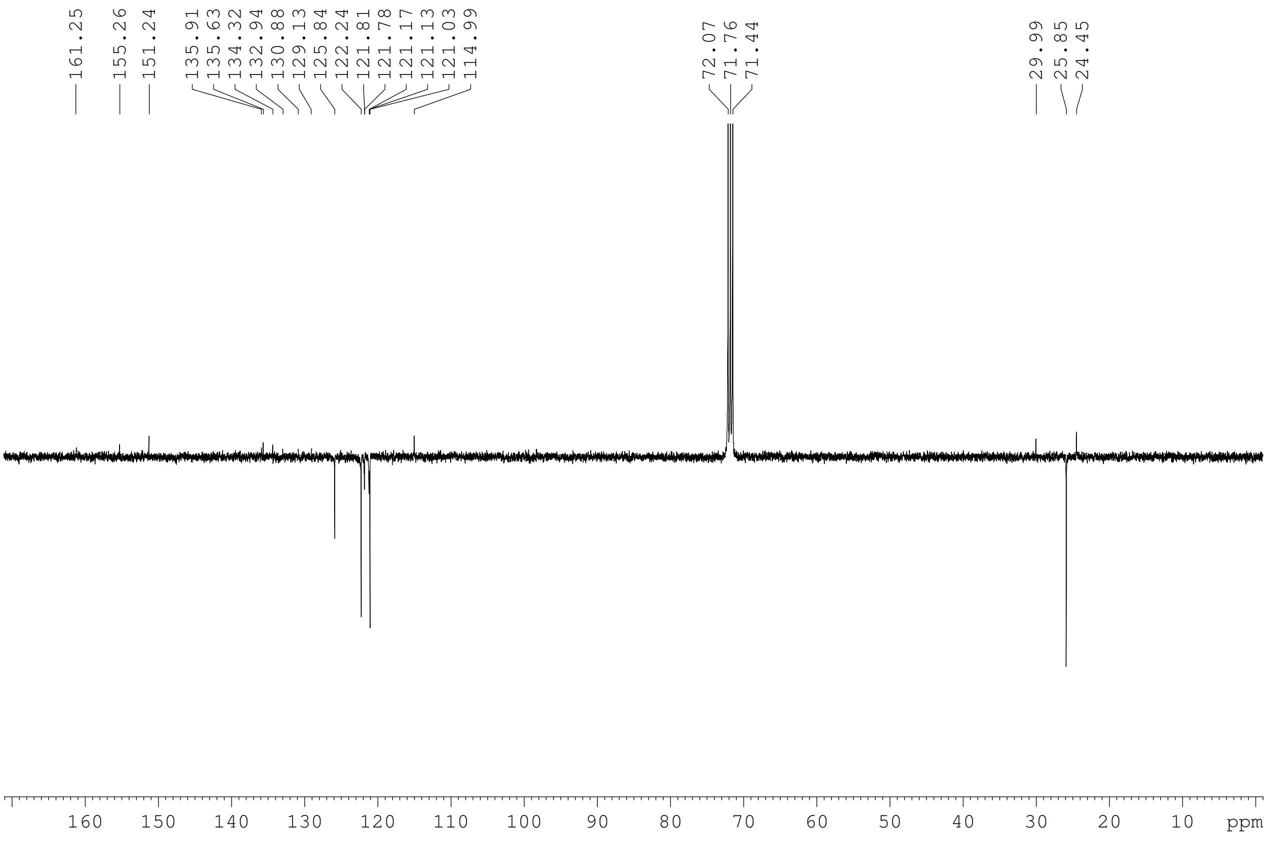

Figure S26. ^{1}H- and ^{13}C-NMR spectra of compound **23**

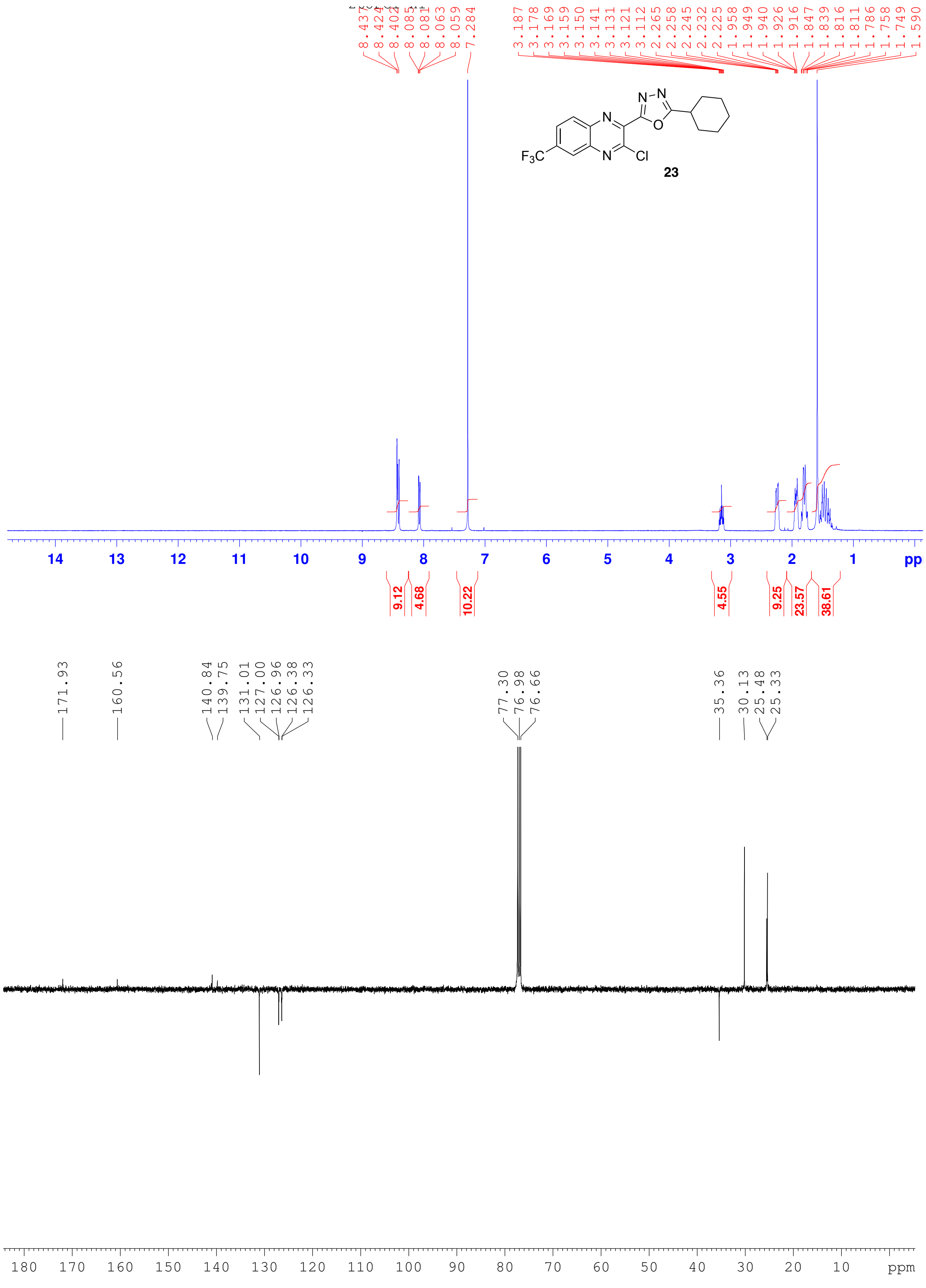

Figure S27. ^{1}H- and ^{13}C-NMR spectra of compound **24**

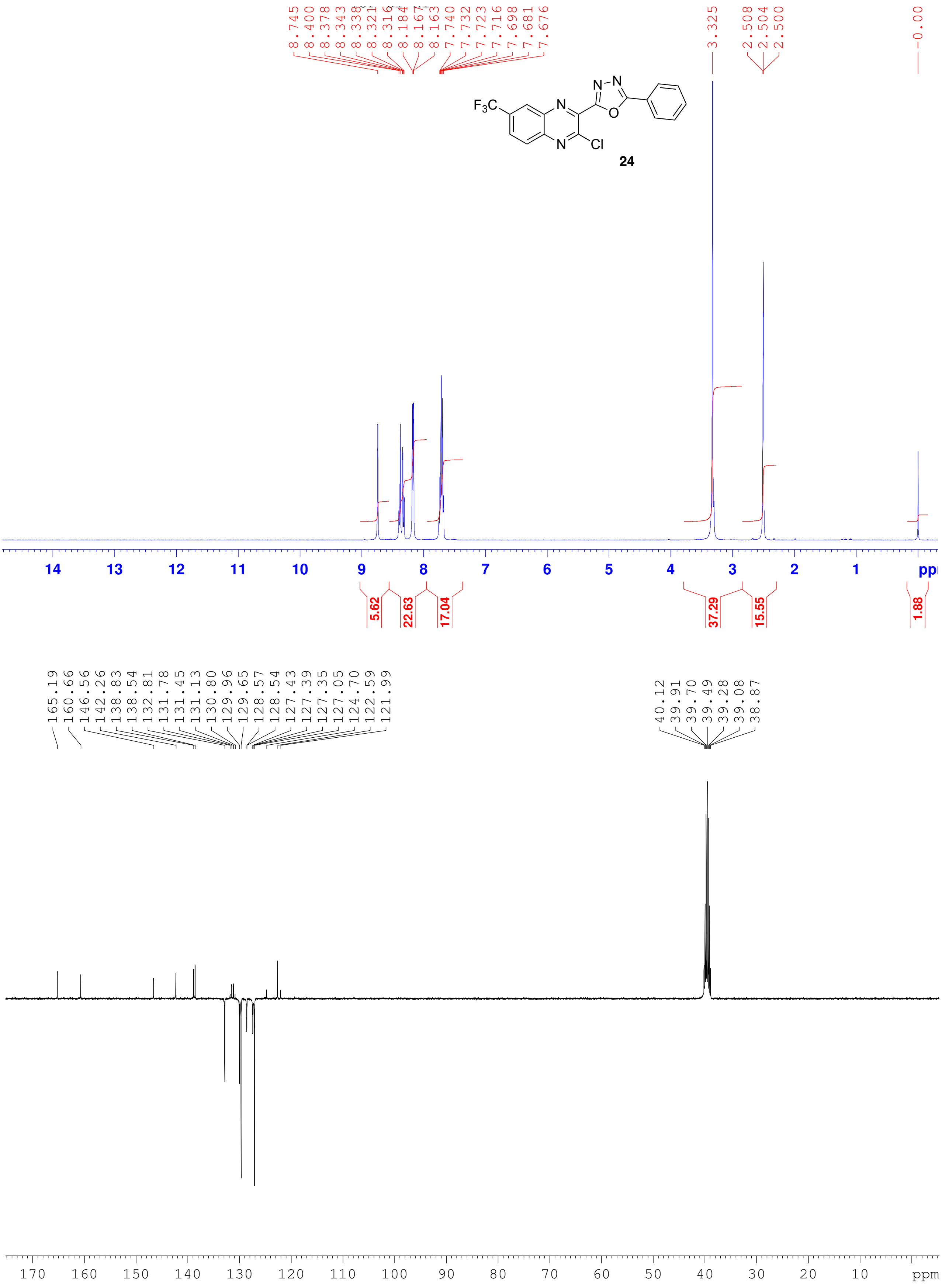

Figure S28. ^{1}H- and ^{13}C-NMR spectra of compound **25**

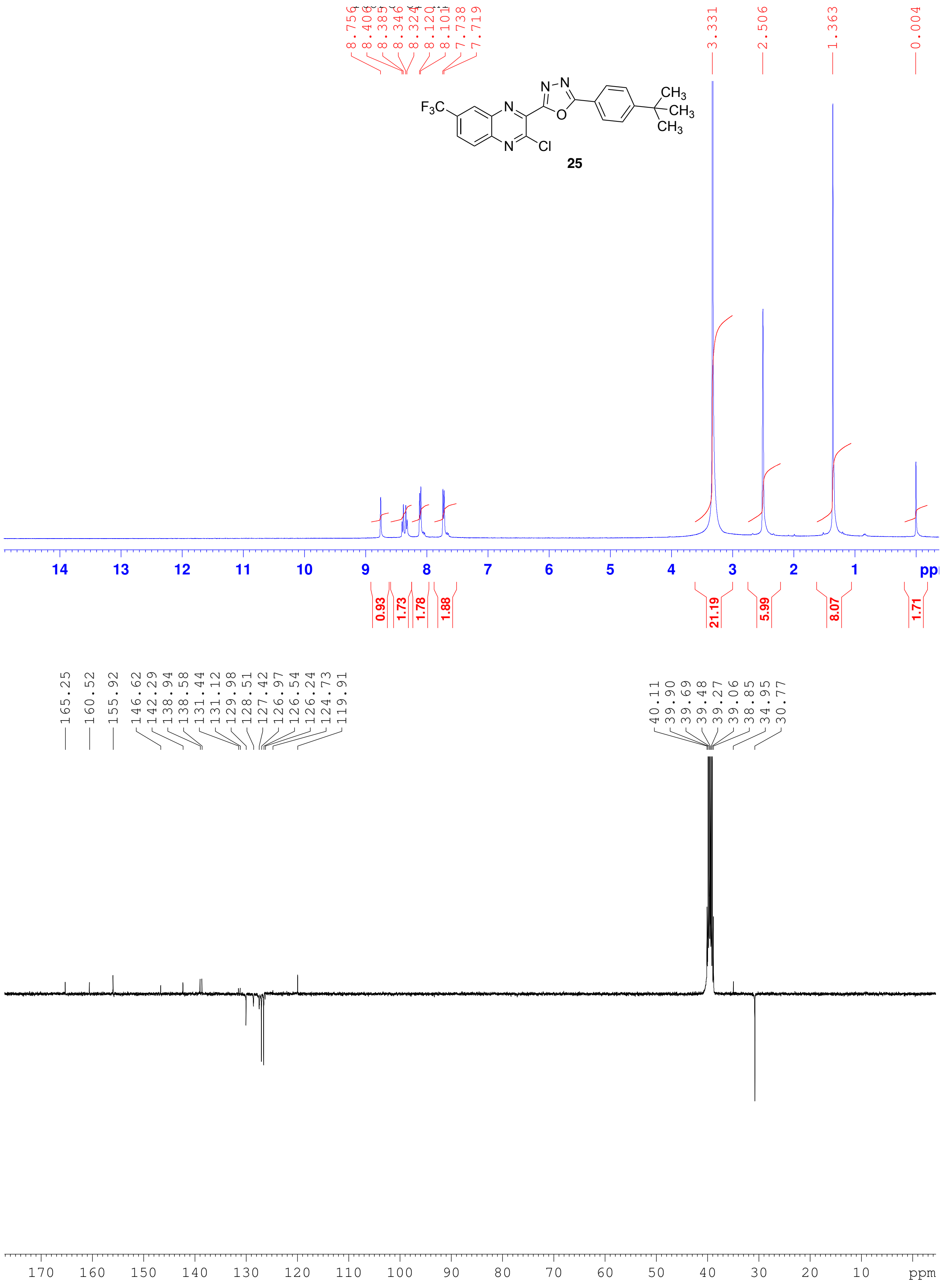

Figure S29. ^{1}H- and ^{13}C-NMR spectra of compound **26**

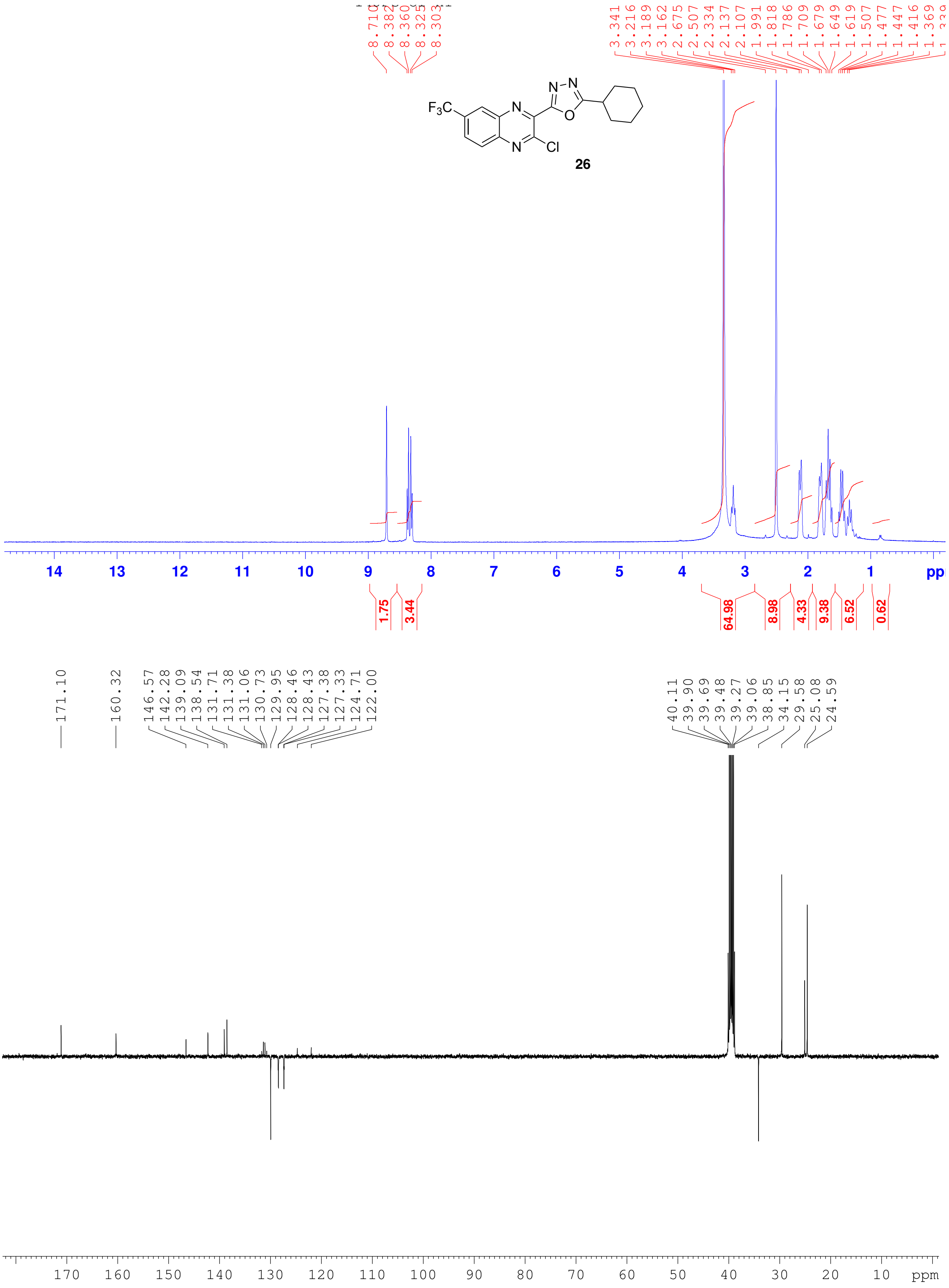

Figure S30. ^{1}H- and ^{13}C-NMR spectra of compound **27**

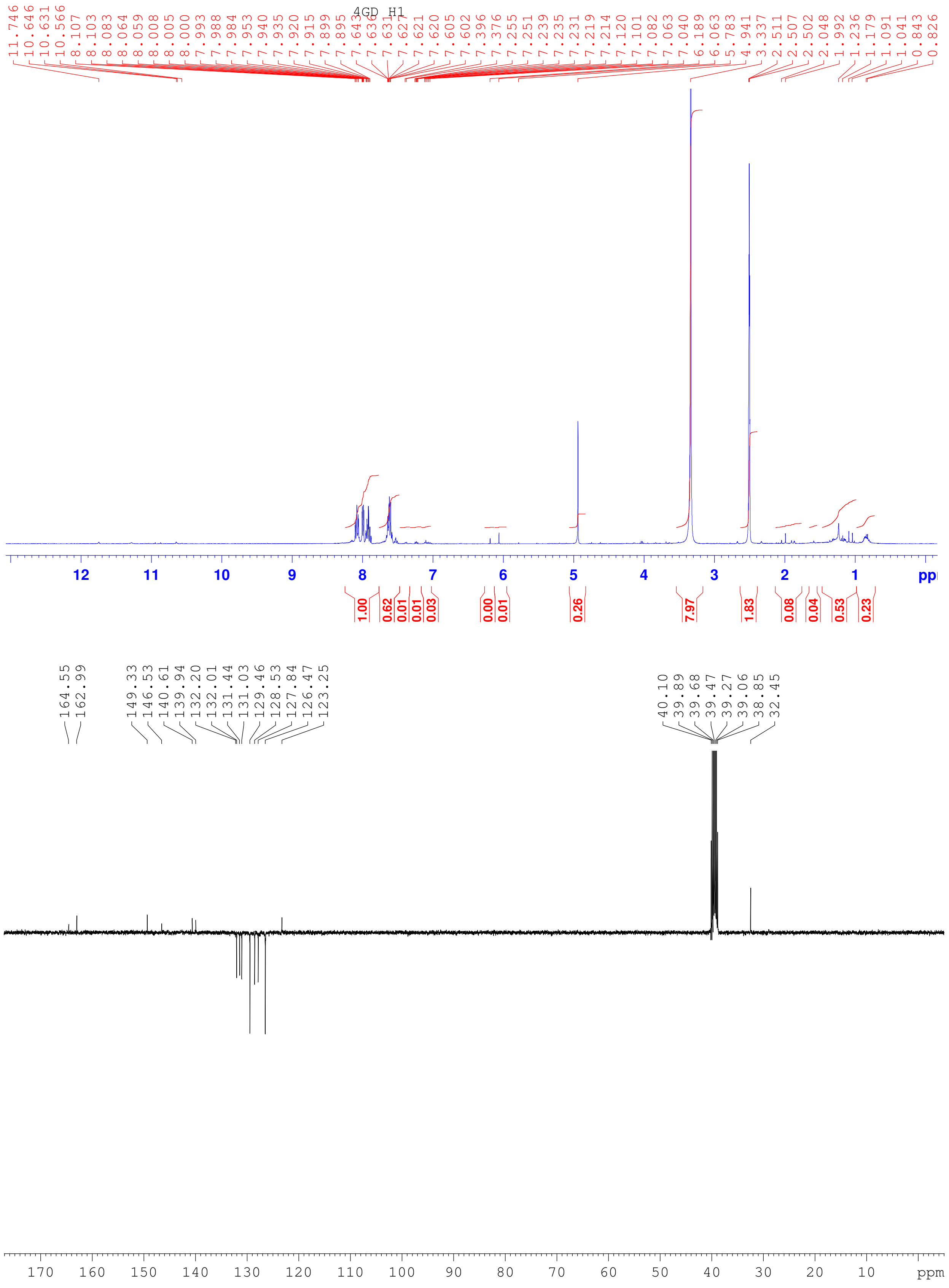

Figure S31. ^{1}H- and ^{13}C-NMR spectra of compound **28**

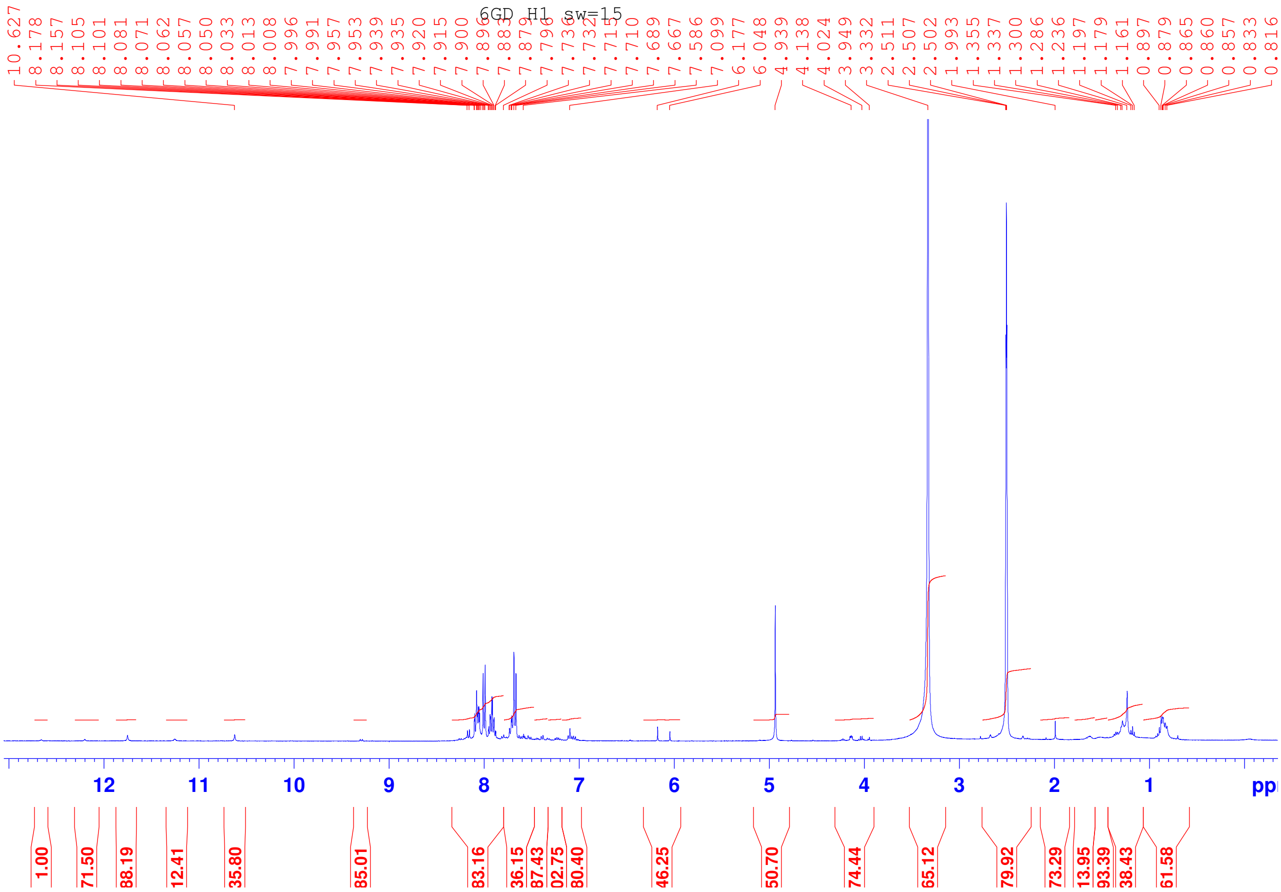

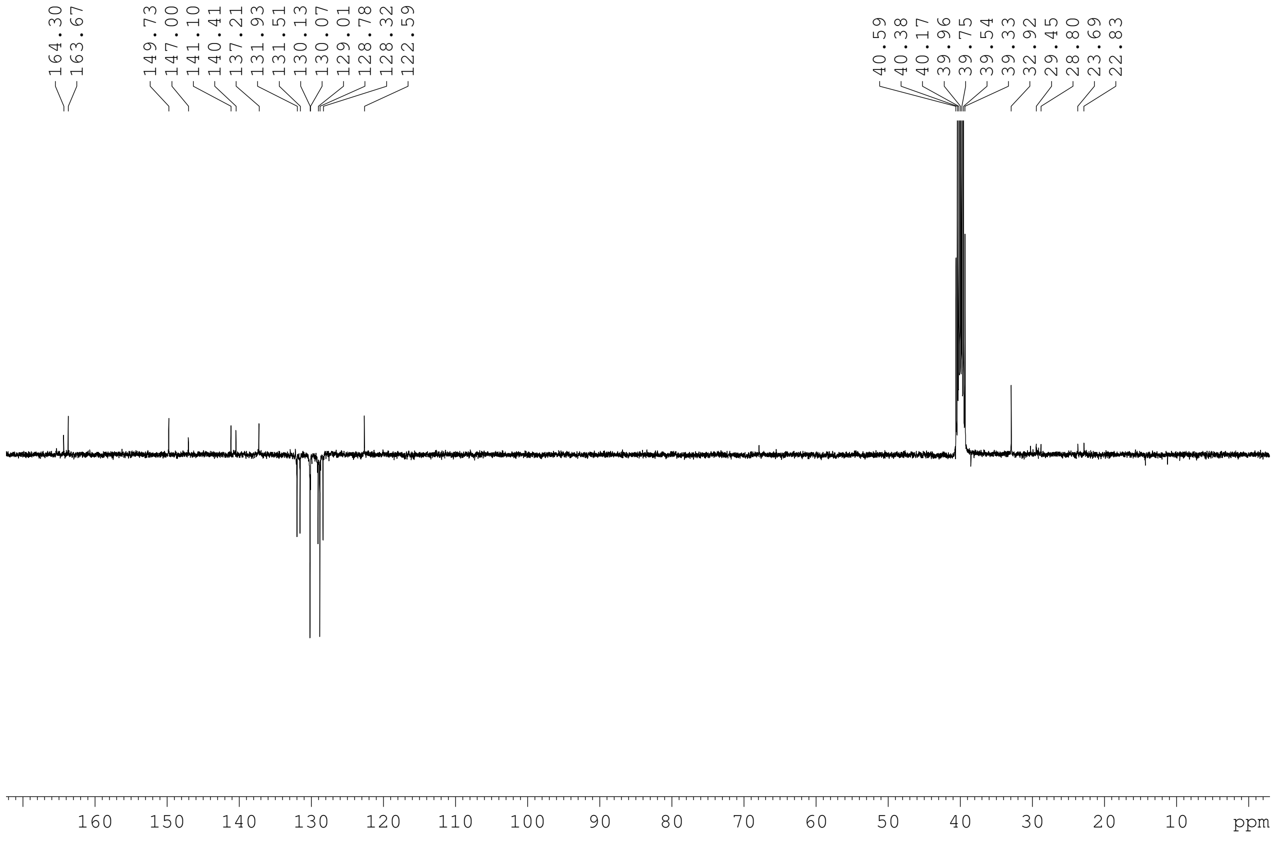

Figure S32. One dose assay of compound **4** (NSC 823912).

Developmental Therapeutics Program
One Dose Mean Graph

NSC: D-823912 / 1 | **Conc:** 1.00E-5 Molar | **Test Date:** Mar 23, 2020

Experiment ID: 2003OS27 | **Report Date:** Jun 17, 2020

Panel/Cell Line	Growth Percent
**Leukemia**	
CCRF-CEM	92.12
HL-60(TB)	96.02
K-562	92.69
MOLT-4	104.30
RPMI-8226	89.74
SR	85.62
**Non-Small Cell Lung Cancer**	
A549/ATCC	103.58
EKVX	98.99
HOP-62	108.01
HOP-92	98.32
NCI-H226	103.09
NCI-H23	99.78
NCI-H322M	103.13
NCI-H460	110.53
NCI-H522	95.37
**Colon Cancer**	
COLO 205	121.47
HCC-2998	103.96
HCT-116	94.06
HCT-15	96.74
HT29	120.85
KM12	97.68
SW-620	104.44
**CNS Cancer**	
SF-268	99.39
SF-295	98.63
SF-539	96.07
SNB-19	94.12
SNB-75	88.64
U251	99.27
**Melanoma**	
MALME-3M	94.88
M14	97.68
MDA-MB-435	103.04
SK-MEL-2	111.99
SK-MEL-28	106.75
SK-MEL-5	100.08
UACC-257	108.28
UACC-62	94.21
**Ovarian Cancer**	
IGROV1	94.99
OVCAR-3	112.50
OVCAR-4	96.89
OVCAR-5	100.23
OVCAR-8	99.56
NCI/ADR-RES	99.60
SK-OV-3	112.80
**Renal Cancer**	
786-0	100.56
A498	109.66
ACHN	87.43
CAKI-1	82.53
RXF 393	116.84
SN12C	97.56
TK-10	129.48
UO-31	102.38
**Prostate Cancer**	
PC-3	91.17
DU-145	106.99
**Breast Cancer**	
MCF7	72.76
MDA-MB-231/ATCC	99.03
HS 578T	97.88
BT-549	98.58
T-47D	89.79
MDA-MB-468	76.38
Mean	99.82
Delta	27.06
Range	56.72

Mean Growth Percent - Growth Percent

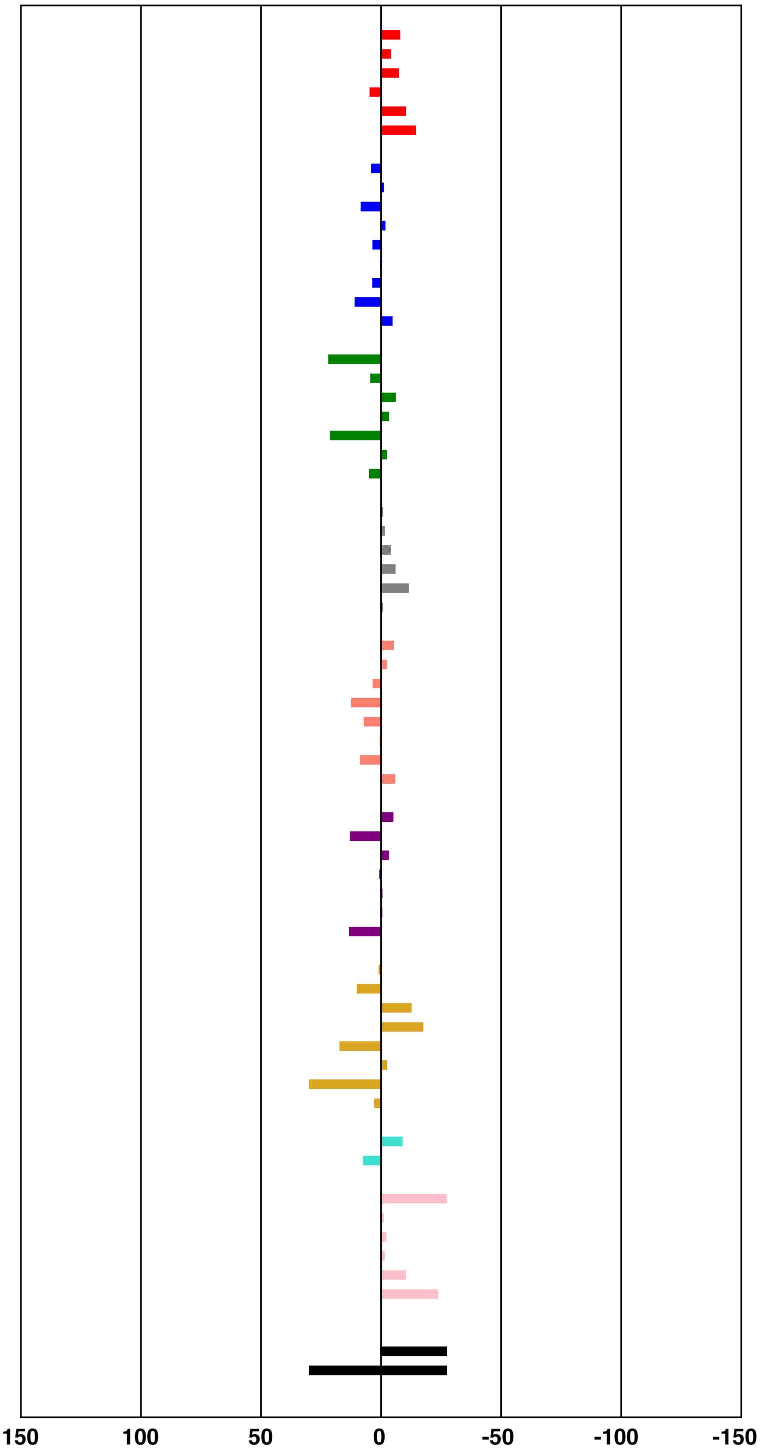

Figure S33. One dose assay of compound **5** (NSC 823919).

Developmental Therapeutics Program One Dose Mean Graph	NSC: D-823919 / 1	Conc: 1.00E-5 Molar	Test Date: Mar 23, 2020
	Experiment ID: 2003OS27		Report Date: Jun 17, 2020

Panel/Cell Line	Growth Percent
Leukemia	
CCRF-CEM	103.86
HL-60(TB)	100.70
K-562	99.74
MOLT-4	106.84
RPMI-8226	98.00
SR	106.36
Non-Small Cell Lung Cancer	
A549/ATCC	112.21
EKVX	106.79
HOP-62	103.77
HOP-92	104.22
NCI-H226	101.39
NCI-H23	107.16
NCI-H322M	108.38
NCI-H460	115.91
NCI-H522	92.99
Colon Cancer	
COLO 205	117.89
HCC-2998	98.00
HCT-116	104.14
HCT-15	111.22
HT29	112.82
KM12	101.71
SW-620	110.35
CNS Cancer	
SF-268	97.80
SF-295	110.59
SF-539	101.37
SNB-19	99.09
SNB-75	96.07
U251	104.29
Melanoma	
MALME-3M	99.27
M14	103.57
MDA-MB-435	101.14
SK-MEL-2	111.49
SK-MEL-28	112.03
SK-MEL-5	98.30
UACC-257	111.49
UACC-62	98.10
Ovarian Cancer	
IGROV1	95.33
OVCAR-3	110.65
OVCAR-4	100.90
OVCAR-5	105.02
OVCAR-8	104.19
NCI/ADR-RES	108.28
SK-OV-3	108.43
Renal Cancer	
786-0	107.31
A498	120.96
ACHN	104.76
CAKI-1	86.04
RXF 393	117.45
SN12C	97.61
TK-10	131.29
UO-31	78.01
Prostate Cancer	
PC-3	100.23
DU-145	114.41
Breast Cancer	
MCF7	96.72
MDA-MB-231/ATCC	96.51
HS 578T	115.97
BT-549	108.88
T-47D	84.72
MDA-MB-468	83.96
Mean	104.18
Delta	26.17
Range	53.28

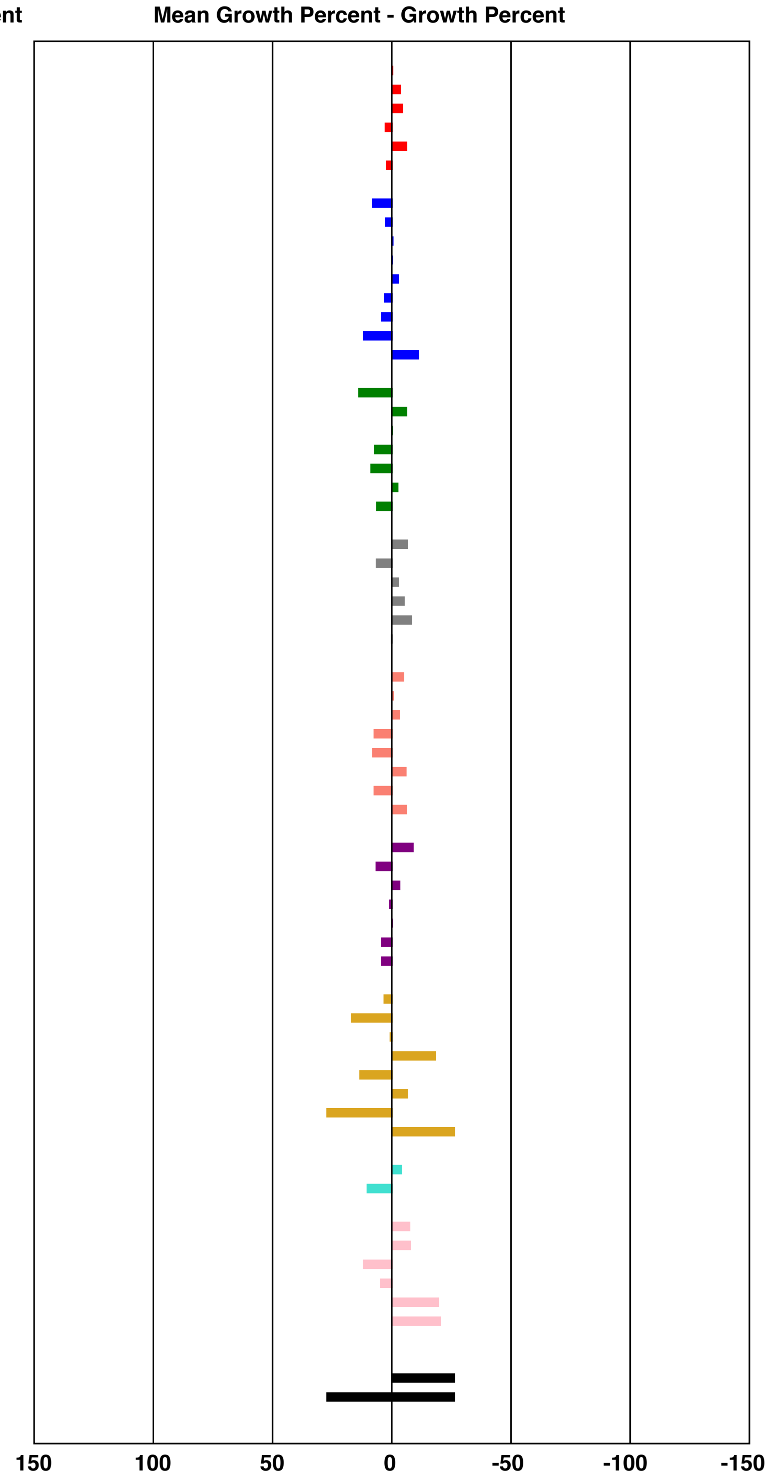

Figure S34. One dose assay of compound **6** (NSC 823911).

Developmental Therapeutics Program One Dose Mean Graph	NSC: D-823911 / 1	Conc: 1.00E-5 Molar	Test Date: Mar 23, 2020
	Experiment ID: 2003OS27		Report Date: Jun 17, 2020

Panel/Cell Line	Growth Percent
**Leukemia**	
CCRF-CEM	98.49
HL-60(TB)	96.36
K-562	100.68
MOLT-4	98.96
RPMI-8226	91.76
SR	108.11
**Non-Small Cell Lung Cancer**	
A549/ATCC	118.16
EKVX	104.08
HOP-62	103.35
HOP-92	99.35
NCI-H226	93.81
NCI-H23	103.19
NCI-H322M	100.88
NCI-H460	117.87
NCI-H522	98.82
**Colon Cancer**	
COLO 205	127.72
HCC-2998	108.73
HCT-116	102.99
HCT-15	104.19
HT29	117.78
KM12	101.82
SW-620	108.88
**CNS Cancer**	
SF-268	96.52
SF-295	112.14
SF-539	104.29
SNB-19	101.97
SNB-75	94.71
U251	103.76
**Melanoma**	
MALME-3M	94.23
M14	101.61
MDA-MB-435	105.54
SK-MEL-2	114.61
SK-MEL-28	110.31
SK-MEL-5	98.74
UACC-257	109.72
UACC-62	97.78
**Ovarian Cancer**	
IGROV1	90.16
OVCAR-3	99.68
OVCAR-4	108.89
OVCAR-5	108.18
OVCAR-8	105.48
NCI/ADR-RES	104.35
SK-OV-3	102.15
**Renal Cancer**	
786-0	109.15
A498	121.16
ACHN	103.33
CAKI-1	87.95
RXF 393	112.01
SN12C	101.90
TK-10	144.29
UO-31	73.36
**Prostate Cancer**	
PC-3	101.57
DU-145	106.31
**Breast Cancer**	
MCF7	76.19
MDA-MB-231/ATCC	100.22
HS 578T	111.23
BT-549	111.30
T-47D	81.02
MDA-MB-468	86.82
Mean	103.37
Delta	30.01
Range	70.93

Mean Growth Percent - Growth Percent

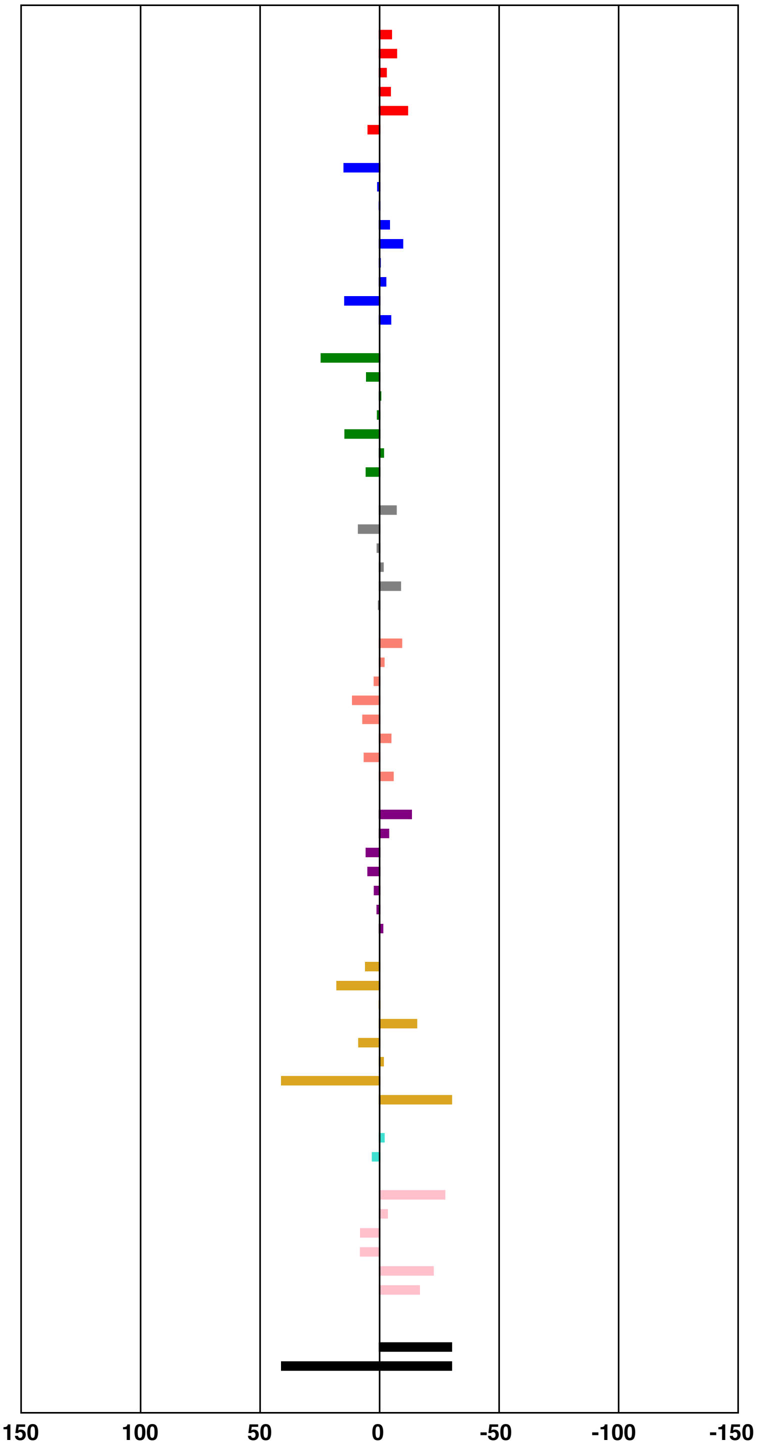

Figure S35. One dose assay of compound **7** (NSC 823915).

Developmental Therapeutics Program One Dose Mean Graph	NSC: D-823915 / 1	Conc: 1.00E-5 Molar	Test Date: Mar 23, 2020
	Experiment ID: 2003OS27		Report Date: Jun 17, 2020

Panel/Cell Line	Growth Percent
**Leukemia**	
CCRF-CEM	98.51
HL-60(TB)	98.83
K-562	104.16
MOLT-4	102.22
RPMI-8226	100.58
SR	112.35
**Non-Small Cell Lung Cancer**	
A549/ATCC	107.66
EKVX	104.52
HOP-62	102.96
HOP-92	98.32
NCI-H226	102.32
NCI-H23	105.47
NCI-H322M	100.94
NCI-H460	107.16
NCI-H522	96.60
**Colon Cancer**	
COLO 205	116.18
HCC-2998	108.44
HCT-116	96.91
HCT-15	105.55
HT29	115.24
KM12	104.41
SW-620	105.46
**CNS Cancer**	
SF-268	97.26
SF-295	104.03
SF-539	107.61
SNB-19	99.52
SNB-75	94.51
U251	104.96
**Melanoma**	
MALME-3M	103.81
M14	104.48
MDA-MB-435	103.82
SK-MEL-2	106.97
SK-MEL-28	104.71
SK-MEL-5	101.24
UACC-257	111.30
UACC-62	95.71
**Ovarian Cancer**	
IGROV1	105.03
OVCAR-3	117.37
OVCAR-4	113.75
OVCAR-5	102.51
OVCAR-8	106.82
NCI/ADR-RES	109.31
SK-OV-3	117.25
**Renal Cancer**	
786-0	102.90
A498	113.74
ACHN	95.27
CAKI-1	94.73
RXF 393	109.72
SN12C	102.71
TK-10	111.71
UO-31	81.94
**Prostate Cancer**	
PC-3	98.26
DU-145	110.38
**Breast Cancer**	
MCF7	85.31
MDA-MB-231/ATCC	111.33
HS 578T	105.73
BT-549	100.38
T-47D	109.90
MDA-MB-468	99.92
Mean	104.08
Delta	22.14
Range	35.43

Mean Growth Percent - Growth Percent

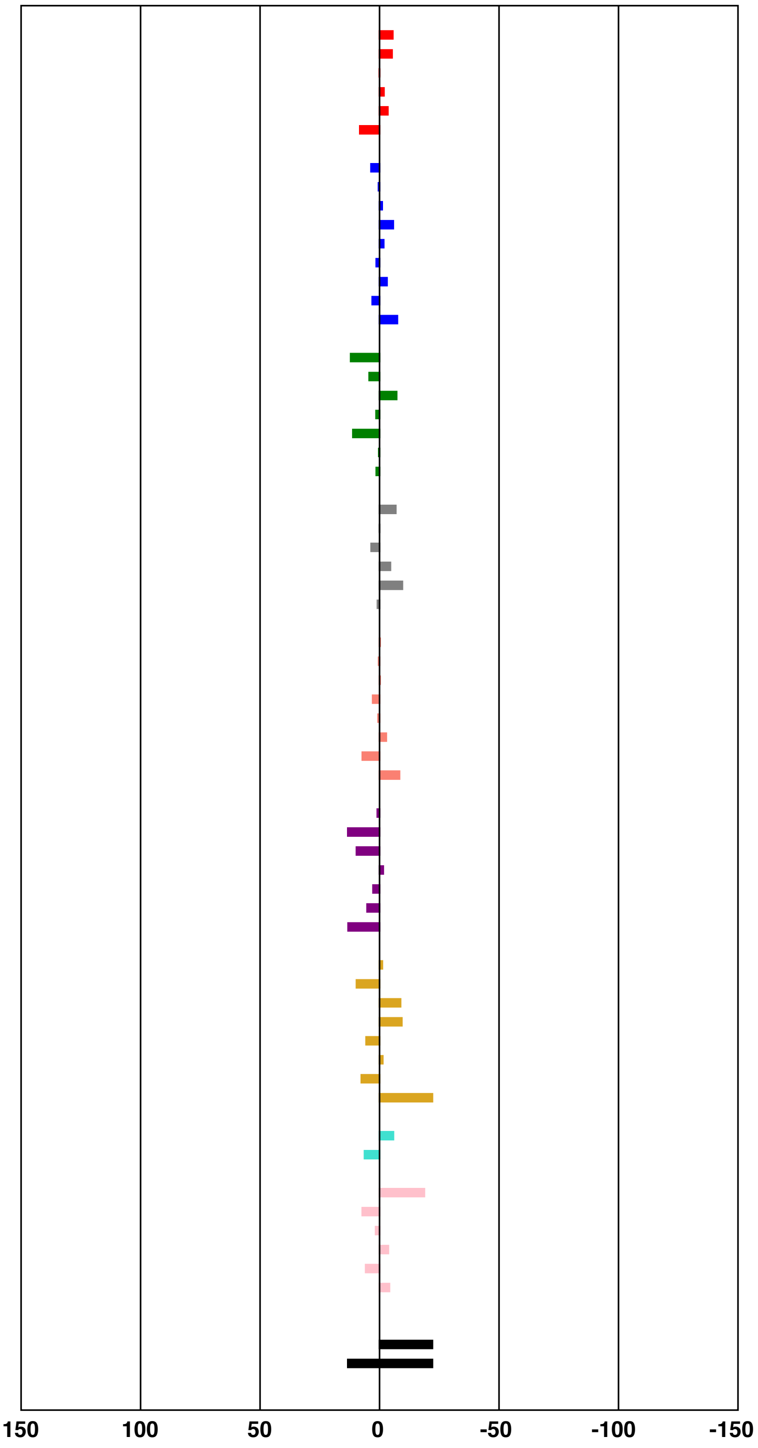

Figure S36. One dose assay of compound **8** (NSC 830863).

Developmental Therapeutics Program
One Dose Mean Graph

NSC: D-830863 / 1 | **Conc:** 1.00E-5 Molar | **Test Date:** Jun 14, 2021

Experiment ID: 2106OS47 | **Report Date:** Sep 07, 2021

Panel/Cell Line	Growth Percent
Leukemia	
CCRF-CEM	103.04
HL-60(TB)	13.57
K-562	65.86
MOLT-4	101.43
RPMI-8226	65.90
SR	91.27
Non-Small Cell Lung Cancer	
A549/ATCC	102.07
EKVX	99.24
HOP-62	11.11
NCI-H226	86.54
NCI-H23	90.85
NCI-H322M	92.93
NCI-H460	88.10
NCI-H522	99.10
Colon Cancer	
COLO 205	104.37
HCC-2998	98.53
HCT-116	14.17
HCT-15	81.86
HT29	116.13
KM12	65.79
SW-620	66.24
CNS Cancer	
SF-268	41.98
SF-295	103.06
SF-539	93.84
SNB-19	95.83
SNB-75	96.38
U251	98.20
Melanoma	
LOX IMVI	89.98
MALME-3M	101.46
M14	93.08
MDA-MB-435	68.95
SK-MEL-2	63.83
SK-MEL-28	68.44
SK-MEL-5	98.13
UACC-257	61.93
UACC-62	72.73
Ovarian Cancer	
IGROV1	72.51
OVCAR-4	82.21
OVCAR-5	99.10
OVCAR-8	84.21
NCI/ADR-RES	82.50
SK-OV-3	95.90
Renal Cancer	
786-0	105.01
A498	89.39
ACHN	102.32
CAKI-1	89.17
RXF 393	88.39
SN12C	86.21
TK-10	107.45
UO-31	79.45
Prostate Cancer	
PC-3	92.44
DU-145	86.17
Breast Cancer	
MCF7	-65.85
MDA-MB-231/ATCC	90.45
HS 578T	-8.86
BT-549	103.93
T-47D	-70.52
MDA-MB-468	7.12
Mean	75.94
Delta	146.46
Range	186.65

Mean Growth Percent - Growth Percent

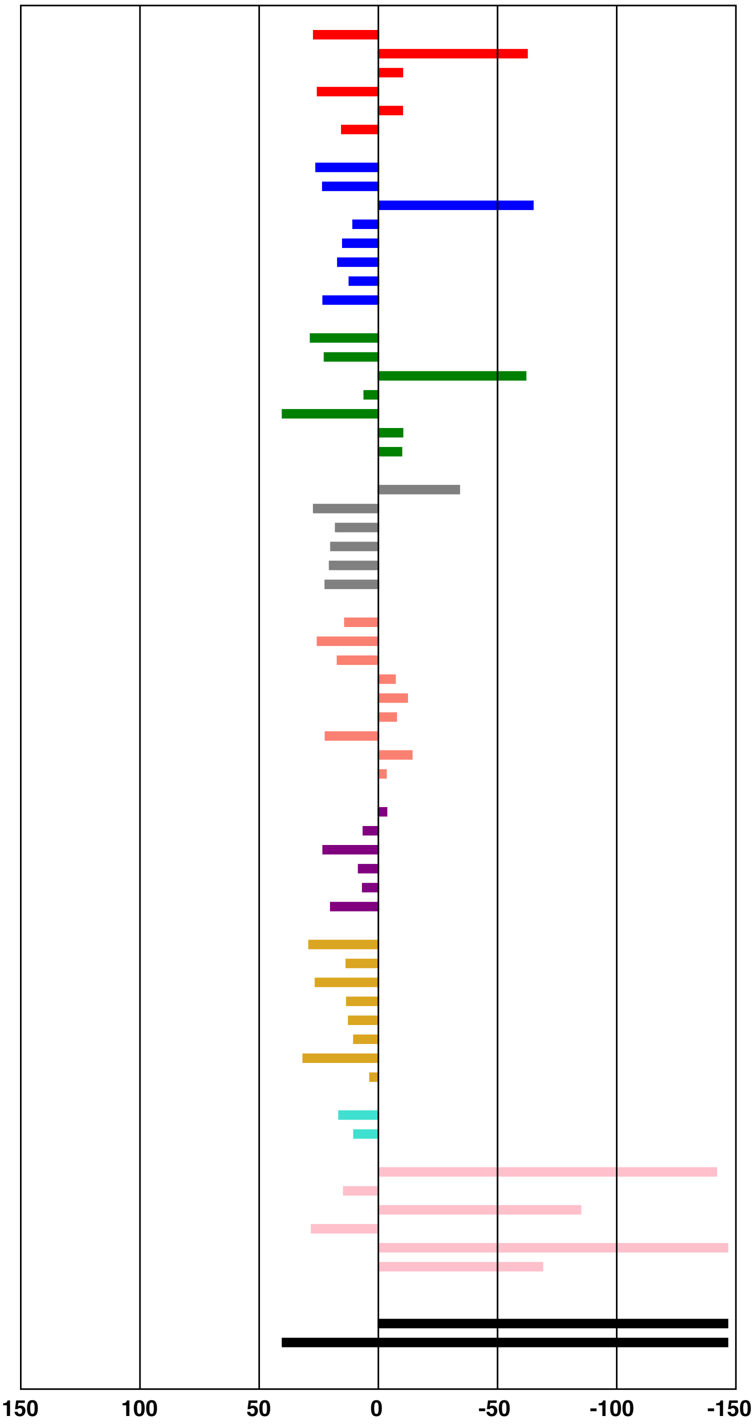

Figure S37. One dose assay of compound **9** (NSC 830867).

Developmental Therapeutics Program One Dose Mean Graph	NSC: D-830867 / 1	Conc: 1.00E-5 Molar	Test Date: Jun 14, 2021
	Experiment ID: 2106OS47		Report Date: Sep 07, 2021

Panel/Cell Line	Growth Percent
Leukemia	
CCRF-CEM	84.87
HL-60(TB)	66.56
K-562	80.87
MOLT-4	108.54
RPMI-8226	95.39
Non-Small Cell Lung Cancer	
A549/ATCC	98.39
EKVX	98.82
HOP-62	106.13
NCI-H226	84.73
NCI-H23	86.28
NCI-H322M	97.75
NCI-H460	97.48
NCI-H522	99.05
Colon Cancer	
COLO 205	109.86
HCC-2998	103.86
HCT-116	101.32
HCT-15	71.74
HT29	109.11
KM12	94.10
SW-620	97.21
CNS Cancer	
SF-268	100.66
SF-295	106.29
SF-539	97.72
SNB-19	98.69
SNB-75	97.24
U251	97.91
Melanoma	
LOX IMVI	66.63
MALME-3M	95.23
M14	94.33
MDA-MB-435	99.10
SK-MEL-2	102.29
SK-MEL-28	104.04
SK-MEL-5	98.79
UACC-257	96.65
UACC-62	96.39
Ovarian Cancer	
IGROV1	101.24
OVCAR-4	97.98
OVCAR-5	106.75
OVCAR-8	101.26
NCI/ADR-RES	107.36
SK-OV-3	101.81
Renal Cancer	
786-0	105.70
A498	89.32
ACHN	99.24
CAKI-1	89.49
RXF 393	87.58
SN12C	101.19
TK-10	105.46
UO-31	92.66
Prostate Cancer	
PC-3	95.80
DU-145	91.31
Breast Cancer	
MCF7	37.39
MDA-MB-231/ATCC	104.65
HS 578T	92.81
BT-549	137.68
T-47D	86.00
MDA-MB-468	7.71
Mean	94.46
Delta	86.75
Range	129.97

Mean Growth Percent - Growth Percent

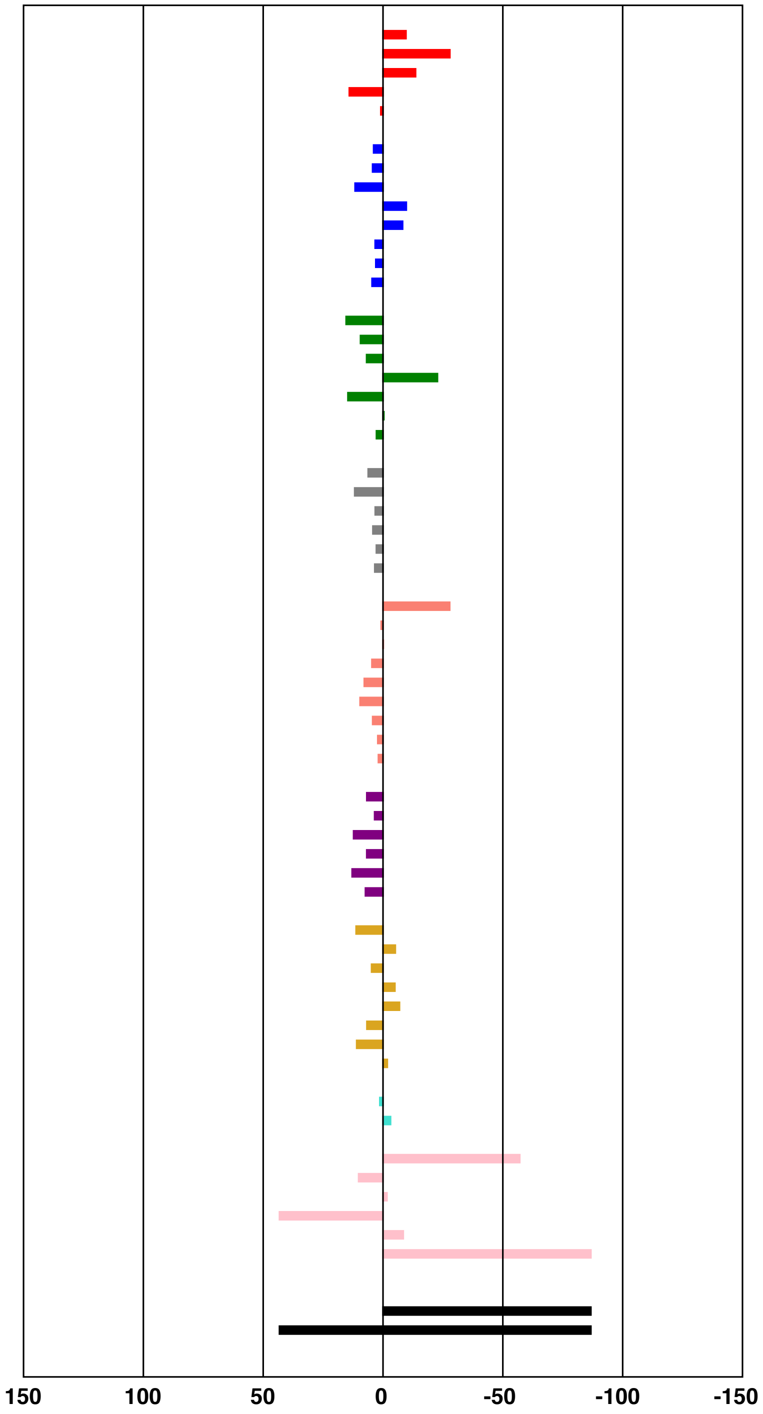

Figure S38. One dose assay of compound **10** (NSC 830864).

Developmental Therapeutics Program One Dose Mean Graph	NSC: D-830864 / 1	Conc: 1.00E-5 Molar	Test Date: Jun 14, 2021
	Experiment ID: 2106OS47		Report Date: Sep 07, 2021

Panel/Cell Line	Growth Percent
Leukemia	
CCRF-CEM	97.10
HL-60(TB)	82.73
K-562	98.16
MOLT-4	104.86
RPMI-8226	100.63
SR	82.46
Non-Small Cell Lung Cancer	
A549/ATCC	100.82
EKVX	100.89
HOP-62	99.06
NCI-H226	96.08
NCI-H23	94.20
NCI-H322M	102.55
NCI-H460	99.16
NCI-H522	100.58
Colon Cancer	
COLO 205	106.19
HCC-2998	103.68
HCT-116	102.65
HCT-15	90.64
HT29	112.47
KM12	85.13
SW-620	103.67
CNS Cancer	
SF-268	103.19
SF-295	108.98
SF-539	97.26
SNB-19	98.72
SNB-75	93.54
U251	102.31
Melanoma	
LOX IMVI	87.95
MALME-3M	98.47
M14	98.30
MDA-MB-435	98.71
SK-MEL-2	102.63
SK-MEL-28	102.48
SK-MEL-5	99.25
UACC-257	97.83
UACC-62	94.14
Ovarian Cancer	
IGROV1	100.42
OVCAR-4	92.86
OVCAR-5	110.25
OVCAR-8	99.78
NCI/ADR-RES	105.25
SK-OV-3	92.70
Renal Cancer	
786-0	106.28
A498	92.85
ACHN	95.31
CAKI-1	84.40
RXF 393	100.06
SN12C	98.55
TK-10	132.73
UO-31	81.78
Prostate Cancer	
PC-3	90.41
DU-145	47.45
Breast Cancer	
MCF7	46.95
MDA-MB-231/ATCC	101.74
HS 578T	90.55
BT-549	128.49
T-47D	57.55
MDA-MB-468	17.74
Mean	95.20
Delta	77.46
Range	114.99

Mean Growth Percent - Growth Percent

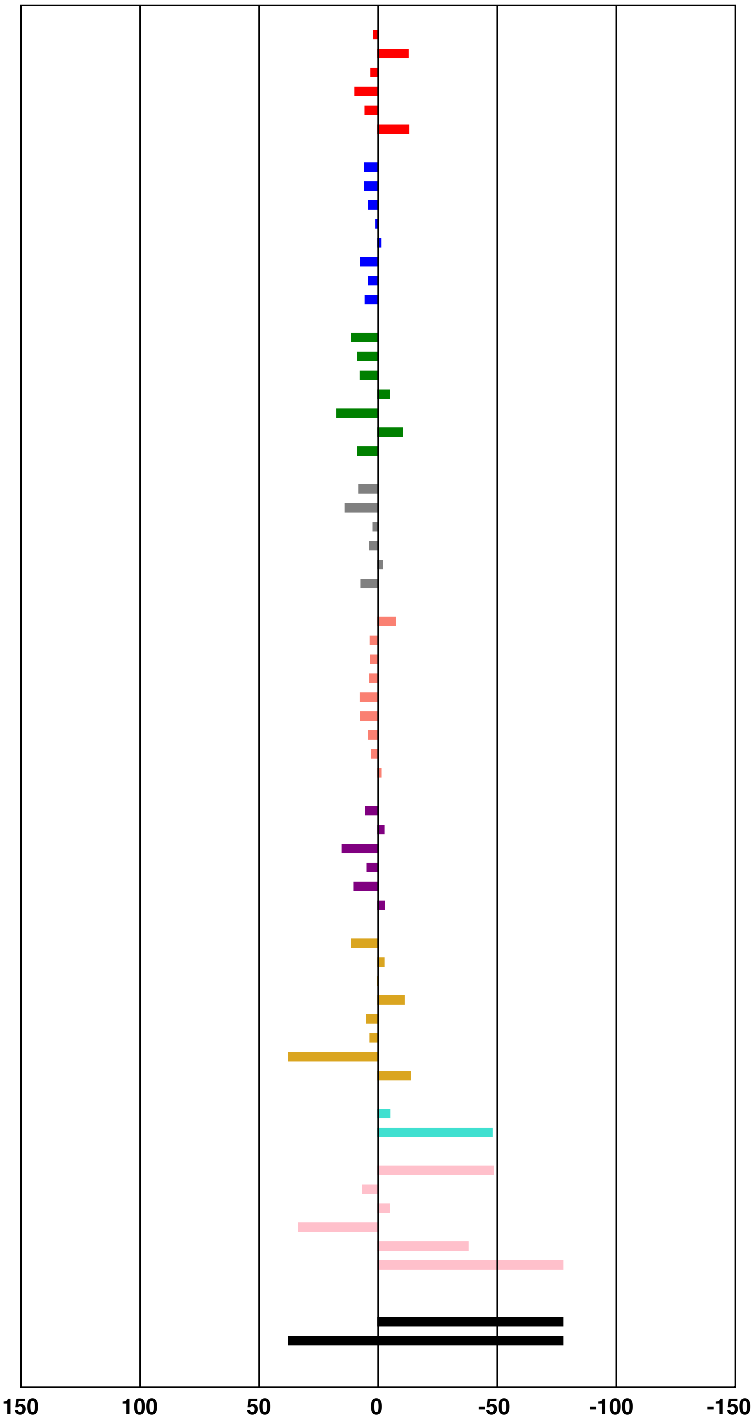

Figure S39. One dose assay of compound **11** (NSC 830865).

**Developmental Therapeutics Program One Dose Mean Graph**	**NSC:** D-830865 / 1	**Conc:** 1.00E-5 Molar	**Test Date:** Jun 14, 2021
	**Experiment ID:** 2106OS47		**Report Date:** Sep 07, 2021

Panel/Cell Line	Growth Percent
Leukemia	
CCRF-CEM	104.88
HL-60(TB)	97.10
K-562	99.93
MOLT-4	104.26
RPMI-8226	99.71
SR	95.21
Non-Small Cell Lung Cancer	
A549/ATCC	98.21
EKVX	101.49
HOP-62	109.89
NCI-H226	95.12
NCI-H23	99.05
NCI-H322M	100.26
NCI-H460	101.99
NCI-H522	99.82
Colon Cancer	
COLO 205	106.56
HCC-2998	111.90
HCT-116	103.71
HCT-15	93.94
HT29	111.24
KM12	101.82
SW-620	98.96
CNS Cancer	
SF-268	102.87
SF-295	93.32
SF-539	101.80
SNB-19	96.58
SNB-75	88.89
U251	104.13
Melanoma	
LOX IMVI	96.21
MALME-3M	100.86
M14	101.19
MDA-MB-435	100.46
SK-MEL-2	105.89
SK-MEL-28	110.93
SK-MEL-5	99.00
UACC-257	102.51
UACC-62	95.77
Ovarian Cancer	
IGROV1	96.46
OVCAR-4	96.79
OVCAR-5	107.51
OVCAR-8	100.39
NCI/ADR-RES	101.06
SK-OV-3	94.26
Renal Cancer	
786-0	104.25
A498	89.53
ACHN	103.24
CAKI-1	89.36
RXF 393	101.96
SN12C	100.01
TK-10	126.62
UO-31	85.58
Prostate Cancer	
PC-3	95.42
DU-145	103.14
Breast Cancer	
MCF7	50.16
MDA-MB-231/ATCC	104.95
HS 578T	97.20
BT-549	136.40
T-47D	79.54
MDA-MB-468	74.82
Mean	99.55
Delta	49.39
Range	86.24

Mean Growth Percent - Growth Percent

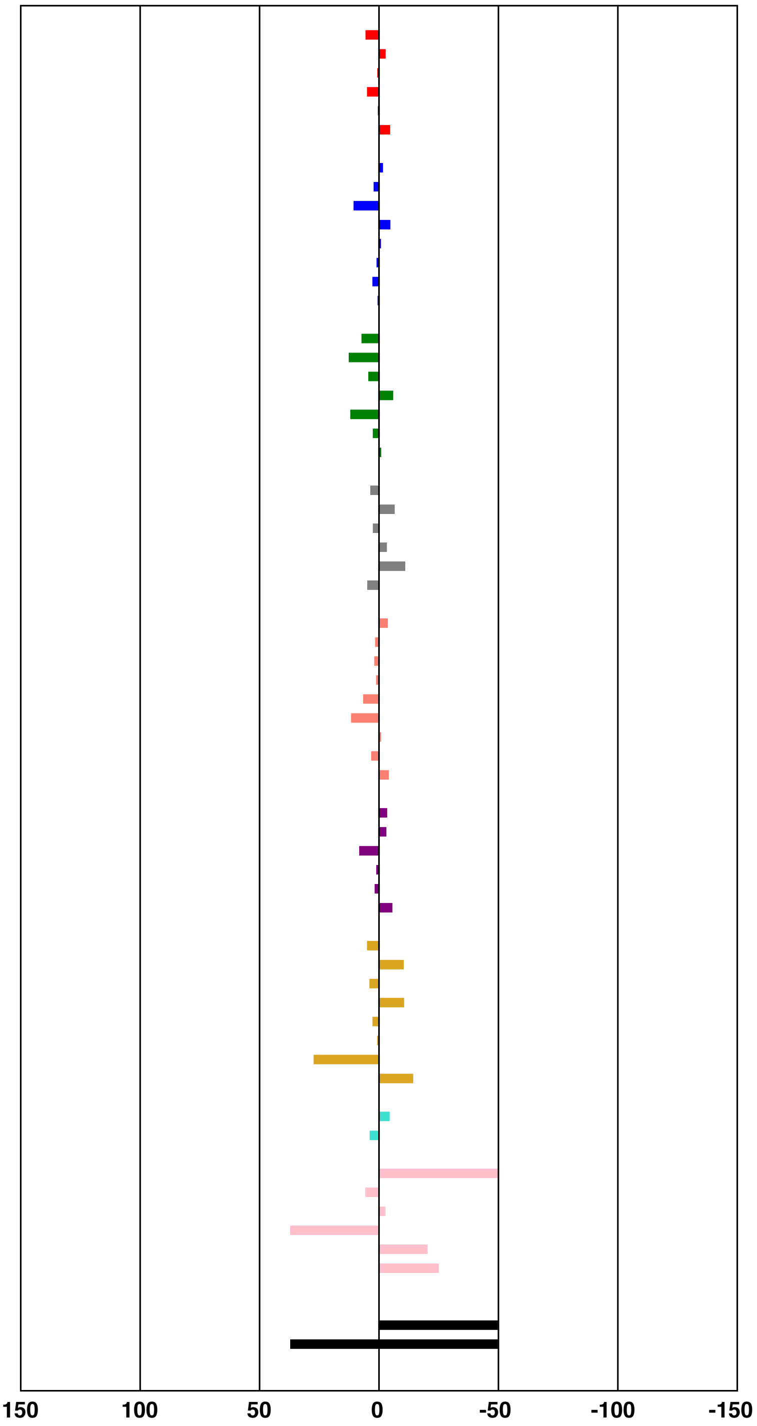

Figure S40. One dose assay of compound **12** (NSC 830866).

Developmental Therapeutics Program One Dose Mean Graph	**NSC:** D-830866 / 1	**Conc:** 1.00E-5 Molar	**Test Date:** Jun 14, 2021
	**Experiment ID:** 2106OS47		**Report Date:** Sep 07, 2021

Panel/Cell Line	Growth Percent
Leukemia	
CCRF-CEM	94.18
HL-60(TB)	91.66
K-562	93.09
MOLT-4	100.56
RPMI-8226	96.00
SR	74.91
Non-Small Cell Lung Cancer	
A549/ATCC	97.20
EKVX	100.98
HOP-62	100.82
NCI-H226	91.67
NCI-H23	95.76
NCI-H322M	102.08
NCI-H460	96.45
NCI-H522	98.99
Colon Cancer	
COLO 205	107.72
HCC-2998	108.42
HCT-116	91.64
HCT-15	81.90
HT29	107.36
KM12	95.42
SW-620	92.49
CNS Cancer	
SF-268	95.22
SF-295	103.93
SF-539	94.80
SNB-19	97.06
SNB-75	93.75
U251	98.10
Melanoma	
LOX IMVI	81.59
MALME-3M	80.38
M14	87.69
MDA-MB-435	96.85
SK-MEL-2	100.94
SK-MEL-28	109.49
SK-MEL-5	99.24
UACC-257	98.29
UACC-62	96.35
Ovarian Cancer	
IGROV1	97.81
OVCAR-4	96.13
OVCAR-5	101.40
OVCAR-8	98.51
NCI/ADR-RES	98.53
SK-OV-3	93.59
Renal Cancer	
786-0	104.61
A498	95.46
ACHN	97.29
CAKI-1	85.44
RXF 393	95.25
SN12C	105.04
TK-10	108.80
UO-31	80.96
Prostate Cancer	
PC-3	91.05
DU-145	99.51
Breast Cancer	
MCF7	65.35
MDA-MB-231/ATCC	105.06
HS 578T	86.31
BT-549	114.17
T-47D	87.89
MDA-MB-468	77.11
Mean	95.49
Delta	30.14
Range	48.82

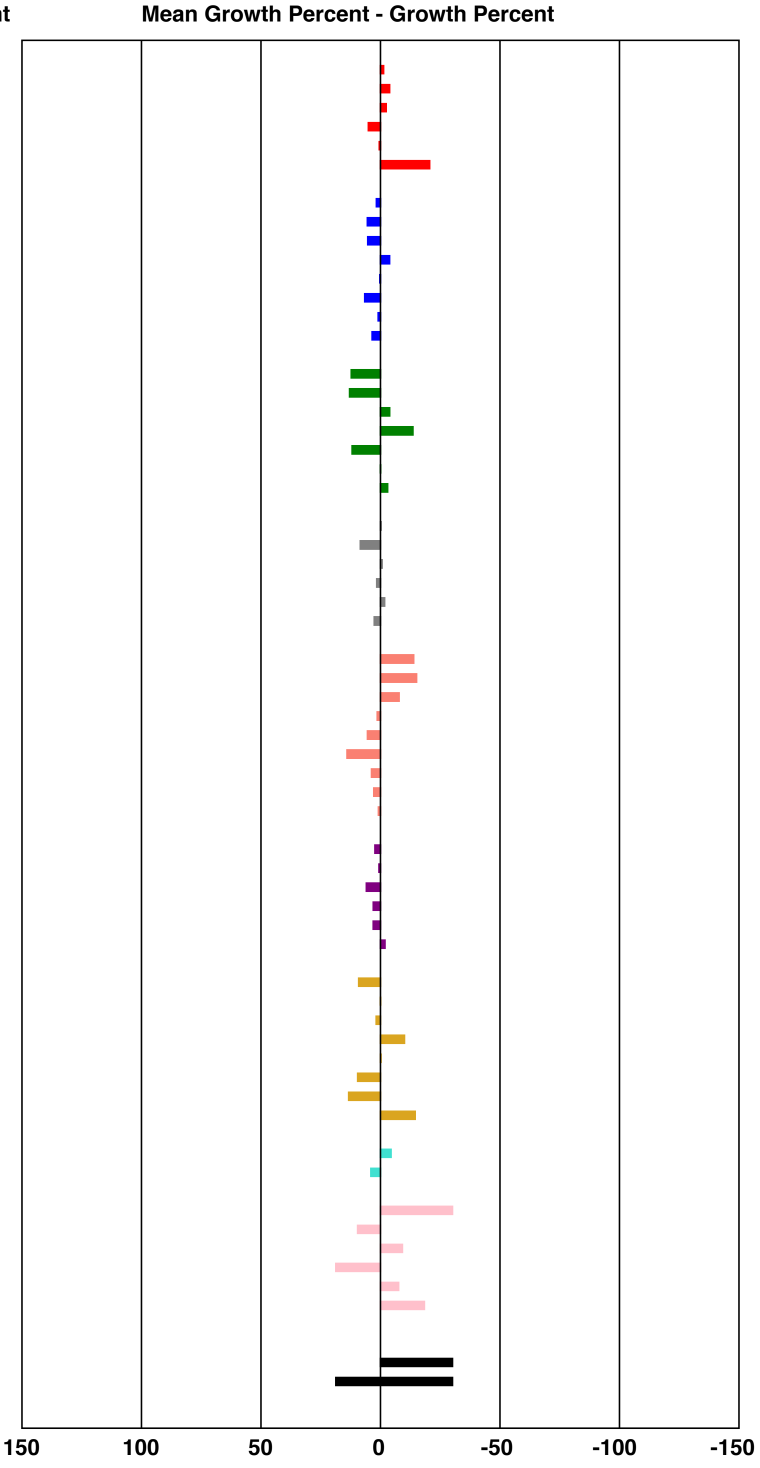

Figure S41. One dose assay of compound **13** (NSC 823913).

Developmental Therapeutics Program One Dose Mean Graph	NSC: D-823913 / 1	Conc: 1.00E-5 Molar	Test Date: Mar 23, 2020
	Experiment ID: 2003OS27		Report Date: Jun 17, 2020

Panel/Cell Line	Growth Percent
Leukemia	
CCRF-CEM	94.50
HL-60(TB)	97.72
K-562	96.23
MOLT-4	97.47
RPMI-8226	83.47
SR	76.88
Non-Small Cell Lung Cancer	
A549/ATCC	104.81
EKVX	103.05
HOP-62	95.89
HOP-92	96.61
NCI-H226	113.71
NCI-H23	94.76
NCI-H322M	80.13
NCI-H460	108.50
NCI-H522	100.83
Colon Cancer	
COLO 205	121.77
HCC-2998	91.76
HCT-116	99.61
HCT-15	92.21
HT29	110.80
KM12	85.63
SW-620	104.76
CNS Cancer	
SF-268	93.32
SF-295	106.59
SF-539	98.08
SNB-19	91.78
SNB-75	90.74
U251	101.00
Melanoma	
MALME-3M	87.86
M14	91.52
MDA-MB-435	99.35
SK-MEL-2	109.63
SK-MEL-28	86.91
SK-MEL-5	100.08
UACC-257	98.29
UACC-62	91.25
Ovarian Cancer	
IGROV1	86.48
OVCAR-3	93.19
OVCAR-4	101.40
OVCAR-5	98.60
OVCAR-8	101.15
NCI/ADR-RES	101.14
SK-OV-3	111.94
Renal Cancer	
786-0	104.10
A498	120.89
ACHN	94.14
CAKI-1	81.73
RXF 393	108.81
SN12C	91.61
TK-10	143.73
UO-31	97.05
Prostate Cancer	
PC-3	86.00
DU-145	85.74
Breast Cancer	
MCF7	50.75
MDA-MB-231/ATCC	98.22
HS 578T	104.44
BT-549	94.60
T-47D	60.16
MDA-MB-468	12.52
Mean	95.35
Delta	82.83
Range	131.21

Mean Growth Percent - Growth Percent

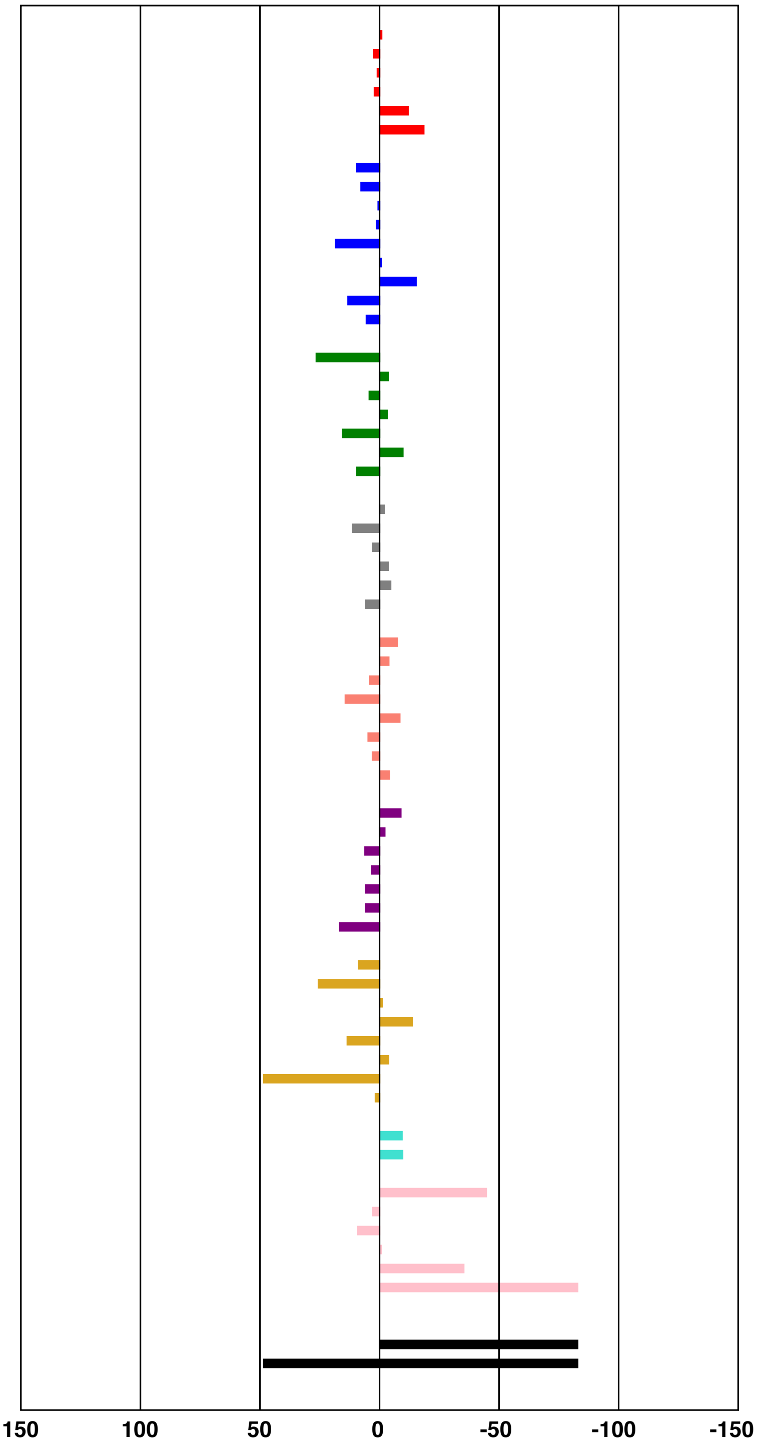

Figure S42. One dose assay of compound **14** (NSC 823914).

Developmental Therapeutics Program
One Dose Mean Graph

NSC: D-823914 / 1	Conc: 1.00E-5 Molar	Test Date: Mar 23, 2020
Experiment ID: 2003OS27		Report Date: Jun 17, 2020

Panel/Cell Line	Growth Percent
Leukemia	
CCRF-CEM	101.52
HL-60(TB)	99.32
K-562	99.26
MOLT-4	96.83
RPMI-8226	102.10
SR	103.67
Non-Small Cell Lung Cancer	
A549/ATCC	111.18
EKVX	102.57
HOP-62	107.22
HOP-92	102.01
NCI-H226	101.83
NCI-H23	99.84
NCI-H322M	101.70
NCI-H460	106.50
NCI-H522	88.95
Colon Cancer	
COLO 205	119.95
HCC-2998	98.18
HCT-116	101.24
HCT-15	109.74
HT29	114.21
KM12	102.47
SW-620	107.35
CNS Cancer	
SF-268	101.49
SF-295	106.08
SF-539	99.14
SNB-19	103.21
SNB-75	97.83
U251	101.00
Melanoma	
MALME-3M	107.10
M14	102.62
MDA-MB-435	103.83
SK-MEL-2	112.29
SK-MEL-28	111.49
SK-MEL-5	102.76
UACC-257	110.97
UACC-62	97.79
Ovarian Cancer	
IGROV1	99.34
OVCAR-3	107.67
OVCAR-4	100.45
OVCAR-5	106.11
OVCAR-8	102.73
NCI/ADR-RES	102.71
SK-OV-3	115.51
Renal Cancer	
786-0	102.07
A498	122.61
ACHN	99.48
CAKI-1	94.43
RXF 393	122.18
SN12C	101.45
TK-10	112.32
UO-31	82.53
Prostate Cancer	
PC-3	100.37
DU-145	109.62
Breast Cancer	
MCF7	89.31
MDA-MB-231/ATCC	106.83
HS 578T	99.62
BT-549	105.40
T-47D	102.58
MDA-MB-468	113.56
Mean	103.97
Delta	21.44
Range	40.08

Mean Growth Percent - Growth Percent

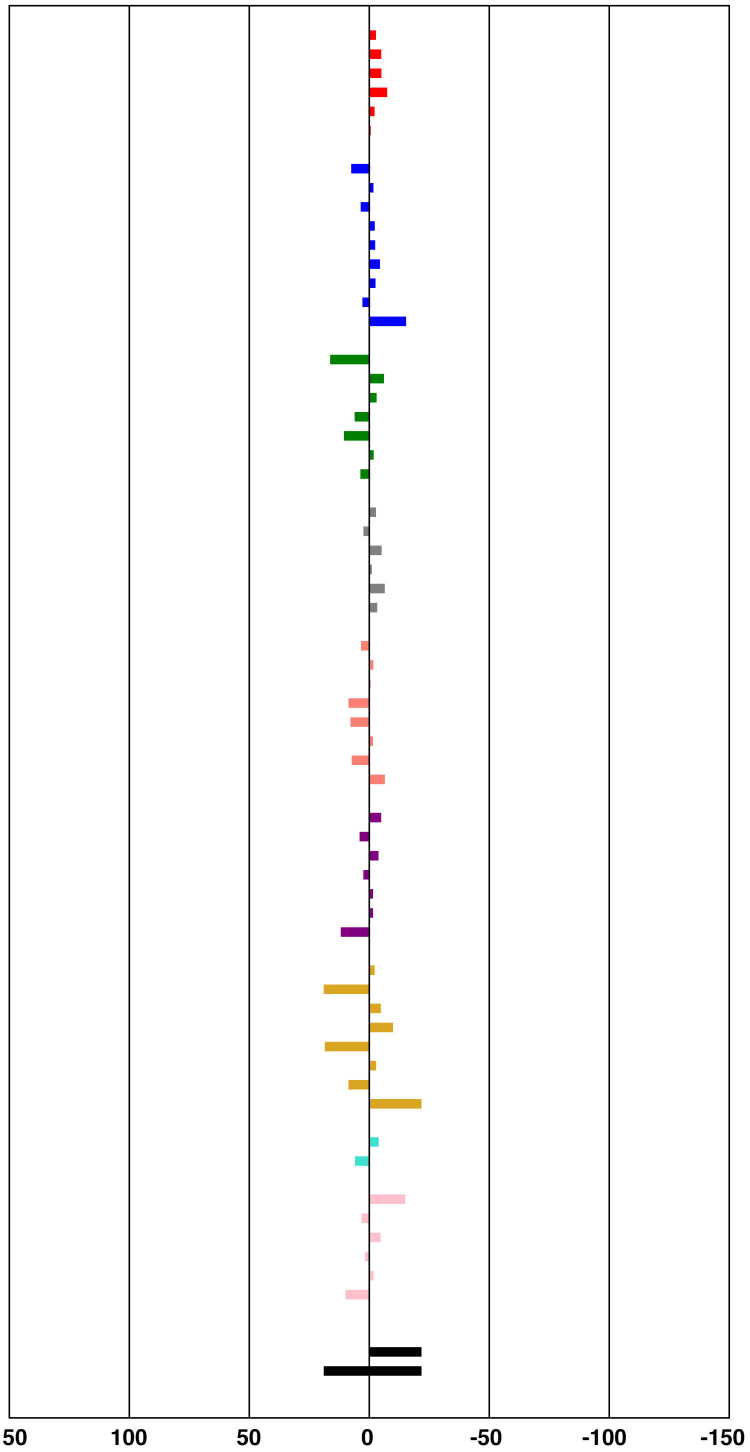

Figure S43. One dose assay of compound **15** (NSC 823916).

Developmental Therapeutics Program One Dose Mean Graph	NSC: D-823916 / 1	Conc: 1.00E-5 Molar	Test Date: Mar 23, 2020
	Experiment ID: 2003OS27		Report Date: Jun 17, 2020

Panel/Cell Line	Growth Percent
Leukemia	
CCRF-CEM	100.88
HL-60(TB)	100.43
K-562	98.43
MOLT-4	98.24
RPMI-8226	102.69
SR	107.57
Non-Small Cell Lung Cancer	
A549/ATCC	106.81
EKVX	104.59
HOP-62	111.70
HOP-92	99.15
NCI-H226	103.75
NCI-H23	101.84
NCI-H322M	106.37
NCI-H460	106.99
NCI-H522	96.38
Colon Cancer	
COLO 205	116.04
HCC-2998	109.89
HCT-116	105.24
HCT-15	106.84
HT29	107.63
KM12	100.16
SW-620	107.18
CNS Cancer	
SF-268	99.32
SF-295	103.83
SF-539	98.53
SNB-19	99.40
SNB-75	101.79
U251	102.80
Melanoma	
MALME-3M	106.16
M14	102.51
MDA-MB-435	105.33
SK-MEL-2	116.84
SK-MEL-28	111.08
SK-MEL-5	100.34
UACC-257	111.81
UACC-62	95.36
Ovarian Cancer	
IGROV1	102.39
OVCAR-3	112.02
OVCAR-4	111.06
OVCAR-5	102.31
OVCAR-8	105.54
NCI/ADR-RES	104.12
SK-OV-3	118.72
Renal Cancer	
786-0	106.40
A498	113.19
ACHN	99.69
CAKI-1	95.85
RXF 393	109.44
SN12C	99.85
TK-10	128.56
UO-31	82.52
Prostate Cancer	
PC-3	96.63
DU-145	115.46
Breast Cancer	
MCF7	98.10
MDA-MB-231/ATCC	102.52
HS 578T	105.40
BT-549	103.53
T-47D	100.97
MDA-MB-468	113.28
Mean	104.77
Delta	22.25
Range	46.04

Mean Growth Percent - Growth Percent

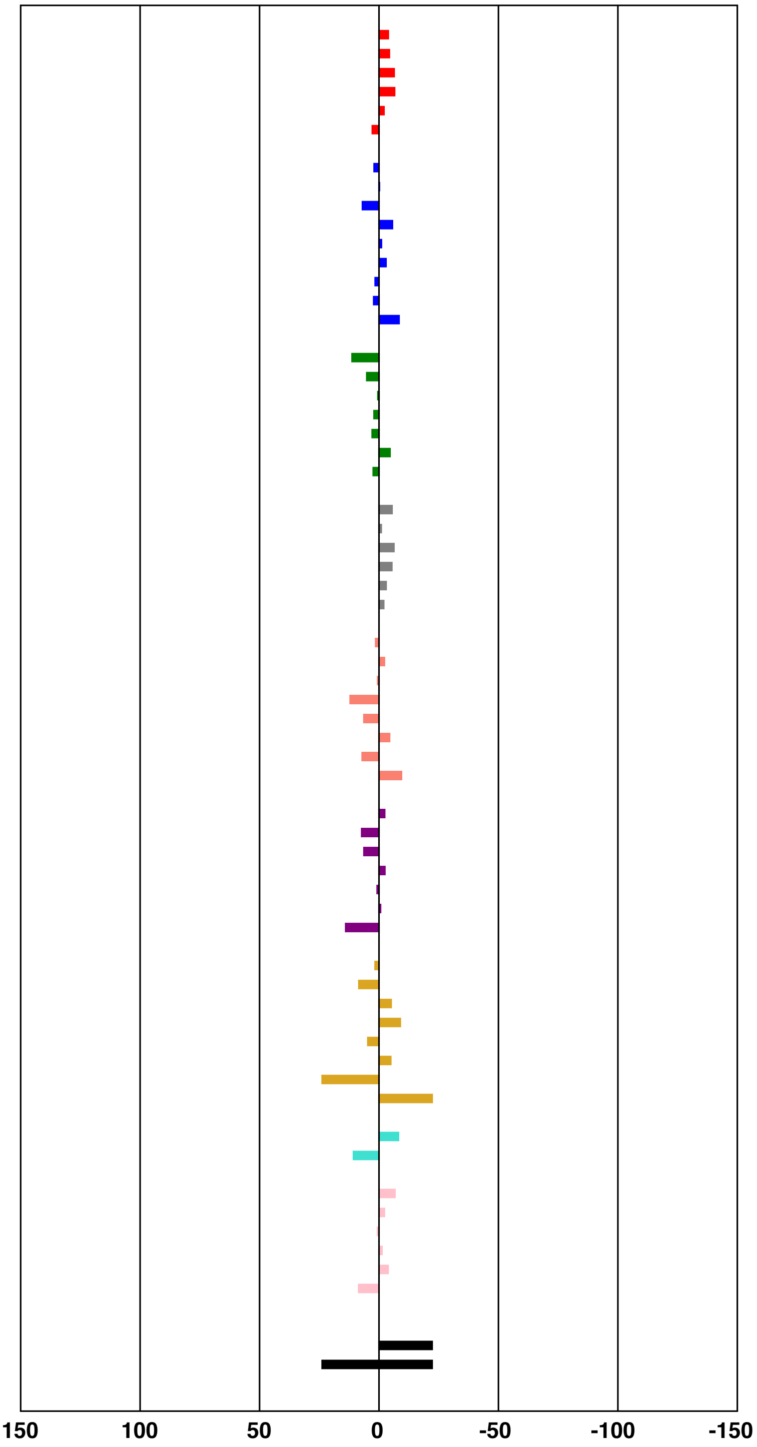

Figure S44. One dose assay of compound **16** (NSC 830872).

Developmental Therapeutics Program One Dose Mean Graph	NSC: D-830872 / 1	Conc: 1.00E-5 Molar	Test Date: Jun 14, 2021
	Experiment ID: 2106OS47		Report Date: Sep 07, 2021

Panel/Cell Line	Growth Percent
Leukemia	
CCRF-CEM	101.72
HL-60(TB)	98.88
K-562	107.73
MOLT-4	117.62
RPMI-8226	107.52
SR	106.69
Non-Small Cell Lung Cancer	
A549/ATCC	99.62
EKVX	104.00
HOP-62	113.62
NCI-H226	99.45
NCI-H23	102.93
NCI-H322M	106.57
NCI-H460	107.67
NCI-H522	101.57
Colon Cancer	
COLO 205	109.78
HCC-2998	106.84
HCT-116	110.45
HCT-15	101.57
HT29	117.89
KM12	103.86
SW-620	99.76
CNS Cancer	
SF-268	103.43
SF-295	111.61
SF-539	105.04
SNB-19	100.69
SNB-75	99.97
U251	104.80
Melanoma	
LOX IMVI	99.05
MALME-3M	104.54
M14	103.36
MDA-MB-435	102.36
SK-MEL-2	116.17
SK-MEL-28	113.36
SK-MEL-5	99.50
UACC-257	104.31
UACC-62	99.03
Ovarian Cancer	
IGROV1	115.81
OVCAR-4	105.64
OVCAR-5	111.51
OVCAR-8	102.87
NCI/ADR-RES	107.11
SK-OV-3	101.20
Renal Cancer	
786-0	108.31
A498	110.76
ACHN	110.02
CAKI-1	94.44
RXF 393	103.94
SN12C	105.61
TK-10	124.87
UO-31	90.77
Prostate Cancer	
PC-3	96.19
DU-145	110.32
Breast Cancer	
MCF7	87.80
MDA-MB-231/ATCC	109.74
HS 578T	99.88
BT-549	131.66
T-47D	98.27
MDA-MB-468	93.86
Mean	105.41
Delta	17.61
Range	43.86

Mean Growth Percent - Growth Percent

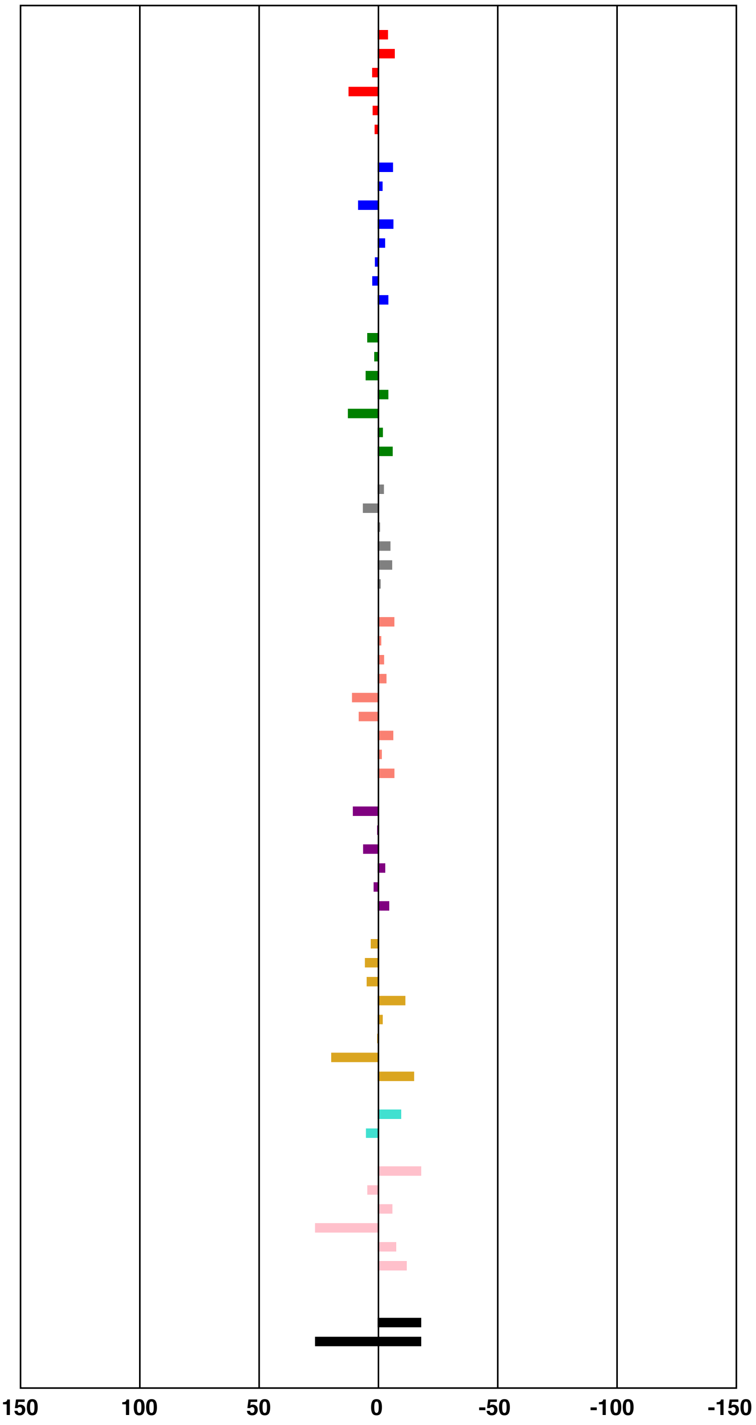

Figure S45. One dose assay of compound **17** (NSC 830870).

Developmental Therapeutics Program One Dose Mean Graph	NSC: D-830870 / 1	Conc: 1.00E-5 Molar	Test Date: Jun 14, 2021
	Experiment ID: 2106OS47		Report Date: Sep 07, 2021

Panel/Cell Line	Growth Percent
**Leukemia**	
CCRF-CEM	110.92
HL-60(TB)	111.23
K-562	111.48
MOLT-4	115.12
RPMI-8226	99.51
SR	105.21
**Non-Small Cell Lung Cancer**	
A549/ATCC	104.30
EKVX	107.40
HOP-62	116.55
NCI-H226	107.67
NCI-H23	92.70
NCI-H322M	105.70
NCI-H460	107.91
NCI-H522	102.50
**Colon Cancer**	
COLO 205	113.77
HCC-2998	112.76
HCT-116	107.04
HCT-15	105.27
HT29	109.68
KM12	103.51
SW-620	100.32
**CNS Cancer**	
SF-268	105.15
SF-295	114.87
SF-539	105.20
SNB-19	102.20
SNB-75	94.84
U251	106.55
**Melanoma**	
LOX IMVI	102.51
MALME-3M	107.41
M14	101.99
MDA-MB-435	104.59
SK-MEL-2	113.54
SK-MEL-28	113.66
SK-MEL-5	99.49
UACC-257	113.20
UACC-62	97.02
**Ovarian Cancer**	
IGROV1	111.28
OVCAR-4	104.43
OVCAR-5	110.02
OVCAR-8	104.73
NCI/ADR-RES	110.02
SK-OV-3	102.77
**Renal Cancer**	
786-0	103.65
A498	106.50
ACHN	106.89
CAKI-1	99.41
RXF 393	104.37
SN12C	105.53
TK-10	125.24
UO-31	88.88
**Prostate Cancer**	
PC-3	93.22
DU-145	109.68
**Breast Cancer**	
MCF7	85.17
MDA-MB-231/ATCC	103.76
HS 578T	113.54
BT-549	114.51
T-47D	104.09
MDA-MB-468	91.67
Mean	105.73
Delta	20.56
Range	40.07

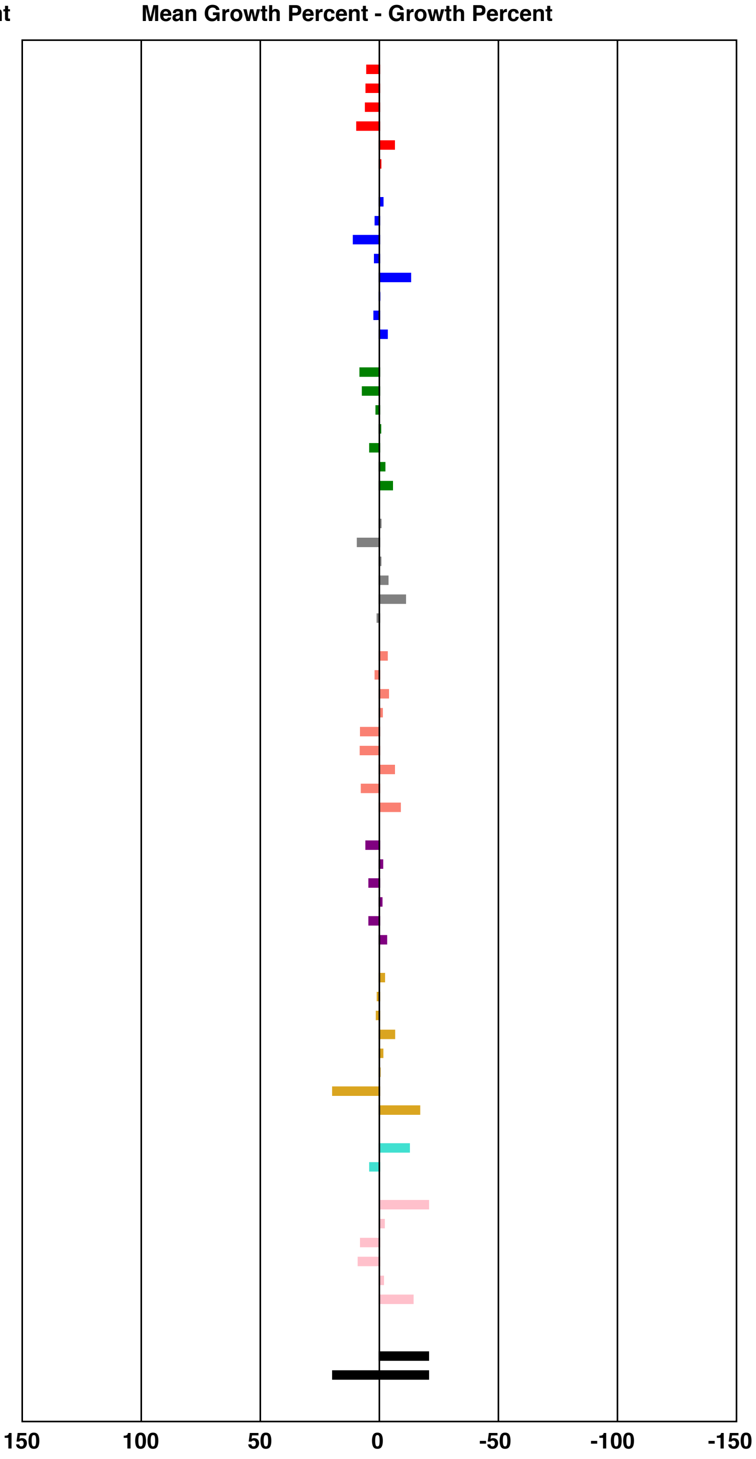

Figure S46. One dose assay of compound **18** (NSC 839958).

Developmental Therapeutics Program One Dose Mean Graph	NSC: D-839958 / 1	Conc: 1.00E-5 Molar	Test Date: Sep 26, 2022
	Experiment ID: 2209OS58		Report Date: Apr 18, 2023

Panel/Cell Line	Growth Percent
Leukemia	
CCRF-CEM	114.95
HL-60(TB)	56.57
K-562	99.37
MOLT-4	109.97
RPMI-8226	84.08
SR	112.91
Non-Small Cell Lung Cancer	
A549/ATCC	93.87
EKVX	99.91
HOP-62	95.86
HOP-92	123.20
NCI-H226	100.41
NCI-H23	93.44
NCI-H322M	52.07
NCI-H460	104.14
NCI-H522	102.29
Colon Cancer	
COLO 205	120.59
HCC-2998	109.19
HCT-116	96.32
HCT-15	97.61
HT29	108.06
KM12	92.72
SW-620	99.68
CNS Cancer	
SF-268	77.68
SF-295	98.25
SF-539	94.90
SNB-19	96.11
SNB-75	97.53
U251	97.76
Melanoma	
LOX IMVI	100.32
MALME-3M	94.27
M14	102.42
MDA-MB-435	100.23
SK-MEL-2	117.30
SK-MEL-28	72.40
SK-MEL-5	100.17
UACC-257	74.65
UACC-62	91.05
Ovarian Cancer	
IGROV1	97.59
OVCAR-3	-37.09
OVCAR-4	90.83
OVCAR-5	100.53
OVCAR-8	95.03
SK-OV-3	96.67
Renal Cancer	
786-0	102.58
A498	91.13
ACHN	95.80
CAKI-1	86.87
RXF 393	103.05
SN12C	93.85
TK-10	100.94
UO-31	77.41
Prostate Cancer	
PC-3	127.12
DU-145	102.49
Breast Cancer	
MCF7	14.10
MDA-MB-231/ATCC	92.87
HS 578T	34.76
BT-549	107.96
T-47D	-2.23
MDA-MB-468	-65.17
Mean	87.95
Delta	153.12
Range	192.29

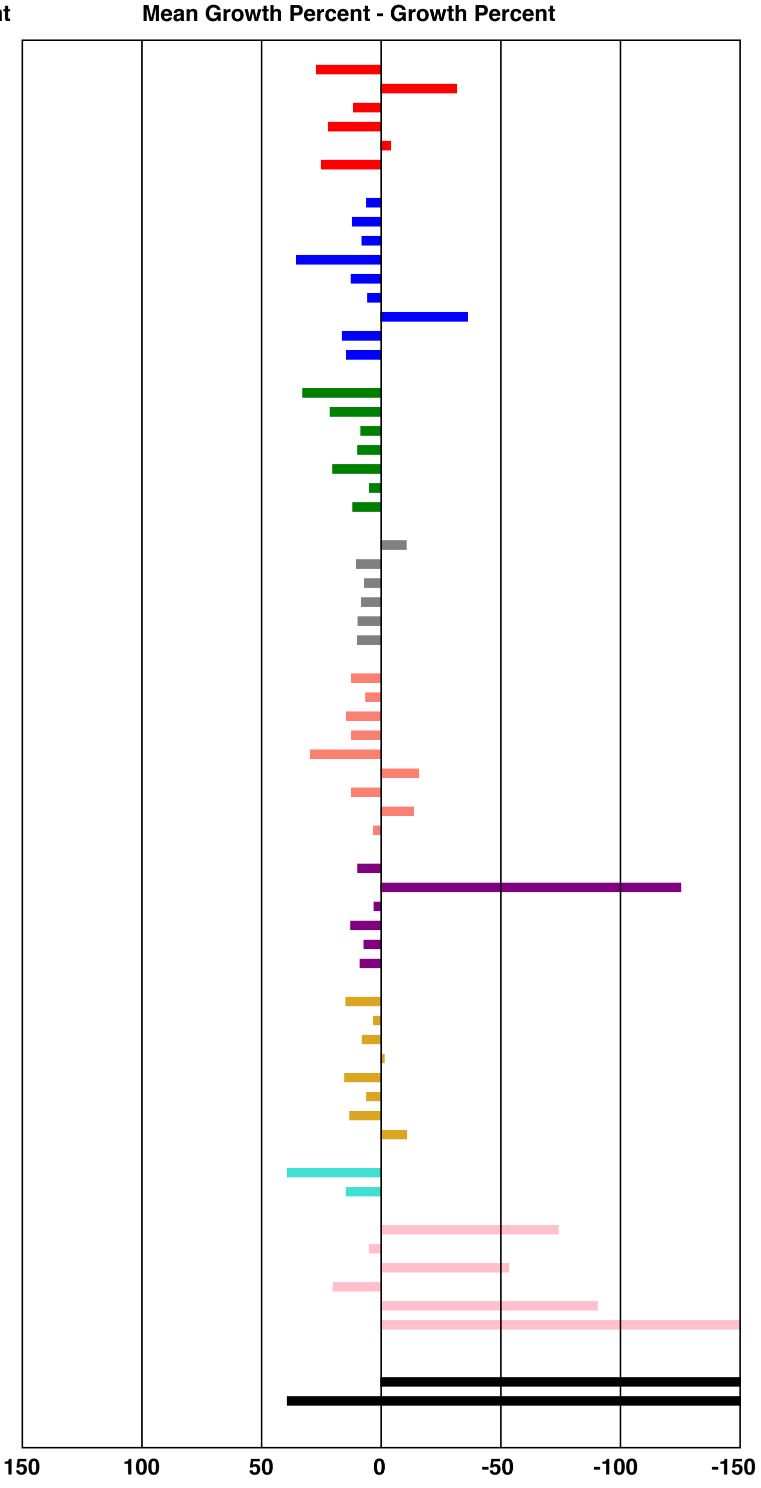

Figure S47. One dose assay of compound **19** (NSC 839959).

Developmental Therapeutics Program One Dose Mean Graph	NSC: D-839959 / 1	Conc: 1.00E-5 Molar	Test Date: Sep 26, 2022
	Experiment ID: 2209OS58		Report Date: Apr 20, 2023

Panel/Cell Line	Growth Percent
**Leukemia**	
CCRF-CEM	108.64
HL-60(TB)	39.07
K-562	99.28
MOLT-4	88.66
RPMI-8226	93.28
SR	99.48
**Non-Small Cell Lung Cancer**	
A549/ATCC	90.12
EKVX	95.95
HOP-62	96.19
HOP-92	128.17
NCI-H226	111.10
NCI-H23	92.90
NCI-H322M	72.41
NCI-H460	119.61
NCI-H522	97.77
**Colon Cancer**	
COLO 205	97.51
HCC-2998	110.19
HCT-116	113.71
HCT-15	88.92
HT29	110.22
KM12	103.93
SW-620	94.10
**CNS Cancer**	
SF-268	96.13
SF-295	101.41
SF-539	92.61
SNB-19	95.64
SNB-75	80.54
U251	90.88
**Melanoma**	
LOX IMVI	100.92
MALME-3M	95.62
M14	106.94
MDA-MB-435	100.98
SK-MEL-2	117.26
SK-MEL-28	85.86
SK-MEL-5	99.64
UACC-257	74.49
UACC-62	81.26
**Ovarian Cancer**	
IGROV1	98.93
OVCAR-3	80.20
OVCAR-4	88.29
OVCAR-5	111.79
OVCAR-8	100.21
SK-OV-3	83.86
**Renal Cancer**	
786-0	96.62
A498	88.97
ACHN	-52.38
CAKI-1	77.77
RXF 393	105.26
SN12C	93.19
TK-10	96.27
UO-31	-96.29
**Prostate Cancer**	
PC-3	122.90
DU-145	97.83
**Breast Cancer**	
MCF7	89.85
MDA-MB-231/ATCC	92.12
HS 578T	82.07
BT-549	92.43
T-47D	76.74
MDA-MB-468	46.62
Mean	88.89
Delta	185.18
Range	224.46

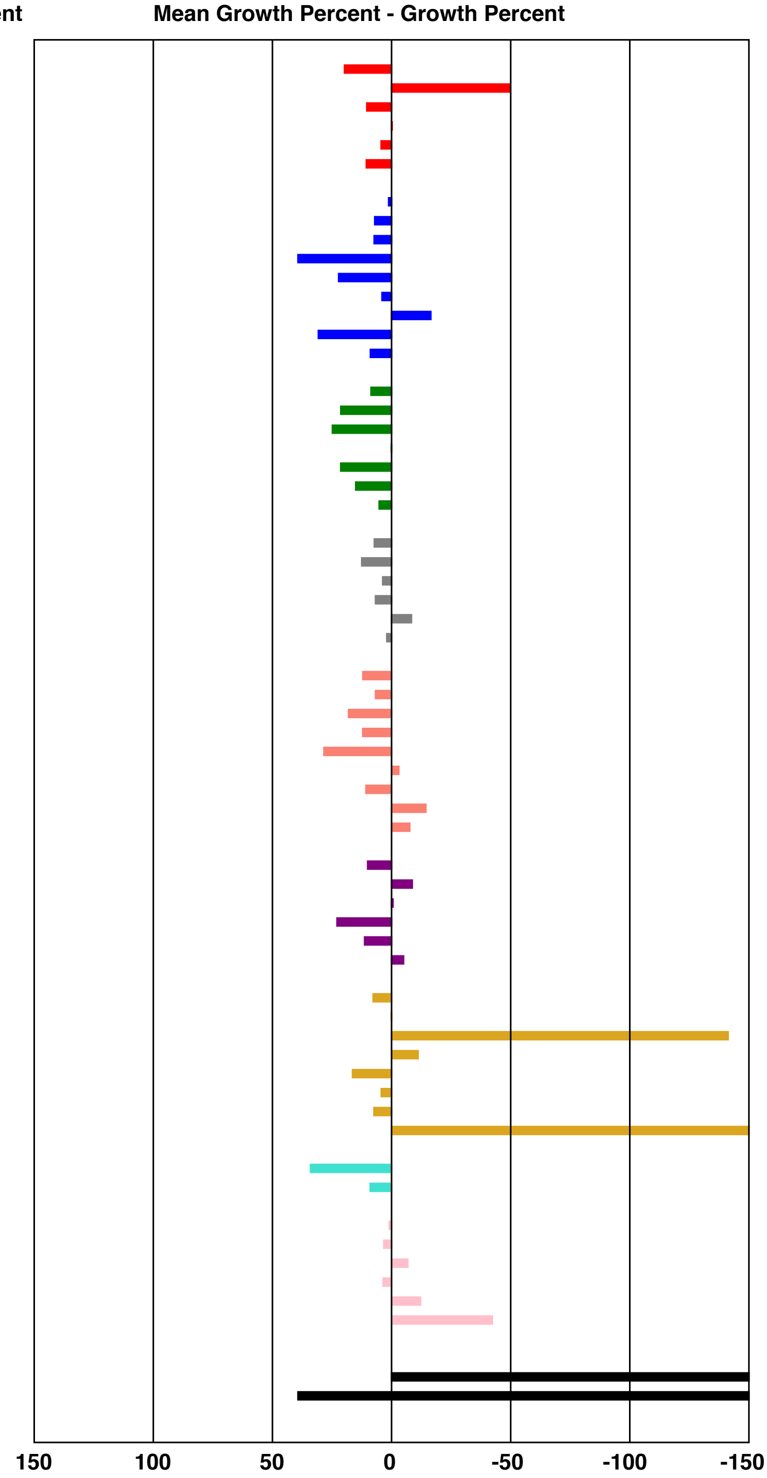

Figure S48. One dose assay of compound **20** (NSC 830869).

Developmental Therapeutics Program One Dose Mean Graph	NSC: D-830869 / 1	Conc: 1.00E-5 Molar	Test Date: Jun 14, 2021
	Experiment ID: 2106OS47		Report Date: Sep 07, 2021

Panel/Cell Line	Growth Percent
Leukemia	
CCRF-CEM	100.64
HL-60(TB)	98.59
K-562	110.55
MOLT-4	107.40
RPMI-8226	98.06
SR	98.35
Non-Small Cell Lung Cancer	
A549/ATCC	103.25
EKVX	97.35
HOP-62	109.24
HOP-92	89.03
NCI-H226	92.93
NCI-H23	96.70
NCI-H322M	97.44
NCI-H460	99.90
NCI-H522	99.25
Colon Cancer	
COLO 205	105.08
HCC-2998	106.65
HCT-116	104.19
HCT-15	97.54
HT29	116.56
KM12	97.92
SW-620	97.55
CNS Cancer	
SF-268	97.14
SF-295	103.99
SF-539	103.08
SNB-19	97.02
SNB-75	93.54
U251	100.78
Melanoma	
LOX IMVI	86.78
MALME-3M	94.99
M14	94.91
MDA-MB-435	102.79
SK-MEL-2	107.36
SK-MEL-28	106.92
SK-MEL-5	98.98
UACC-257	107.11
UACC-62	90.99
Ovarian Cancer	
IGROV1	98.89
OVCAR-4	96.05
OVCAR-5	101.66
OVCAR-8	100.93
NCI/ADR-RES	102.12
SK-OV-3	111.48
Renal Cancer	
786-0	103.10
A498	104.16
ACHN	97.95
CAKI-1	82.95
RXF 393	103.70
SN12C	91.99
TK-10	128.35
UO-31	73.91
Prostate Cancer	
PC-3	91.09
DU-145	108.89
Breast Cancer	
MCF7	66.63
MDA-MB-231/ATCC	100.69
HS 578T	87.57
BT-549	132.92
T-47D	59.35
MDA-MB-468	65.62
Mean	98.65
Delta	39.30
Range	73.57

Mean Growth Percent - Growth Percent

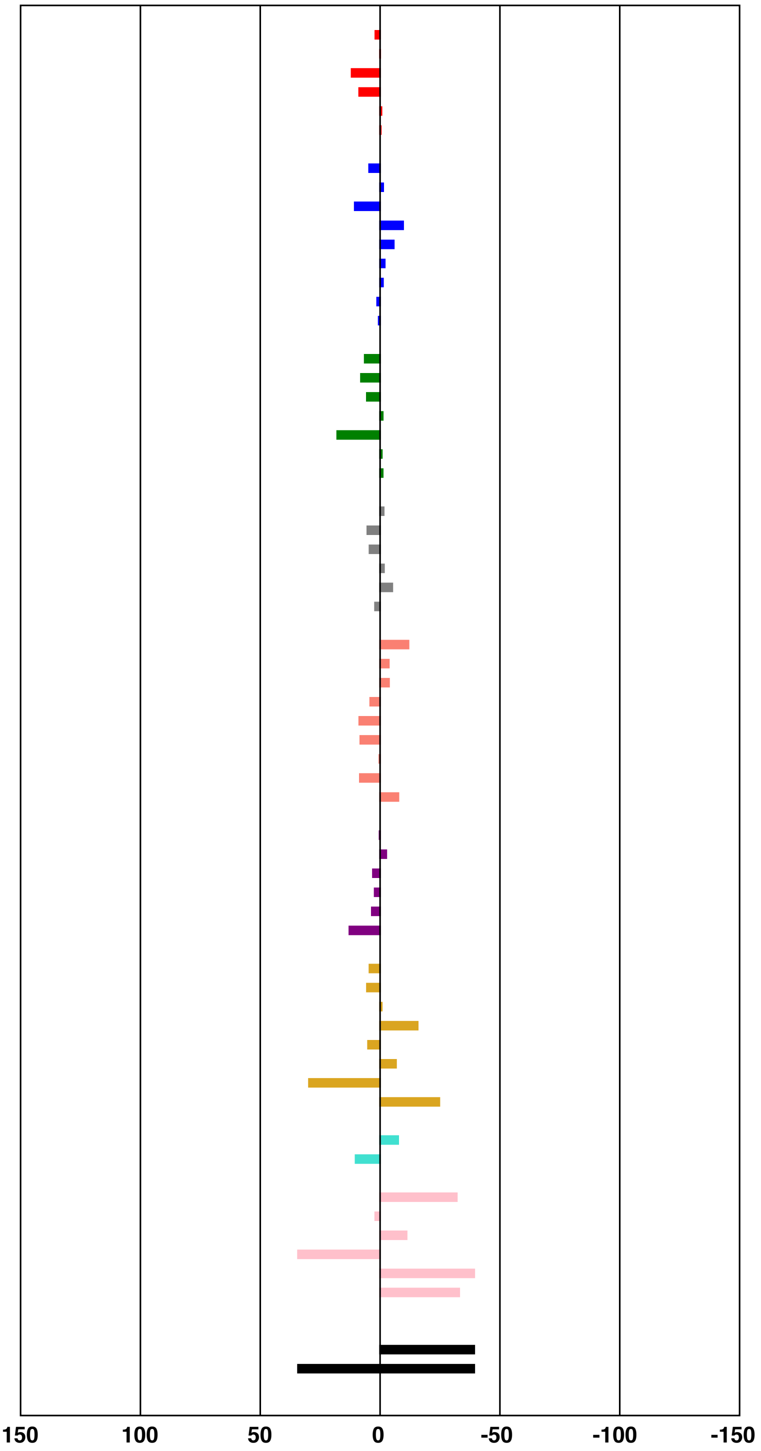

Figure S49. One dose assay of compound **21** (NSC 830868).

Developmental Therapeutics Program One Dose Mean Graph	NSC: D-830868 / 1	Conc: 1.00E-5 Molar	Test Date: Jun 14, 2021
	Experiment ID: 2106OS47		Report Date: Sep 07, 2021

Panel/Cell Line	Growth Percent
**Leukemia**	
CCRF-CEM	102.59
HL-60(TB)	108.12
K-562	107.20
MOLT-4	107.52
RPMI-8226	98.00
SR	101.70
**Non-Small Cell Lung Cancer**	
A549/ATCC	101.26
EKVX	98.18
HOP-62	115.43
HOP-92	95.53
NCI-H226	94.95
NCI-H23	100.96
NCI-H322M	97.02
NCI-H460	99.00
NCI-H522	99.70
**Colon Cancer**	
COLO 205	101.14
HCC-2998	108.13
HCT-116	105.35
HCT-15	99.31
HT29	104.98
KM12	101.15
SW-620	95.70
**CNS Cancer**	
SF-268	101.51
SF-295	101.13
SF-539	98.60
SNB-19	102.60
SNB-75	100.49
U251	104.60
**Melanoma**	
LOX IMVI	97.44
MALME-3M	101.66
M14	96.55
MDA-MB-435	102.44
SK-MEL-2	100.42
SK-MEL-28	107.59
SK-MEL-5	99.39
UACC-257	100.08
UACC-62	97.05
**Ovarian Cancer**	
IGROV1	106.80
OVCAR-4	99.60
OVCAR-5	101.48
OVCAR-8	99.85
NCI/ADR-RES	104.05
SK-OV-3	101.83
**Renal Cancer**	
786-0	105.01
A498	94.49
ACHN	98.54
CAKI-1	88.98
RXF 393	105.69
SN12C	98.78
TK-10	112.51
UO-31	84.93
**Prostate Cancer**	
PC-3	94.07
DU-145	102.20
**Breast Cancer**	
MCF7	94.03
MDA-MB-231/ATCC	102.90
HS 578T	92.68
BT-549	122.51
T-47D	99.25
MDA-MB-468	101.69
Mean	101.12
Delta	16.19
Range	37.58

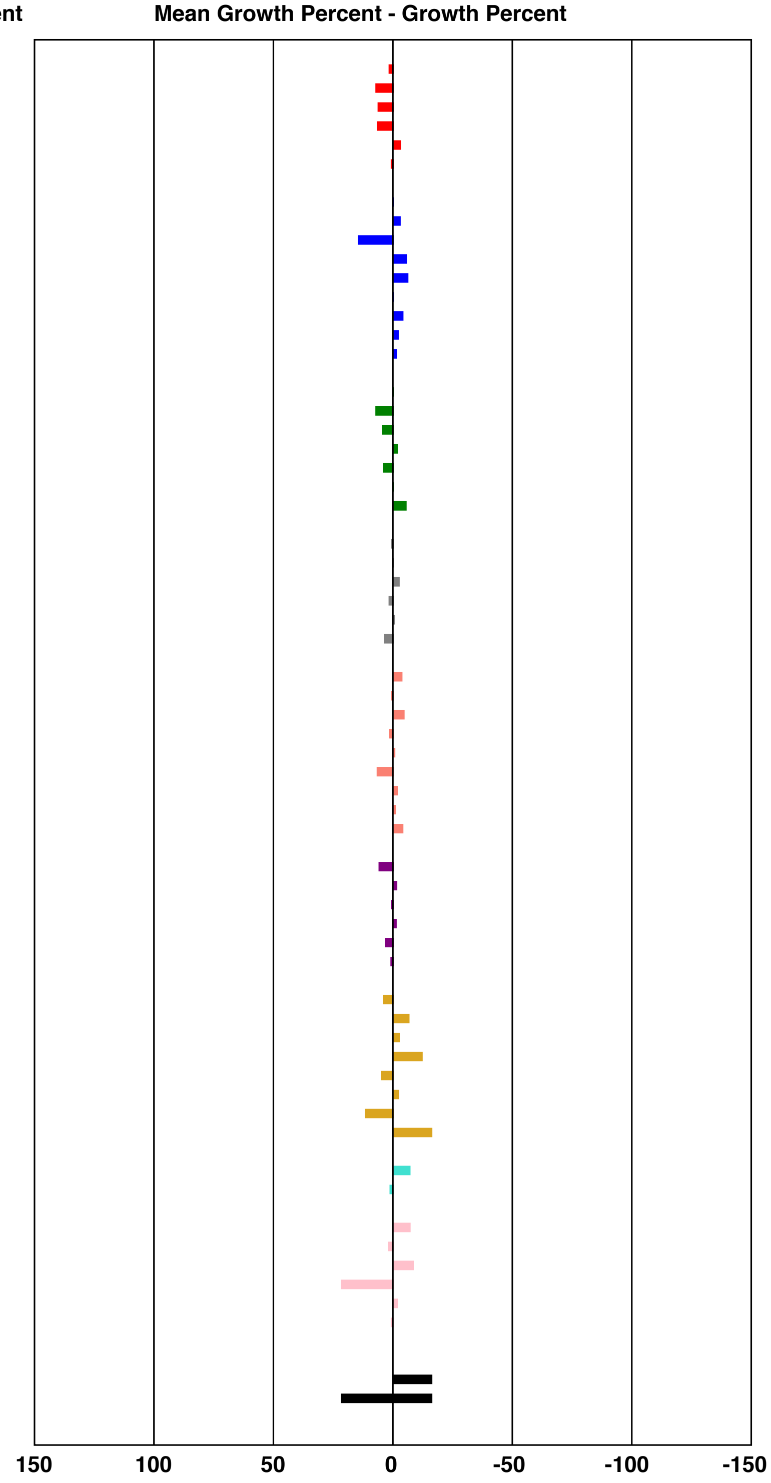

Figure S50. One dose assay of compound **22** (NSC 838517).

Developmental Therapeutics Program One Dose Mean Graph	NSC: D-838517 / 1	Conc: 1.00E-5 Molar	Test Date: Jun 13, 2022
	Experiment ID: 2206OS37		Report Date: Aug 18, 2022

Panel/Cell Line	Growth Percent
Leukemia	
CCRF-CEM	100.51
HL-60(TB)	96.99
K-562	101.24
MOLT-4	96.03
RPMI-8226	105.70
Non-Small Cell Lung Cancer	
A549/ATCC	100.38
EKVX	93.19
HOP-62	97.44
HOP-92	108.86
NCI-H226	98.57
NCI-H23	95.59
NCI-H322M	98.49
NCI-H460	102.41
NCI-H522	96.14
Colon Cancer	
COLO 205	108.44
HCC-2998	109.52
HCT-116	98.27
HCT-15	104.94
HT29	106.78
KM12	107.93
SW-620	100.13
CNS Cancer	
SF-268	98.31
SF-295	105.47
SF-539	95.71
SNB-19	97.23
SNB-75	108.00
U251	102.78
Melanoma	
LOX IMVI	93.41
MALME-3M	88.81
M14	94.62
MDA-MB-435	101.59
SK-MEL-2	105.59
SK-MEL-28	99.88
SK-MEL-5	98.39
UACC-257	98.82
UACC-62	90.28
Ovarian Cancer	
IGROV1	104.51
OVCAR-3	113.21
OVCAR-4	100.36
OVCAR-5	101.06
OVCAR-8	104.06
NCI/ADR-RES	105.12
SK-OV-3	106.57
Renal Cancer	
786-0	104.76
A498	96.74
ACHN	102.15
CAKI-1	91.42
RXF 393	94.68
SN12C	99.48
TK-10	106.76
Prostate Cancer	
PC-3	102.43
DU-145	109.11
Breast Cancer	
MCF7	96.35
HS 578T	91.53
BT-549	108.09
T-47D	95.89
MDA-MB-468	107.03
Mean	100.84
Delta	12.03
Range	24.40

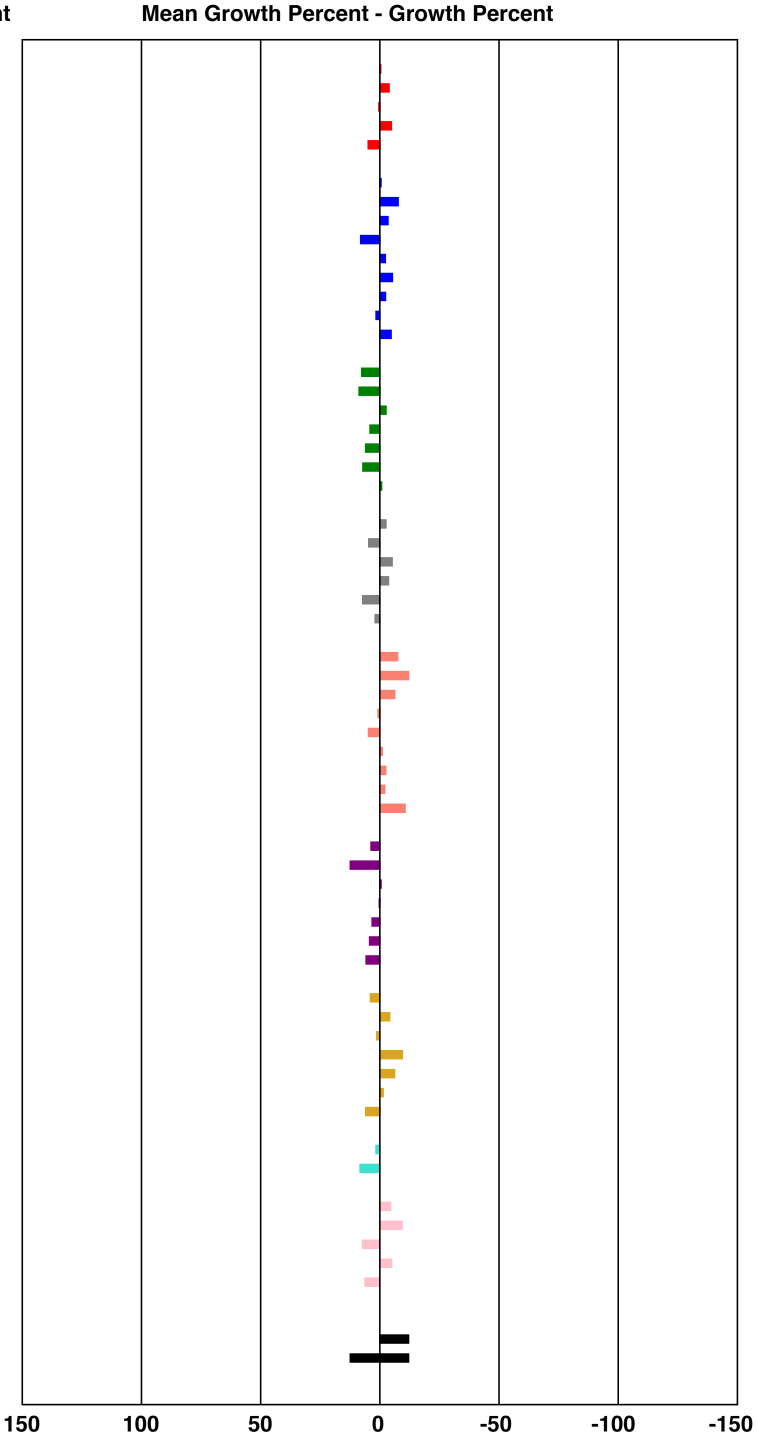

Figure S51. One dose assay of compound **23** (NSC 838518).

Developmental Therapeutics Program One Dose Mean Graph	NSC: D-838518 / 1	Conc: 1.00E-5 Molar	Test Date: Jun 13, 2022
	Experiment ID: 2206OS37		Report Date: Aug 18, 2022

Panel/Cell Line	Growth Percent
**Leukemia**	
CCRF-CEM	1.23
HL-60(TB)	-37.25
K-562	9.59
MOLT-4	-15.49
RPMI-8226	37.55
**Non-Small Cell Lung Cancer**	
A549/ATCC	99.03
EKVX	88.82
HOP-62	106.69
HOP-92	82.40
NCI-H226	85.80
NCI-H23	76.14
NCI-H322M	74.33
NCI-H460	92.23
NCI-H522	38.86
**Colon Cancer**	
COLO 205	105.75
HCC-2998	90.18
HCT-116	58.56
HCT-15	30.10
HT29	100.80
KM12	98.90
SW-620	75.12
**CNS Cancer**	
SF-268	82.41
SF-295	99.97
SF-539	81.72
SNB-19	89.47
SNB-75	103.82
U251	91.01
**Melanoma**	
LOX IMVI	-6.78
MALME-3M	71.37
M14	73.74
MDA-MB-435	92.98
SK-MEL-2	74.53
SK-MEL-28	82.52
SK-MEL-5	80.49
UACC-257	75.13
UACC-62	80.07
**Ovarian Cancer**	
IGROV1	84.71
OVCAR-3	-36.20
OVCAR-4	101.96
OVCAR-5	101.44
OVCAR-8	63.66
NCI/ADR-RES	76.15
SK-OV-3	116.44
**Renal Cancer**	
786-0	93.87
A498	90.45
ACHN	103.43
CAKI-1	89.24
RXF 393	67.80
SN12C	80.42
TK-10	98.13
**Prostate Cancer**	
PC-3	91.67
DU-145	96.52
**Breast Cancer**	
MCF7	11.64
HS 578T	78.66
BT-549	51.28
T-47D	26.30
MDA-MB-468	-59.26
Mean	68.42
Delta	127.68
Range	175.70

Mean Growth Percent - Growth Percent

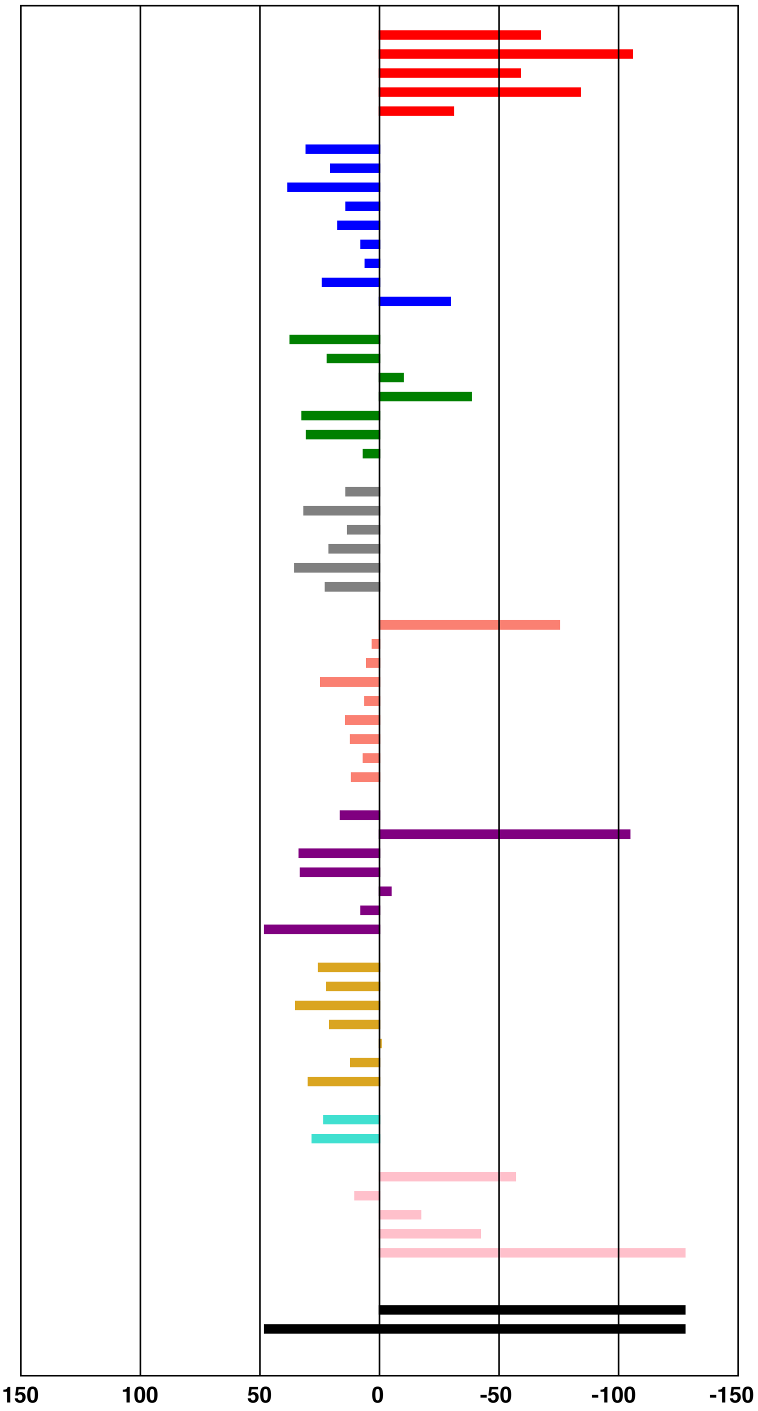

Figure S52. One dose assay of compound **24** (NSC 838520).

Developmental Therapeutics Program One Dose Mean Graph	NSC: D-838520 / 1	Conc: 1.00E-5 Molar	Test Date: Jun 13, 2022
	Experiment ID: 2206OS37		Report Date: Aug 18, 2022

Panel/Cell Line	Growth Percent
Leukemia	
CCRF-CEM	-4.01
HL-60(TB)	-45.52
K-562	-26.46
MOLT-4	-18.11
RPMI-8226	-23.78
Non-Small Cell Lung Cancer	
A549/ATCC	99.62
EKVX	76.21
HOP-62	96.81
HOP-92	-11.80
NCI-H226	71.61
NCI-H23	14.98
NCI-H322M	44.43
NCI-H460	85.06
NCI-H522	21.02
Colon Cancer	
COLO 205	114.66
HCC-2998	55.41
HCT-116	-87.08
HCT-15	-59.76
HT29	70.08
KM12	22.14
SW-620	-23.21
CNS Cancer	
SF-268	18.79
SF-295	100.51
SF-539	44.93
SNB-19	80.80
SNB-75	111.31
U251	100.65
Melanoma	
LOX IMVI	-92.60
MALME-3M	-46.99
M14	65.19
MDA-MB-435	98.19
SK-MEL-2	65.60
SK-MEL-28	26.79
SK-MEL-5	95.25
UACC-257	73.73
UACC-62	49.63
Ovarian Cancer	
IGROV1	42.31
OVCAR-3	-71.24
OVCAR-4	53.35
OVCAR-5	113.80
OVCAR-8	-20.96
NCI/ADR-RES	-46.65
SK-OV-3	128.19
Renal Cancer	
786-0	91.68
A498	106.08
ACHN	99.01
CAKI-1	93.28
RXF 393	-34.66
SN12C	46.45
TK-10	107.34
Prostate Cancer	
PC-3	-13.63
DU-145	15.88
Breast Cancer	
MCF7	-73.91
HS 578T	25.47
BT-549	5.18
T-47D	-47.36
MDA-MB-468	-56.86
Mean	32.05
Delta	124.65
Range	220.79

Mean Growth Percent - Growth Percent

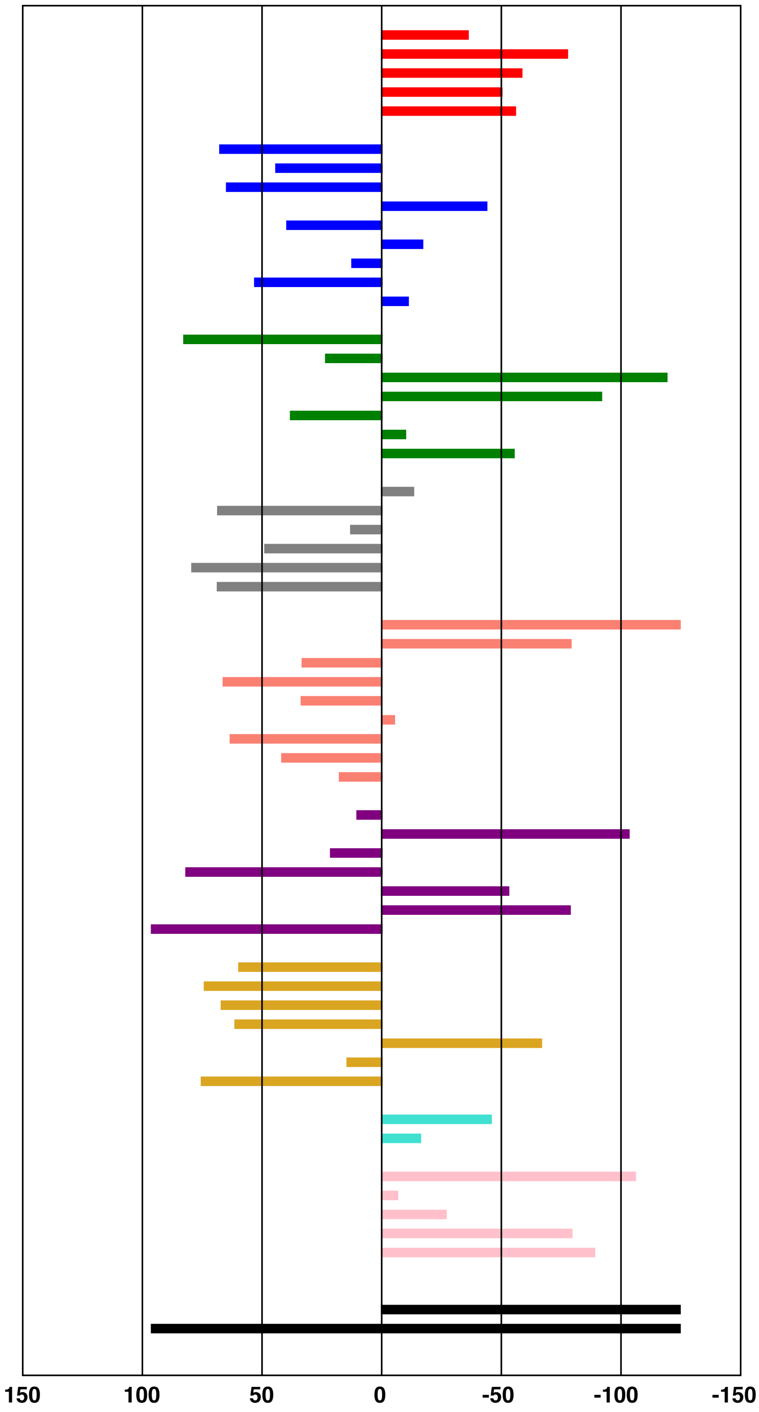

Figure S53. One dose assay of compound **25** (NSC 839956).

Developmental Therapeutics Program
One Dose Mean Graph

NSC: D-839956 / 1 | **Conc:** 1.00E-5 Molar | **Test Date:** Sep 26, 2022
Experiment ID: 2209OS58 | **Report Date:** Apr 20, 2023

Panel/Cell Line	Growth Percent
**Leukemia**	
CCRF-CEM	0.64
HL-60(TB)	-12.92
K-562	-21.77
MOLT-4	-13.61
RPMI-8226	-47.17
SR	-38.37
**Non-Small Cell Lung Cancer**	
A549/ATCC	100.70
EKVX	104.68
HOP-62	111.21
HOP-92	-1.36
NCI-H226	-65.81
NCI-H23	-78.56
NCI-H322M	82.08
NCI-H460	90.71
NCI-H522	-75.38
**Colon Cancer**	
COLO 205	118.25
HCC-2998	101.28
HCT-116	-45.16
HCT-15	-13.02
HT29	106.98
KM12	30.95
SW-620	-69.22
**CNS Cancer**	
SF-268	-75.55
SF-295	111.87
SF-539	11.80
SNB-19	92.97
SNB-75	110.54
U251	99.58
**Melanoma**	
LOX IMVI	-73.70
MALME-3M	-94.56
M14	-80.47
MDA-MB-435	61.49
SK-MEL-2	54.80
SK-MEL-28	-81.34
SK-MEL-5	-98.08
UACC-257	71.58
UACC-62	-28.02
**Ovarian Cancer**	
IGROV1	-8.79
OVCAR-3	-93.14
OVCAR-4	-17.30
OVCAR-5	103.33
OVCAR-8	-37.70
SK-OV-3	114.54
**Renal Cancer**	
786-0	85.76
A498	116.42
ACHN	96.79
CAKI-1	61.62
RXF 393	58.11
SN12C	-17.95
TK-10	106.28
UO-31	74.11
**Prostate Cancer**	
PC-3	67.48
DU-145	102.98
**Breast Cancer**	
MCF7	-53.35
MDA-MB-231/ATCC	-65.04
HS 578T	-14.78
BT-549	-85.67
T-47D	-2.05
MDA-MB-468	-50.86
Mean	16.76
Delta	114.84
Range	216.33

Mean Growth Percent - Growth Percent

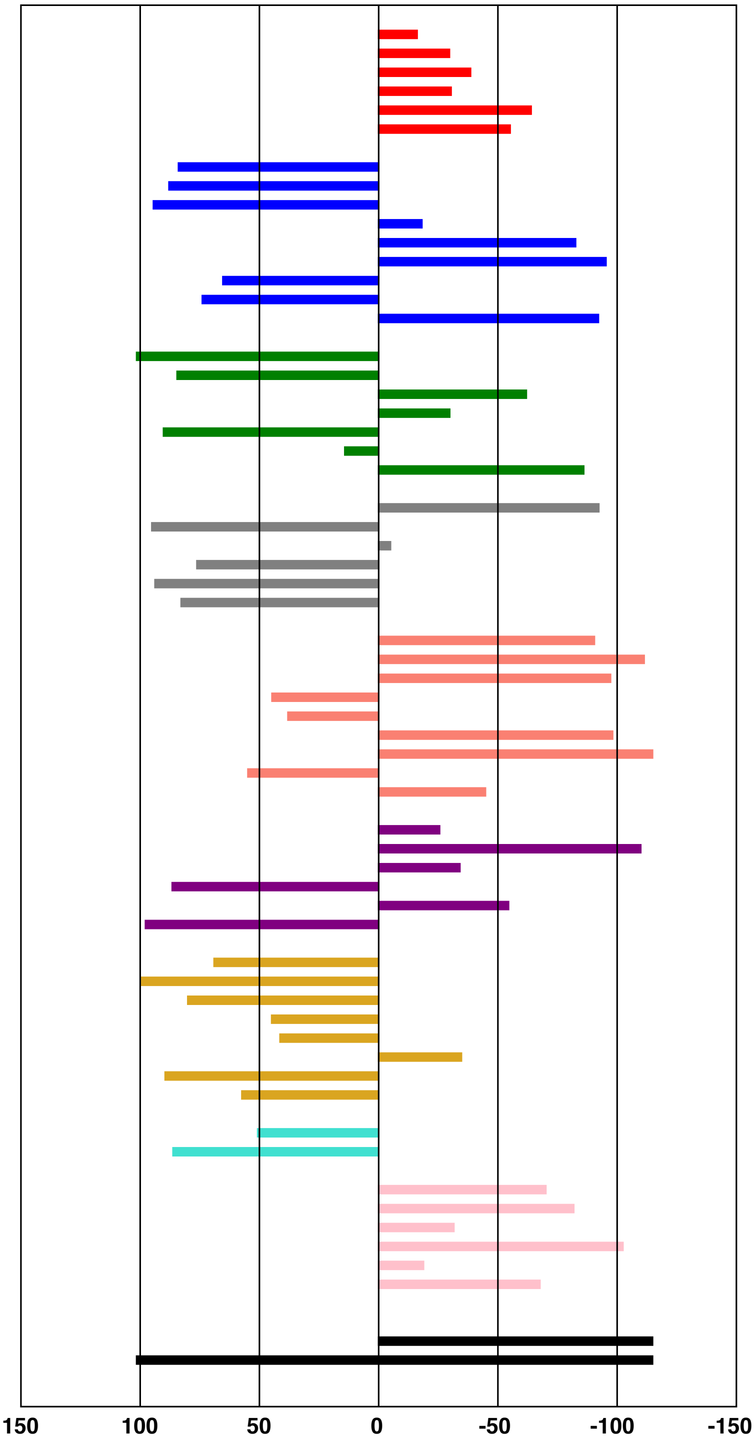

Figure S54. One dose assay of compound **26** (NSC 839957).

Developmental Therapeutics Program One Dose Mean Graph	NSC: D-839957 / 1	Conc: 1.00E-5 Molar	Test Date: Sep 26, 2022
	Experiment ID: 2209OS58		Report Date: Apr 18, 2023

Panel/Cell Line	Growth Percent
**Leukemia**	
CCRF-CEM	-7.77
HL-60(TB)	-57.94
K-562	-29.03
MOLT-4	-17.64
RPMI-8226	-34.43
SR	-39.41
**Non-Small Cell Lung Cancer**	
A549/ATCC	99.41
EKVX	92.07
HOP-62	94.18
HOP-92	-4.27
NCI-H226	70.91
NCI-H23	-67.87
NCI-H322M	65.09
NCI-H460	71.18
NCI-H522	-79.80
**Colon Cancer**	
COLO 205	63.66
HCC-2998	64.01
HCT-116	-84.26
HCT-15	-4.99
HT29	86.17
KM12	-15.55
SW-620	-84.87
**CNS Cancer**	
SF-268	-16.55
SF-295	109.14
SF-539	23.74
SNB-19	78.49
SNB-75	73.06
U251	83.05
**Melanoma**	
LOX IMVI	-80.84
MALME-3M	-80.41
M14	-26.51
MDA-MB-435	72.51
SK-MEL-2	8.15
SK-MEL-28	-93.56
SK-MEL-5	89.20
UACC-257	59.42
UACC-62	-58.57
**Ovarian Cancer**	
IGROV1	-7.85
OVCAR-3	-87.85
OVCAR-4	55.50
OVCAR-5	100.38
OVCAR-8	-17.80
SK-OV-3	97.46
**Renal Cancer**	
786-0	62.44
A498	115.10
ACHN	28.16
CAKI-1	35.39
RXF 393	-1.40
SN12C	39.85
TK-10	109.14
UO-31	43.21
**Prostate Cancer**	
PC-3	15.19
DU-145	37.92
**Breast Cancer**	
MCF7	-23.58
MDA-MB-231/ATCC	-64.88
HS 578T	42.51
BT-549	-80.67
T-47D	11.23
MDA-MB-468	-69.63
Mean	14.56
Delta	108.12
Range	208.66

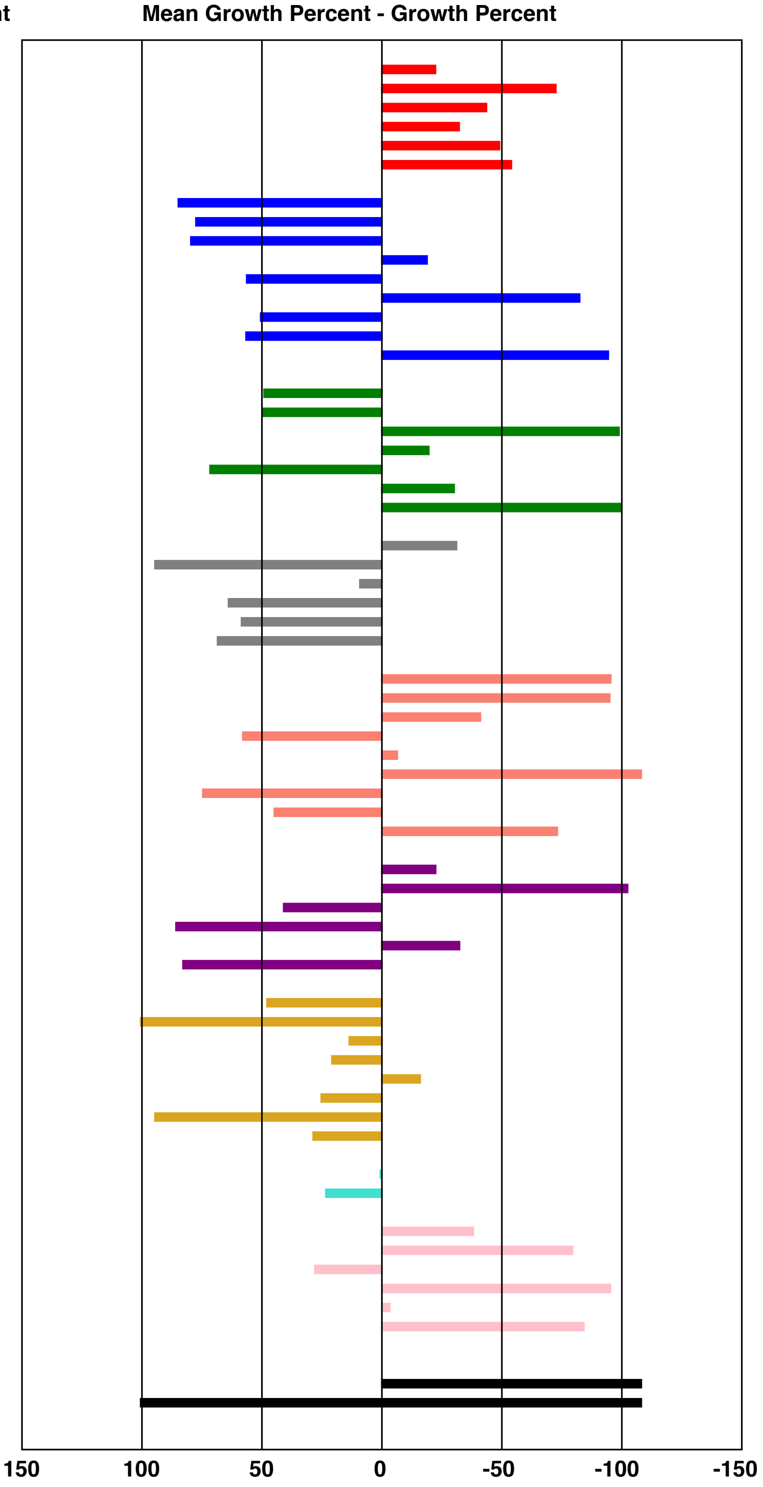

Figure S55. One dose assay of compound **27** (NSC 823917).

Developmental Therapeutics Program One Dose Mean Graph	NSC: D-823917 / 1	Conc: 1.00E-5 Molar	Test Date: Mar 23, 2020
	Experiment ID: 2003OS27		Report Date: Jun 17, 2020

Panel/Cell Line	Growth Percent
Leukemia	
CCRF-CEM	97.66
HL-60(TB)	98.28
K-562	103.10
MOLT-4	104.99
RPMI-8226	81.52
SR	114.18
Non-Small Cell Lung Cancer	
A549/ATCC	113.73
EKVX	80.92
HOP-62	97.25
HOP-92	93.13
NCI-H226	82.67
NCI-H23	92.45
NCI-H322M	100.20
NCI-H460	101.79
NCI-H522	75.76
Colon Cancer	
COLO 205	124.40
HCC-2998	108.62
HCT-116	95.23
HCT-15	101.45
HT29	121.27
KM12	98.70
SW-620	104.30
CNS Cancer	
SF-268	96.10
SF-295	102.75
SF-539	100.58
SNB-19	98.43
SNB-75	84.20
U251	104.92
Melanoma	
MALME-3M	96.70
M14	103.02
MDA-MB-435	108.23
SK-MEL-2	106.03
SK-MEL-28	107.82
SK-MEL-5	94.30
UACC-257	110.84
UACC-62	94.57
Ovarian Cancer	
IGROV1	61.65
OVCAR-3	110.11
OVCAR-4	107.10
OVCAR-5	101.59
OVCAR-8	100.18
NCI/ADR-RES	103.45
SK-OV-3	101.77
Renal Cancer	
786-0	108.03
A498	110.27
ACHN	88.56
CAKI-1	82.43
RXF 393	104.27
SN12C	95.37
TK-10	189.17
UO-31	67.88
Prostate Cancer	
PC-3	97.65
DU-145	112.91
Breast Cancer	
MCF7	73.41
MDA-MB-231/ATCC	85.19
HS 578T	97.08
BT-549	93.92
T-47D	88.91
MDA-MB-468	109.13
Mean	99.83
Delta	38.18
Range	127.52

Mean Growth Percent - Growth Percent

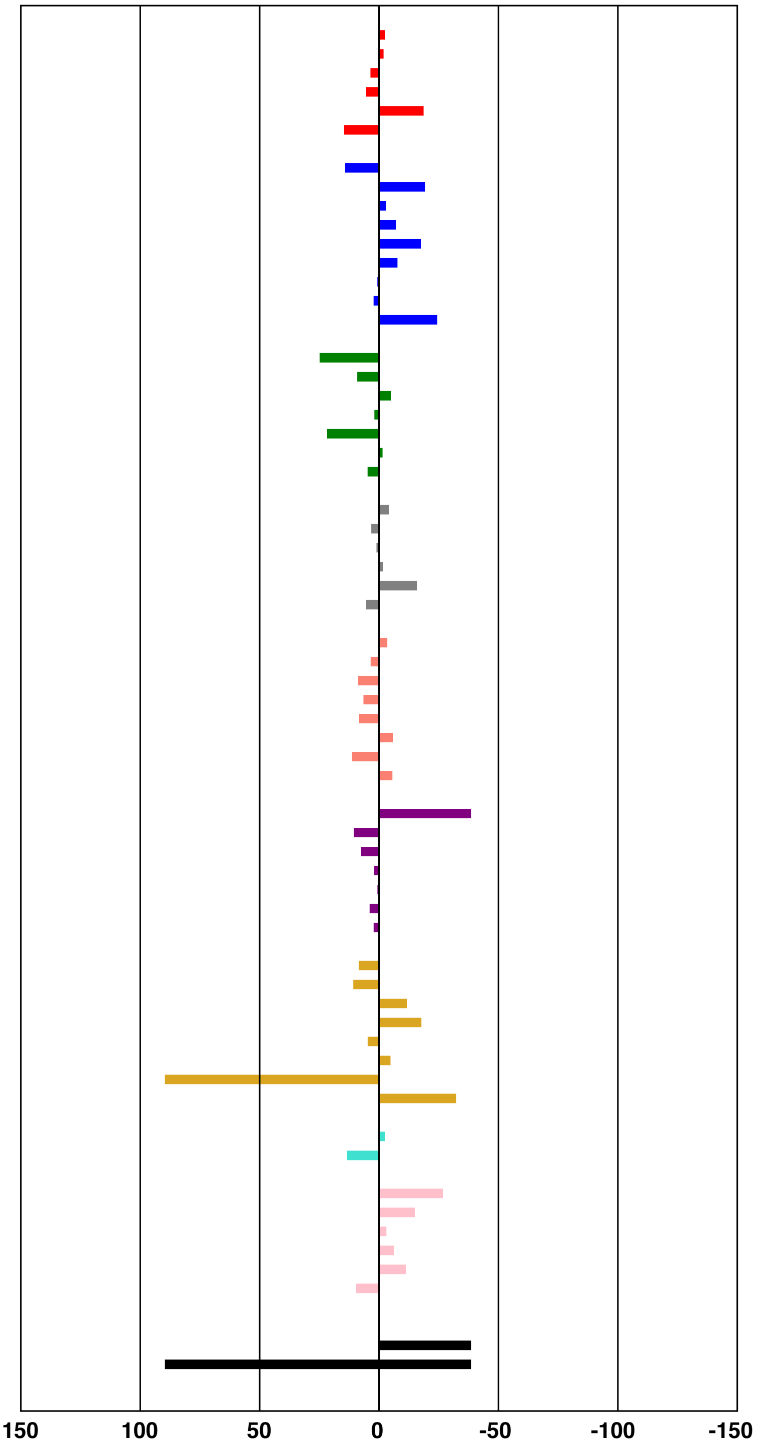

Figure S56. One dose assay of compound **28** (NSC 823918).

Developmental Therapeutics Program

One Dose Mean Graph

**NSC:** D-823918 / 1	**Conc:** 1.00E-5 Molar	**Test Date:** Mar 23, 2020
**Experiment ID:** 2003OS27		**Report Date:** Jun 17, 2020

Panel/Cell Line	Growth Percent
Leukemia	
CCRF-CEM	94.78
HL-60(TB)	98.45
K-562	98.09
MOLT-4	95.37
RPMI-8226	85.83
SR	94.45
Non-Small Cell Lung Cancer	
A549/ATCC	110.69
EKVX	79.68
HOP-62	100.05
HOP-92	93.34
NCI-H226	85.71
NCI-H23	94.71
NCI-H322M	98.73
NCI-H460	105.17
NCI-H522	87.93
Colon Cancer	
COLO 205	127.39
HCC-2998	106.32
HCT-116	98.95
HCT-15	106.52
HT29	122.60
KM12	99.94
SW-620	108.47
CNS Cancer	
SF-268	101.48
SF-295	99.48
SF-539	100.20
SNB-19	96.76
SNB-75	94.20
U251	108.45
Melanoma	
MALME-3M	100.27
M14	97.46
MDA-MB-435	103.47
SK-MEL-2	101.83
SK-MEL-28	110.50
SK-MEL-5	95.88
UACC-257	107.86
UACC-62	91.60
Ovarian Cancer	
IGROV1	66.79
OVCAR-3	104.12
OVCAR-4	107.03
OVCAR-5	109.35
OVCAR-8	106.18
NCI/ADR-RES	103.33
SK-OV-3	106.22
Renal Cancer	
786-0	104.32
A498	116.39
ACHN	97.23
CAKI-1	92.32
RXF 393	114.55
SN12C	97.16
TK-10	189.82
UO-31	66.52
Prostate Cancer	
PC-3	94.00
DU-145	110.36
Breast Cancer	
MCF7	85.14
MDA-MB-231/ATCC	78.48
HS 578T	103.48
BT-549	95.38
T-47D	93.26
MDA-MB-468	111.92
Mean	100.95
Delta	34.43
Range	123.30

Mean Growth Percent - Growth Percent

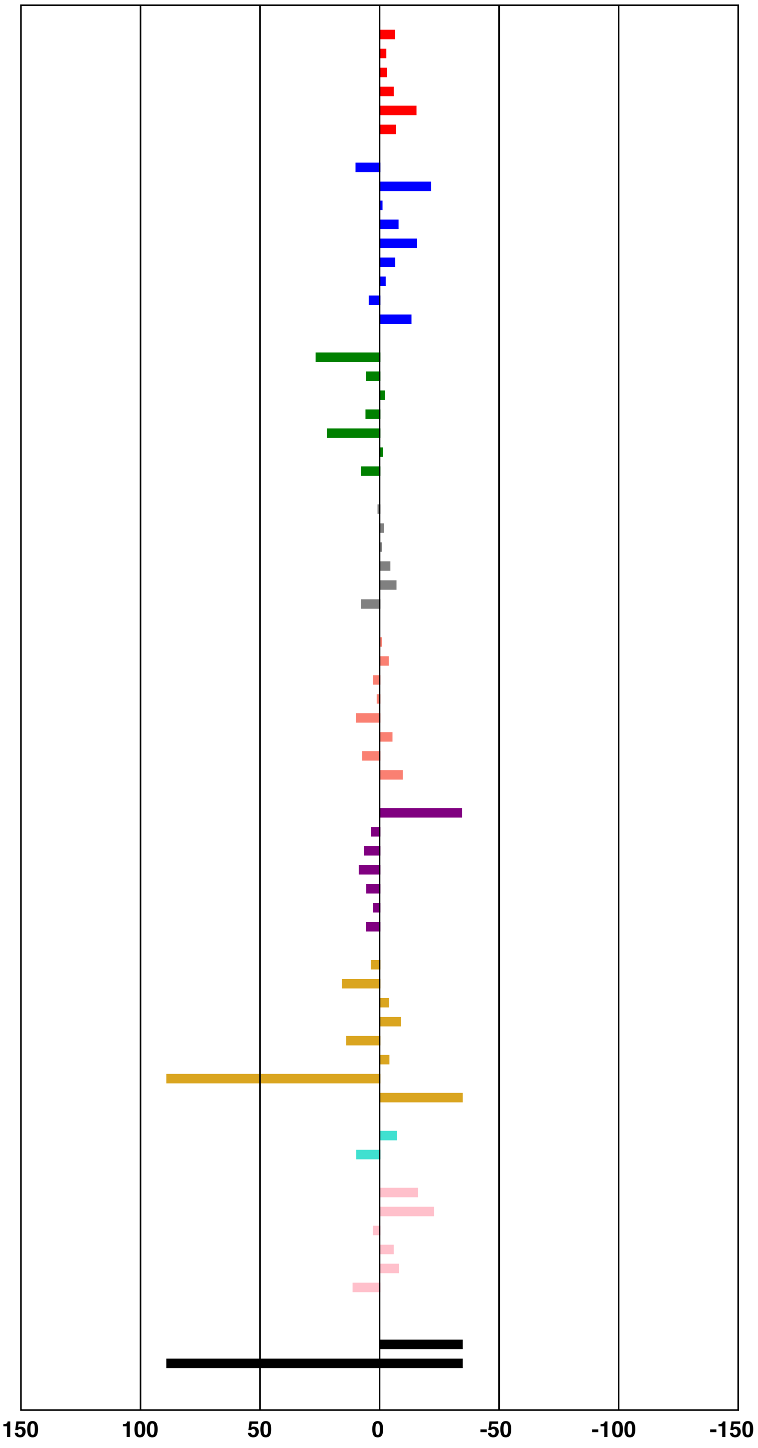

Figure S57. 5-Dose assays of compound **24** (NSC 838520).

National Cancer Institute Developmental Therapeutics Program
In-Vitro Testing Results

NSC : D - 838520 / 1	Experiment ID : 2210NS63	Test Type : 08	Units : Molar
Report Date : May 08, 2023	Test Date : October 24, 2022	QNS :	MC :
COMI : 7JP	Stain Reagent : SRB Dual-Pass Related	SSPL : 0J6G	

	Log10 Concentration														
	Time		Mean Optical Densities					Percent Growth							
Panel/Cell Line	Zero	Ctrl	-8.0	-7.0	-6.0	-5.0	-4.0	-8.0	-7.0	-6.0	-5.0	-4.0	GI50	TGI	LC50
Leukemia															
CCRF-CEM	0.724	3.070	3.111	3.011	2.952	1.263	0.532	102	97	95	23	-27	4.21E-6	2.91E-5	> 1.00E-4
HL-60(TB)	0.460	2.189	2.106	2.126	1.965	0.629	0.361	95	96	87	10	-22	3.01E-6	2.04E-5	> 1.00E-4
K-562	0.167	1.933	1.860	1.934	1.807	0.738	0.220	96	100	93	32	3	5.11E-6	> 1.00E-4	> 1.00E-4
MOLT-4	0.480	2.708	2.682	2.623	2.498	0.774	0.425	99	96	91	13	-11	3.34E-6	3.43E-5	> 1.00E-4
RPMI-8226	0.830	3.032	3.032	3.044	2.929	1.177	0.572	100	101	95	16	-31	3.71E-6	2.17E-5	> 1.00E-4
SR	0.467	2.332	2.238	2.349	2.245	1.494	0.400	95	101	95	55	-14	1.18E-5	6.21E-5	> 1.00E-4
Non-Small Cell Lung Cancer															
A549/ATCC	0.549	2.605	2.510	2.572	2.458	2.451	0.190	95	98	93	93	-65	1.86E-5	3.85E-5	7.99E-5
EKVX	0.651	2.026	1.840	1.900	1.940	1.689	0.011	86	91	94	75	-98	1.40E-5	2.72E-5	5.27E-5
HOP-62	0.997	2.726	2.615	2.653	2.621	2.549	0.020	94	96	94	90	-98	1.63E-5	3.01E-5	5.55E-5
HOP-92	1.505	2.385	2.266	2.253	2.226	1.851	0.134	86	85	82	39	-91	5.60E-6	2.00E-5	4.84E-5
NCI-H226	0.833	2.111	2.024	2.085	2.076	1.933	0.247	93	98	97	86	-70	1.70E-5	3.55E-5	7.41E-5
NCI-H23	0.725	2.418	2.411	2.416	2.350	2.139	0.030	100	100	96	84	-96	1.54E-5	2.92E-5	5.55E-5
NCI-H322M	0.821	2.325	2.259	2.314	2.234	1.668	0.024	96	99	94	56	-97	1.10E-5	2.33E-5	4.93E-5
NCI-H460	0.262	2.344	2.278	2.300	2.189	1.815	0.047	97	98	93	75	-82	1.43E-5	2.99E-5	6.23E-5
NCI-H522	1.053	2.807	2.650	2.650	2.642	2.414	0.127	91	91	91	78	-88	1.47E-5	2.94E-5	5.90E-5
Colon Cancer															
COLO 205	0.836	2.458	2.433	2.503	2.448	2.345	0.010	98	103	99	93	-99	1.68E-5	3.05E-5	5.57E-5
HCC-2998	0.963	2.977	2.832	2.995	3.040	1.905	0.012	93	101	103	47	-99	8.76E-6	2.10E-5	4.62E-5
HCT-116	0.277	2.564	2.550	2.576	2.416	1.377	0.043	99	101	94	48	-85	9.09E-6	2.30E-5	5.48E-5
HCT-15	0.533	2.956	2.844	2.929	2.865	1.396	0.102	95	99	96	36	-81	5.79E-6	2.02E-5	5.43E-5
HT29	0.437	2.383	2.330	2.449	2.367	1.920	0.181	97	103	99	76	-59	1.56E-5	3.67E-5	8.62E-5
KM12	1.009	3.401	3.393	3.398	3.400	3.129	0.365	100	100	100	89	-64	1.79E-5	3.81E-5	8.12E-5
SW-620	0.360	2.526	2.456	2.526	2.420	2.002	0.095	97	100	95	76	-74	1.49E-5	3.22E-5	6.95E-5
CNS Cancer															
SF-268	1.154	2.902	2.717	2.810	2.873	2.621	0.358	89	95	98	84	-69	1.67E-5	3.54E-5	7.51E-5
SF-295	0.871	2.714	2.562	2.675	2.692	2.588	0.059	92	98	99	93	-93	1.70E-5	3.16E-5	5.86E-5
SF-539	0.924	2.689	2.552	2.647	2.595	2.485	0.010	92	98	95	88	-99	1.60E-5	2.96E-5	5.48E-5
SNB-19	0.648	2.093	1.994	2.107	1.985	1.902	0.033	93	101	93	87	-95	1.59E-5	3.00E-5	5.66E-5
SNB-75	1.022	1.818	1.649	1.704	1.661	1.276	0.023	79	86	80	32	-98	4.22E-6	1.76E-5	4.28E-5
U251	0.458	2.326	2.245	2.232	2.195	2.125	0.138	96	95	93	89	-70	1.76E-5	3.64E-5	7.50E-5
Melanoma															
LOX IMVI	0.309	1.909	1.874	1.867	1.772	1.323	0.014	98	97	91	63	-96	1.21E-5	2.50E-5	5.16E-5
MALME-3M	0.804	1.798	1.738	1.760	1.720	1.634	0.009	94	96	92	83	-99	1.53E-5	2.87E-5	5.39E-5
M14	0.544	2.127	2.098	2.091	1.997	1.895	0.026	98	98	92	85	-95	1.57E-5	2.97E-5	5.62E-5
MDA-MB-435	0.500	2.319	2.219	2.227	2.115	1.963	0.044	94	95	89	80	-91	1.50E-5	2.94E-5	5.75E-5
SK-MEL-28	0.682	2.132	2.057	2.159	2.090	1.218	0.010	95	102	97	37	-99	6.06E-6	1.87E-5	4.38E-5
SK-MEL-5	1.080	3.327	3.239	3.308	3.208	3.233	0.006	96	99	95	96	-99	1.72E-5	3.09E-5	5.58E-5
UACC-257	1.448	2.991	2.894	2.923	2.900	2.736	0.099	94	96	94	84	-93	1.55E-5	2.97E-5	5.70E-5
UACC-62	1.047	2.955	2.846	2.910	2.808	2.638	0.015	94	98	92	83	-99	1.53E-5	2.87E-5	5.41E-5
Ovarian Cancer															
IGROV1	0.467	1.930	1.897	1.939	1.820	1.049	0.061	98	101	92	40	-87	6.40E-6	2.06E-5	5.10E-5
OVCAR-3	0.495	1.721	1.718	1.764	1.704	0.288	0.031	100	104	99	-42	-94	2.22E-6	5.03E-6	1.43E-5
OVCAR-4	1.073	2.323	2.272	2.314	2.271	1.734	0.057	96	99	96	53	-95	1.05E-5	2.28E-5	4.98E-5
OVCAR-5	0.659	1.763	1.776	1.807	1.697	1.720	0.065	101	104	94	96	-90	1.77E-5	3.28E-5	6.08E-5
NCI/ADR-RES	0.648	1.811	1.743	1.766	1.729	1.406	0.179	94	96	93	65	-72	1.29E-5	2.98E-5	6.88E-5
SK-OV-3	1.200	2.339	2.186	2.253	2.269	2.290	0.006	87	92	94	96	-100	1.71E-5	3.09E-5	5.58E-5
Renal Cancer															
786-0	0.817	2.787	2.606	2.724	2.702	2.531	0.059	91	97	96	87	-93	1.61E-5	3.05E-5	5.78E-5
A498	1.373	1.966	1.893	1.946	1.896	1.858	0.288	88	97	88	82	-79	1.57E-5	3.22E-5	6.60E-5
ACHN	0.402	1.808	1.817	1.885	1.821	1.699	-0.004	101	105	101	92	-100	1.66E-5	3.02E-5	5.49E-5
CAKI-1	1.093	2.593	2.493	2.590	2.566	2.551	0.039	93	100	98	97	-96	1.75E-5	3.18E-5	5.76E-5
RXF 393	1.007	1.675	1.666	1.751	1.649	1.088	0.031	99	111	96	12	-97	3.54E-6	1.29E-5	3.71E-5
SN12C	0.623	2.141	2.021	2.049	1.988	1.700	0.006	92	94	90	71	-99	1.33E-5	2.61E-5	5.15E-5
TK-10	1.080	2.750	2.622	2.677	2.705	2.591	0.046	92	96	97	90	-96	1.65E-5	3.06E-5	5.68E-5
UO-31	0.856	2.594	2.473	2.489	2.487	2.291	.	93	94	94	83	-100	1.51E-5	2.83E-5	5.32E-5
Prostate Cancer															
PC-3	0.543	2.420	2.374	2.323	2.248	1.046	0.149	98	95	91	27	-73	4.34E-6	1.86E-5	5.93E-5
DU-145	0.411	1.809	1.870	1.937	1.883	1.001	0.009	104	109	105	42	-98	7.52E-6	2.00E-5	4.55E-5
Breast Cancer															
MCF7	0.351	1.787	1.675	1.804	1.675	0.121	0.106	92	101	92	-66	-70	1.85E-6	3.84E-6	7.96E-6
MDA-MB-231/ATCC	0.595	1.431	1.426	1.417	1.384	0.802	0.086	99	98	94	25	-86	4.33E-6	1.67E-5	4.76E-5
HS 578T	1.261	2.504	2.435	2.483	2.471	1.544	0.601	94	98	97	23	-52	4.31E-6	2.01E-5	9.30E-5
BT-549	1.500	2.452	2.382	2.412	2.442	2.170	0.031	93	96	99	70	-98	1.32E-5	2.62E-5	5.19E-5
T-47D	1.260	2.716	2.598	2.506	2.648	0.864	0.326	92	86	95	-31	-74	2.28E-6	5.65E-6	2.72E-5
MDA-MB-468	0.741	1.572	1.547	1.569	1.480	0.407	0.082	97	100	89	-45	-89	1.95E-6	4.61E-6	1.29E-5

Figure S58. 5-Dose assays of compound **25** (NSC 839956).

National Cancer Institute Developmental Therapeutics Program
In-Vitro Testing Results

NSC : D - 839956 / 1	Experiment ID : 2302NS97	Test Type : 08	Units : Molar
Report Date : April 26, 2023	Test Date : February 13, 2023	QNS :	MC :
COMI : 16JPG	Stain Reagent : SRB Dual-Pass Related	SSPL : 0J6G	

			Log10 Concentration													
	Time		Mean Optical Densities					Percent Growth								
Panel/Cell Line	Zero	Ctrl	-8.0	-7.0	-6.0	-5.0	-4.0	-8.0	-7.0	-6.0	-5.0	-4.0	GI50	TGI	LC50
**Leukemia**															
CCRF-CEM	0.464	2.168	2.137	2.181	2.101	0.825	0.589	98	101	96	21	7	4.12E-6	> 1.00E-4	> 1.00E-4
HL-60(TB)	0.609	1.865	1.697	1.803	1.783	1.869	0.279	87	95	94	100	-54	2.12E-5	4.46E-5	9.38E-5
K-562	0.229	1.895	2.039	1.861	1.869	1.572	0.225	109	98	98	81	-2	2.35E-5	9.47E-5	> 1.00E-4
MOLT-4	0.612	1.976	2.066	2.022	1.949	0.859	0.546	107	103	98	18	-11	3.99E-6	4.21E-5	> 1.00E-4
RPMI-8226	0.702	2.776	2.720	2.730	2.670	2.273	0.495	97	98	95	76	-29	1.76E-5	5.25E-5	> 1.00E-4
SR	0.235	0.852	0.695	0.778	0.762	0.247	0.155	75	88	85	2	-34	2.66E-6	1.13E-5	> 1.00E-4
**Non-Small Cell Lung Cancer**															
A549/ATCC	0.534	2.847	2.761	2.731	2.694	2.721	2.603	96	95	93	95	89	> 1.00E-4	> 1.00E-4	> 1.00E-4
EKVX	0.708	2.583	2.446	2.485	2.463	2.663	1.246	93	95	94	104	29	5.22E-5	> 1.00E-4	> 1.00E-4
HOP-62	0.461	1.954	1.938	1.923	1.957	1.841	0.186	99	98	100	92	-60	1.90E-5	4.05E-5	8.63E-5
HOP-92	1.259	1.943	1.870	1.784	1.795	1.709	0.168	89	77	78	66	-87	1.27E-5	2.70E-5	5.75E-5
NCI-H226	0.796	2.276	2.118	2.034	1.924	1.792	0.116	89	84	76	67	-85	1.30E-5	2.76E-5	5.86E-5
NCI-H23	0.842	2.926	2.834	2.845	2.829	2.743	0.309	96	96	95	91	-63	1.85E-5	3.89E-5	8.20E-5
NCI-H322M	0.815	2.445	2.322	2.288	2.271	2.235	0.274	92	90	89	87	-66	1.75E-5	3.70E-5	7.82E-5
NCI-H460	0.244	2.412	2.630	2.604	2.720	2.414	0.028	110	109	114	100	-89	1.84E-5	3.39E-5	6.24E-5
NCI-H522	1.418	3.140	3.058	3.069	3.090	2.900	0.198	95	96	97	86	-86	1.62E-5	3.16E-5	6.17E-5
**Colon Cancer**															
COLO 205	0.221	1.150	1.163	1.157	1.173	1.171	0.147	101	101	102	102	-33	2.43E-5	5.67E-5	> 1.00E-4
HCC-2998	0.513	2.440	2.513	2.604	2.416	2.500	0.199	104	108	99	103	-61	2.10E-5	4.24E-5	8.55E-5
HCT-116	0.308	2.481	2.482	2.497	2.474	2.484	0.123	100	101	100	100	-60	2.05E-5	4.21E-5	8.63E-5
HCT-15	0.287	2.535	2.382	2.275	2.285	2.323	0.265	93	88	89	91	-8	2.59E-5	8.36E-5	> 1.00E-4
HT29	0.384	2.511	2.394	2.531	2.426	2.425	0.198	94	101	96	96	-49	2.08E-5	4.61E-5	> 1.00E-4
KM12	0.466	2.575	2.531	2.503	2.571	2.502	0.028	98	97	100	97	-94	1.75E-5	3.21E-5	5.88E-5
SW-620	0.280	2.320	2.426	2.416	2.411	2.181	0.101	105	105	104	93	-64	1.88E-5	3.91E-5	8.13E-5
**CNS Cancer**															
SF-268	0.825	2.441	2.434	2.364	2.450	2.278	0.055	100	95	101	90	-93	1.65E-5	3.09E-5	5.80E-5
SF-295	0.937	2.955	2.921	2.840	2.941	2.911	2.300	98	94	99	98	68	> 1.00E-4	> 1.00E-4	> 1.00E-4
SF-539	0.757	2.433	2.483	2.438	2.346	2.349	0.002	103	100	95	95	-100	1.70E-5	3.07E-5	5.55E-5
SNB-19	0.542	2.064	2.013	2.007	1.991	1.960	0.326	97	96	95	93	-40	2.11E-5	5.02E-5	> 1.00E-4
SNB-75	1.800	3.214	3.111	2.958	3.044	2.974	0.102	93	82	88	83	-94	1.54E-5	2.94E-5	5.62E-5
U251	0.474	2.502	2.436	2.383	2.309	2.262	0.096	97	94	91	88	-80	1.69E-5	3.35E-5	6.65E-5
**Melanoma**															
LOX IMVI	0.215	1.942	2.042	1.989	1.918	1.462	0.211	106	103	99	72	-2	1.99E-5	9.37E-5	> 1.00E-4
MALME-3M	0.757	1.714	1.624	1.610	1.605	1.622	0.003	91	89	89	90	-100	1.63E-5	2.99E-5	5.48E-5
M14	0.515	1.888	1.854	1.843	1.806	1.757	0.199	98	97	94	90	-61	1.85E-5	3.94E-5	8.42E-5
MDA-MB-435	0.488	2.347	2.362	2.332	2.252	2.184	0.016	101	99	95	91	-97	1.66E-5	3.06E-5	5.64E-5
SK-MEL-2	1.941	3.023	3.045	3.003	3.058	2.988	0.297	102	98	103	97	-85	1.81E-5	3.41E-5	6.44E-5
SK-MEL-28	0.604	1.962	1.944	1.893	1.896	1.859	0.014	99	95	95	92	-98	1.67E-5	3.06E-5	5.61E-5
SK-MEL-5	0.778	2.954	2.562	2.728	2.653	2.429	.	82	90	86	76	-100	1.40E-5	2.70E-5	5.20E-5
UACC-257	1.447	3.020	2.946	2.889	2.912	2.942	0.447	95	92	93	95	-69	1.88E-5	3.79E-5	7.65E-5
UACC-62	0.869	2.792	2.607	2.587	2.554	2.478	0.041	90	89	88	84	-95	1.54E-5	2.94E-5	5.58E-5
**Ovarian Cancer**															
IGROV1	0.788	2.683	2.732	2.708	2.700	2.591	0.020	103	101	101	95	-98	1.71E-5	3.12E-5	5.67E-5
OVCAR-3	0.480	1.690	1.732	1.693	1.734	1.528	0.004	104	100	104	87	-99	1.57E-5	2.93E-5	5.44E-5
OVCAR-4	0.865	2.332	2.325	2.274	2.185	2.052	0.007	100	96	90	81	-99	1.48E-5	2.81E-5	5.33E-5
OVCAR-5	0.539	1.579	1.450	1.422	1.405	1.382	0.185	88	85	83	81	-66	1.63E-5	3.57E-5	7.82E-5
OVCAR-8	0.529	2.793	2.723	2.715	2.717	2.695	0.285	97	97	97	96	-46	2.10E-5	4.73E-5	> 1.00E-4
NCI/ADR-RES	0.489	1.989	1.878	1.932	1.910	1.803	0.367	93	96	95	88	-25	2.16E-5	6.00E-5	> 1.00E-4
SK-OV-3	0.717	1.959	1.983	1.879	1.983	2.141	0.198	102	94	102	115	-72	2.22E-5	4.10E-5	7.59E-5
**Renal Cancer**															
786-0	0.787	2.557	2.443	2.463	2.446	2.478	0.084	94	95	94	96	-89	1.76E-5	3.29E-5	6.13E-5
A498	1.177	2.110	2.132	2.151	2.106	2.024	2.150	102	104	100	91	104	> 1.00E-4	> 1.00E-4	> 1.00E-4
ACHN	0.365	1.819	1.777	1.821	1.822	1.747	0.002	97	100	100	95	-99	1.70E-5	3.08E-5	5.57E-5
CAKI-1	0.555	2.369	2.173	2.131	2.138	2.044	0.002	89	87	87	82	-100	1.50E-5	2.83E-5	5.33E-5
RXF 393	0.782	1.487	1.433	1.426	1.430	1.366	0.048	92	91	92	83	-94	1.53E-5	2.94E-5	5.64E-5
SN12C	0.787	2.866	2.748	2.666	2.658	2.569	0.029	94	90	90	86	-96	1.57E-5	2.96E-5	5.57E-5
TK-10	1.311	2.720	2.663	2.614	2.626	2.646	0.078	96	92	93	95	-94	1.73E-5	3.17E-5	5.84E-5
UO-31	0.811	2.988	2.847	2.852	2.782	2.675	-0.006	94	94	91	86	-100	1.56E-5	2.89E-5	5.38E-5
**Prostate Cancer**															
PC-3	0.529	2.518	2.409	2.393	2.373	2.297	0.135	95	94	93	89	-75	1.73E-5	3.50E-5	7.07E-5
DU-145	0.287	1.352	1.412	1.414	1.383	1.313	-0.001	106	106	103	96	-100	1.72E-5	3.09E-5	5.56E-5
**Breast Cancer**															
MCF7	0.351	2.122	1.971	1.963	1.999	1.793	0.144	91	91	93	81	-59	1.67E-5	3.80E-5	8.63E-5
MDA-MB-231/ATCC	0.714	2.141	2.108	2.079	2.127	2.036	0.100	98	96	99	93	-86	1.73E-5	3.30E-5	6.29E-5
HS 578T	1.127	2.454	2.331	2.308	2.264	2.144	0.708	91	89	86	77	-37	1.71E-5	4.71E-5	> 1.00E-4
BT-549	1.020	1.833	1.786	1.744	1.817	1.638	0.105	94	89	98	76	-90	1.43E-5	2.87E-5	5.76E-5
T-47D	0.564	1.494	1.472	1.503	1.396	1.254	0.242	98	101	89	74	-57	1.53E-5	3.67E-5	8.82E-5
MDA-MB-468	0.952	1.914	1.729	1.588	1.627	1.528	0.083	81	66	70	60	-91	1.16E-5	2.49E-5	5.33E-5

Figure S59. 5-Dose assays of compound **26** (NSC 839957).

National Cancer Institute Developmental Therapeutics Program In-Vitro Testing Results			
NSC : D - 839957 / 1	Experiment ID : 2302NS97	Test Type : 08	Units : Molar
Report Date : April 26, 2023	Test Date : February 13, 2023	QNS :	MC :
COMI : 14JPG	Stain Reagent : SRB Dual-Pass Related	SSPL : 0J6G	

Log10 Concentration

Panel/Cell Line	Time Zero	Ctrl	Mean Optical Densities -8.0	-7.0	-6.0	-5.0	-4.0	Percent Growth -8.0	-7.0	-6.0	-5.0	-4.0	GI50	TGI	LC50
**Leukemia**															
CCRF-CEM	0.464	2.168	2.232	2.262	2.347	1.206	0.712	104	106	110	44	15	8.01E-6	> 1.00E-4	> 1.00E-4
HL-60(TB)	0.609	1.865	1.779	2.013	1.897	1.449	0.456	93	112	103	67	-25	1.52E-5	5.32E-5	> 1.00E-4
K-562	0.229	1.895	2.104	2.126	1.993	0.952	0.337	112	114	106	43	6	7.83E-6	> 1.00E-4	> 1.00E-4
MOLT-4	0.612	1.976	2.126	2.232	2.169	0.735	0.610	111	119	114	9	0	4.07E-6	9.05E-5	> 1.00E-4
RPMI-8226	0.702	2.776	2.863	2.796	2.846	1.961	0.733	104	101	103	61	1	1.52E-5	> 1.00E-4	> 1.00E-4
SR	0.235	0.852	0.763	0.822	0.765	0.252	0.297	86	95	86	3	10	2.70E-6	> 1.00E-4	> 1.00E-4
**Non-Small Cell Lung Cancer**															
A549/ATCC	0.534	2.847	2.745	2.753	2.773	2.669	1.713	96	96	97	92	51	> 1.00E-4	> 1.00E-4	> 1.00E-4
EKVX	0.708	2.583	2.373	2.424	2.400	2.246	0.244	89	92	90	82	-66	1.65E-5	3.60E-5	7.85E-5
HOP-62	0.461	1.954	1.819	1.974	1.778	1.570	0.064	91	101	88	74	-86	1.42E-5	2.90E-5	5.95E-5
HOP-92	1.259	1.943	1.953	1.964	1.912	1.788	0.380	102	103	96	77	-70	1.53E-5	3.36E-5	7.33E-5
NCI-H226	0.796	2.276	2.067	2.141	1.935	1.728	0.102	86	91	77	63	-87	1.22E-5	2.63E-5	5.65E-5
NCI-H23	0.842	2.926	2.824	2.848	2.704	2.516	0.115	95	96	89	80	-86	1.52E-5	3.03E-5	6.05E-5
NCI-H322M	0.815	2.445	2.380	2.397	2.268	2.092	-0.002	96	97	89	78	-100	1.44E-5	2.75E-5	5.24E-5
NCI-H460	0.244	2.412	2.421	2.397	2.266	2.170	0.017	100	99	93	89	-93	1.63E-5	3.08E-5	5.79E-5
NCI-H522	1.418	3.140	3.010	3.039	2.987	2.827	0.069	92	94	91	82	-95	1.51E-5	2.90E-5	5.56E-5
**Colon Cancer**															
COLO 205	0.221	1.150	1.160	1.063	0.970	0.809	0.101	101	91	81	63	-55	1.30E-5	3.44E-5	9.15E-5
HCC-2998	0.513	2.440	2.313	2.291	2.382	1.879	0.118	93	92	97	71	-77	1.38E-5	3.01E-5	6.56E-5
HCT-116	0.308	2.481	2.476	2.551	2.490	1.154	0.052	100	103	100	39	-83	6.61E-6	2.08E-5	5.34E-5
HCT-15	0.287	2.535	2.436	2.522	2.422	1.350	0.174	96	99	95	47	-39	8.77E-6	3.51E-5	> 1.00E-4
HT29	0.384	2.511	2.400	2.550	2.477	2.191	0.106	95	102	98	85	-73	1.67E-5	3.46E-5	7.19E-5
KM12	0.466	2.575	2.581	2.533	2.504	2.251	0.020	100	98	97	85	-96	1.56E-5	2.95E-5	5.58E-5
SW-620	0.280	2.320	2.286	2.295	2.097	1.496	0.037	98	99	89	60	-87	1.16E-5	2.55E-5	5.61E-5
**CNS Cancer**															
SF-268	0.825	2.441	2.314	2.329	2.254	2.051	0.034	92	93	88	76	-96	1.41E-5	2.77E-5	5.41E-5
SF-295	0.937	2.955	2.860	3.040	2.894	2.844	0.693	95	104	97	94	-26	2.34E-5	6.08E-5	> 1.00E-4
SF-539	0.757	2.433	2.453	2.407	2.399	2.220	0.022	101	98	98	87	-97	1.59E-5	2.97E-5	5.55E-5
SNB-19	0.542	2.064	2.027	2.034	1.969	1.873	0.111	98	98	94	87	-80	1.68E-5	3.34E-5	6.65E-5
SNB-75	1.800	3.214	3.052	3.042	3.013	2.908	0.013	89	88	86	78	-99	1.44E-5	2.76E-5	5.28E-5
U251	0.474	2.502	2.382	2.395	2.342	2.266	0.069	94	95	92	88	-85	1.66E-5	3.22E-5	6.25E-5
**Melanoma**															
LOX IMVI	0.215	1.942	1.849	1.889	1.840	0.375	0.146	95	97	94	9	-32	3.31E-6	1.67E-5	> 1.00E-4
MALME-3M	0.757	1.714	1.656	1.657	1.582	1.468	0.034	94	94	86	74	-96	1.39E-5	2.74E-5	5.39E-5
M14	0.515	1.888	1.761	1.813	1.754	1.613	0.120	91	94	90	80	-77	1.55E-5	3.24E-5	6.75E-5
MDA-MB-435	0.488	2.347	2.254	2.322	2.210	2.026	0.018	95	99	93	83	-96	1.52E-5	2.90E-5	5.51E-5
SK-MEL-2	1.941	3.023	2.945	2.942	2.898	2.820	0.152	93	93	88	81	-92	1.51E-5	2.94E-5	5.71E-5
SK-MEL-28	0.604	1.962	2.004	2.059	1.983	1.765	0.007	103	107	102	86	-99	1.56E-5	2.91E-5	5.43E-5
SK-MEL-5	0.778	2.954	2.732	2.622	2.720	2.341	-0.004	90	85	89	72	-100	1.34E-5	2.62E-5	5.12E-5
UACC-257	1.447	3.020	2.943	2.950	2.977	2.892	0.129	95	96	97	92	-91	1.69E-5	3.18E-5	5.96E-5
UACC-62	0.869	2.792	2.630	2.685	2.567	2.359	0.030	92	94	88	78	-97	1.44E-5	2.79E-5	5.40E-5
**Ovarian Cancer**															
IGROV1	0.788	2.683	2.751	2.750	2.635	2.331	0.038	104	104	97	81	-95	1.51E-5	2.89E-5	5.55E-5
OVCAR-3	0.480	1.690	1.659	1.691	1.667	1.079	-0.003	97	100	98	50	-100	9.78E-6	2.14E-5	4.63E-5
OVCAR-4	0.865	2.332	2.224	2.291	2.169	1.970	0.004	93	97	89	75	-100	1.40E-5	2.70E-5	5.21E-5
OVCAR-5	0.539	1.579	1.495	1.465	1.381	1.416	0.052	92	89	81	84	-90	1.57E-5	3.04E-5	5.87E-5
OVCAR-8	0.529	2.793	2.739	2.749	2.698	2.362	0.126	98	98	96	81	-76	1.57E-5	3.28E-5	6.81E-5
NCI/ADR-RES	0.489	1.989	1.919	2.040	1.780	1.657	0.215	95	103	86	78	-56	1.61E-5	3.81E-5	9.00E-5
SK-OV-3	0.717	1.959	1.897	1.982	1.917	1.719	0.079	95	102	97	81	-89	1.52E-5	2.99E-5	5.89E-5
**Renal Cancer**															
786-0	0.787	2.557	2.434	2.492	2.552	2.470	0.066	93	96	100	95	-92	1.74E-5	3.23E-5	5.98E-5
A498	1.177	2.110	1.988	2.068	2.056	2.018	2.000	87	95	94	90	88	> 1.00E-4	> 1.00E-4	> 1.00E-4
ACHN	0.365	1.819	1.841	1.923	1.778	1.705	-0.004	101	107	97	92	-100	1.66E-5	3.02E-5	5.49E-5
CAKI-1	0.555	2.369	2.164	2.200	2.095	1.894	-0.007	89	91	85	74	-100	1.37E-5	2.66E-5	5.16E-5
RXF 393	0.782	1.487	1.522	1.490	1.433	1.225	0.050	105	100	92	63	-94	1.21E-5	2.52E-5	5.26E-5
SN12C	0.787	2.866	2.712	2.789	2.666	2.348	.	93	96	90	75	-100	1.39E-5	2.68E-5	5.18E-5
TK-10	1.311	2.720	2.664	2.655	2.742	2.769	0.078	96	95	102	103	-94	1.87E-5	3.34E-5	5.98E-5
UO-31	0.811	2.988	2.878	2.849	2.675	2.246	-0.011	95	94	86	66	-100	1.25E-5	2.50E-5	5.00E-5
**Prostate Cancer**															
PC-3	0.529	2.518	2.390	2.536	2.344	2.010	0.234	94	101	91	74	-56	1.54E-5	3.73E-5	9.02E-5
DU-145	0.287	1.352	1.336	1.345	1.321	1.112	-0.009	98	99	97	77	-100	1.43E-5	2.73E-5	5.23E-5
**Breast Cancer**															
MCF7	0.351	2.122	2.006	2.059	1.961	0.567	0.119	93	96	91	12	-66	3.31E-6	1.43E-5	6.23E-5
MDA-MB-231/ATCC	0.714	2.141	2.068	2.149	2.029	1.780	0.005	95	101	92	75	-99	1.39E-5	2.69E-5	5.20E-5
HS 578T	1.127	2.454	2.228	2.317	2.214	1.904	0.782	83	90	82	59	-31	1.25E-5	4.53E-5	> 1.00E-4
BT-549	1.020	1.833	1.763	1.804	1.787	1.459	0.051	91	96	94	54	-95	1.06E-5	2.30E-5	4.99E-5
T-47D	0.564	1.494	1.391	1.384	1.474	0.792	0.100	89	88	98	24	-82	4.48E-6	1.69E-5	4.98E-5
MDA-MB-468	0.952	1.914	1.923	1.887	1.632	1.387	0.114	101	97	71	45	-88	6.46E-6	2.18E-5	5.18E-5

Figure S60. Mean graph of compound **24** (NSC 838520).

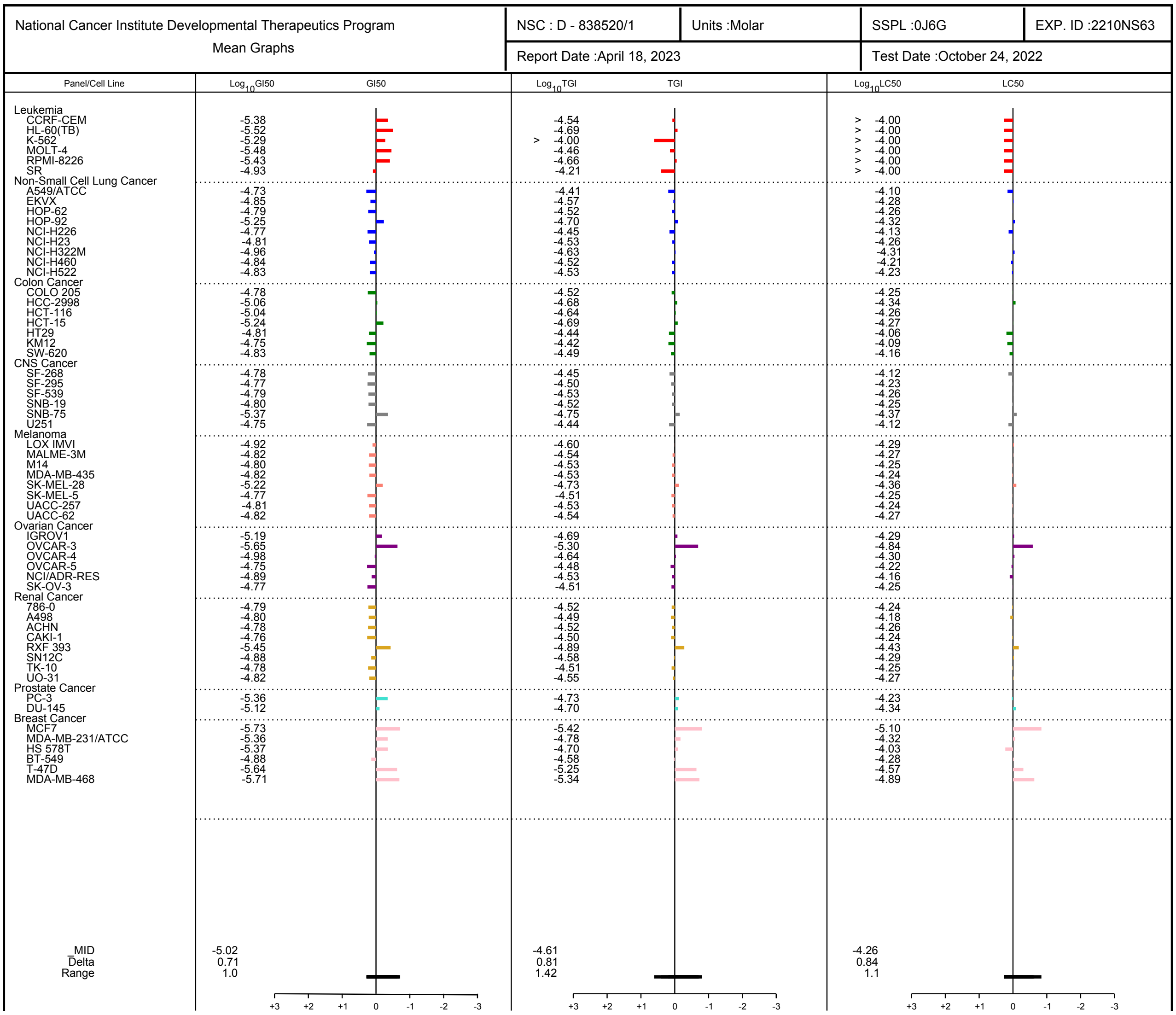

Figure S61. Mean graph of compound **25** (NSC 839956).

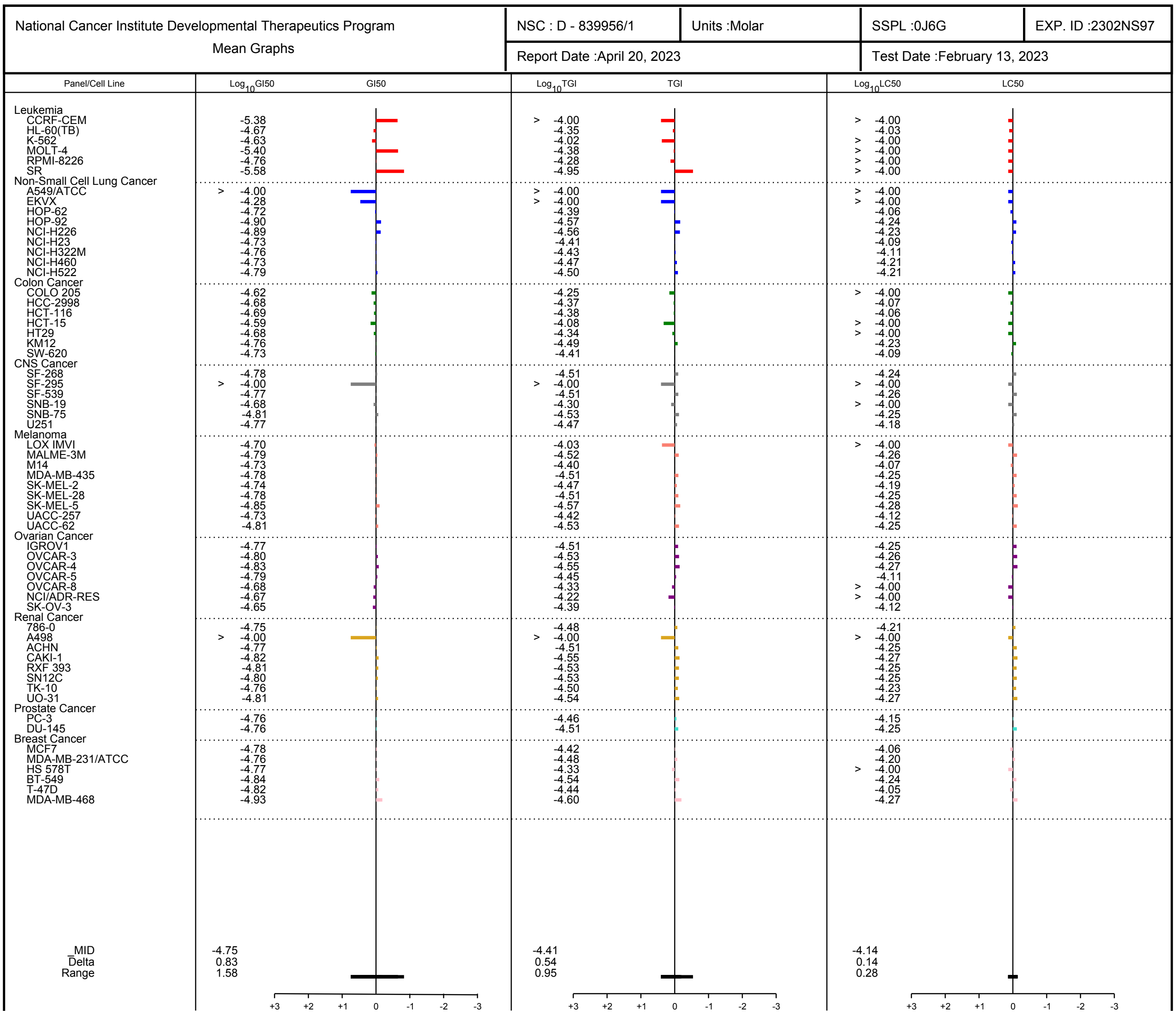

Figure S62. Mean graph of compound **26** (NSC 839957).

National Cancer Institute Developmental Therapeutics Program Mean Graphs	NSC : D - 839957/1	Units :Molar	SSPL :0J6G	EXP. ID :2302NS97
	Report Date :April 18, 2023		Test Date :February 13, 2023	

Panel/Cell Line	Log_{10} GI50	Log_{10} TGI	Log_{10} LC50
**Leukemia**			
CCRF-CEM	-5.10	> -4.00	> -4.00
HL-60(TB)	-4.82	-4.27	> -4.00
K-562	-5.11	> -4.00	> -4.00
MOLT-4	-5.39	-4.04	> -4.00
RPMI-8226	-4.82	> -4.00	> -4.00
SR	-5.57	> -4.00	> -4.00
**Non-Small Cell Lung Cancer**			
A549/ATCC	> -4.00	> -4.00	> -4.00
EKVX	-4.78	-4.44	-4.11
HOP-62	-4.85	-4.54	-4.23
HOP-92	-4.81	-4.47	-4.13
NCI-H226	-4.91	-4.58	-4.25
NCI-H23	-4.82	-4.52	-4.22
NCI-H322M	-4.84	-4.56	-4.28
NCI-H460	-4.79	-4.51	-4.24
NCI-H522	-4.82	-4.54	-4.26
**Colon Cancer**			
COLO 205	-4.89	-4.46	-4.04
HCC-2998	-4.86	-4.52	-4.18
HCT-116	-5.18	-4.68	-4.27
HCT-15	-5.06	-4.45	> -4.00
HT29	-4.78	-4.46	-4.14
KM12	-4.81	-4.53	-4.25
SW-620	-4.93	-4.59	-4.25
**CNS Cancer**			
SF-268	-4.85	-4.56	-4.27
SF-295	-4.63	-4.22	> -4.00
SF-539	-4.80	-4.53	-4.26
SNB-19	-4.78	-4.48	-4.18
SNB-75	-4.84	-4.56	-4.28
U251	-4.78	-4.49	-4.20
**Melanoma**			
LOX IMVI	-5.48	-4.78	> -4.00
MALME-3M	-4.86	-4.56	-4.27
M14	-4.81	-4.49	-4.17
MDA-MB-435	-4.82	-4.54	-4.26
SK-MEL-2	-4.82	-4.53	-4.24
SK-MEL-28	-4.81	-4.54	-4.26
SK-MEL-5	-4.87	-4.58	-4.29
UACC-257	-4.77	-4.50	-4.22
UACC-62	-4.84	-4.55	-4.27
**Ovarian Cancer**			
IGROV1	-4.82	-4.54	-4.26
OVCAR-3	-5.01	-4.67	-4.33
OVCAR-4	-4.86	-4.57	-4.28
OVCAR-5	-4.80	-4.52	-4.23
OVCAR-8	-4.80	-4.48	-4.17
NCI/ADR-RES	-4.79	-4.42	-4.05
SK-OV-3	-4.82	-4.52	-4.23
**Renal Cancer**			
786-0	-4.76	-4.49	-4.22
A498	> -4.00	> -4.00	> -4.00
ACHN	-4.78	-4.52	-4.26
CAKI-1	-4.86	-4.58	-4.29
RXF 393	-4.92	-4.60	-4.28
SN12C	-4.86	-4.57	-4.29
TK-10	-4.73	-4.48	-4.22
UO-31	-4.90	-4.60	-4.30
**Prostate Cancer**			
PC-3	-4.81	-4.43	-4.04
DU-145	-4.85	-4.56	-4.28
**Breast Cancer**			
MCF7	-5.48	-4.84	-4.21
MDA-MB-231/ATCC	-4.86	-4.57	-4.28
HS 578T	-4.90	-4.34	> -4.00
BT-549	-4.97	-4.64	-4.30
T-47D	-5.35	-4.77	-4.30
MDA-MB-468	-5.19	-4.66	-4.29
_MID	-4.88	-4.47	-4.19
Delta	0.69	0.37	0.14
Range	1.57	0.84	0.33

Bar graph axes (GI50, TGI, LC50): +3 +2 +1 0 -1 -2 -3

Figure S63. Dose response curves of compound **24** (NSC 838520).

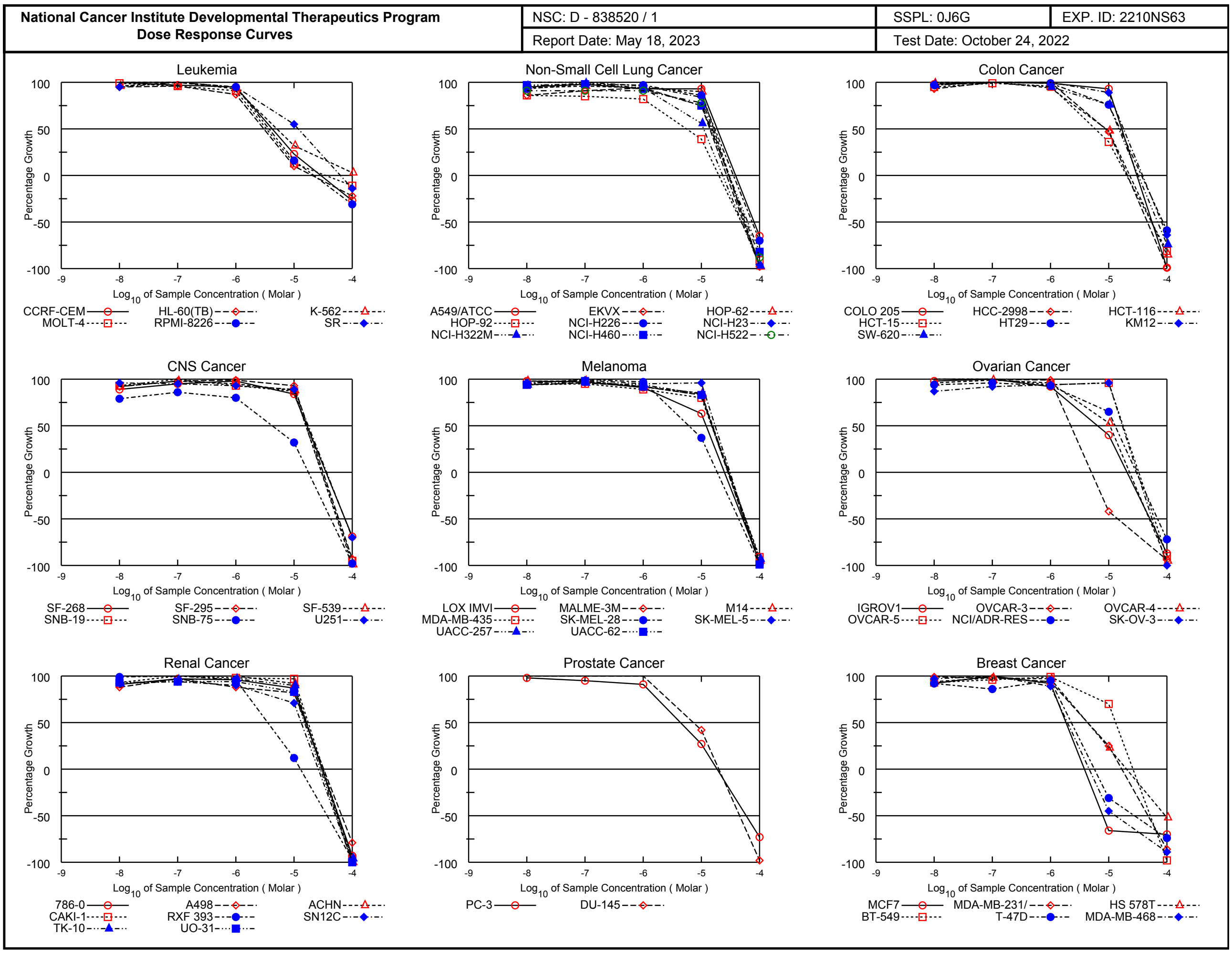

Figure S64. Dose response curves of compound **25** (NSC 839956).

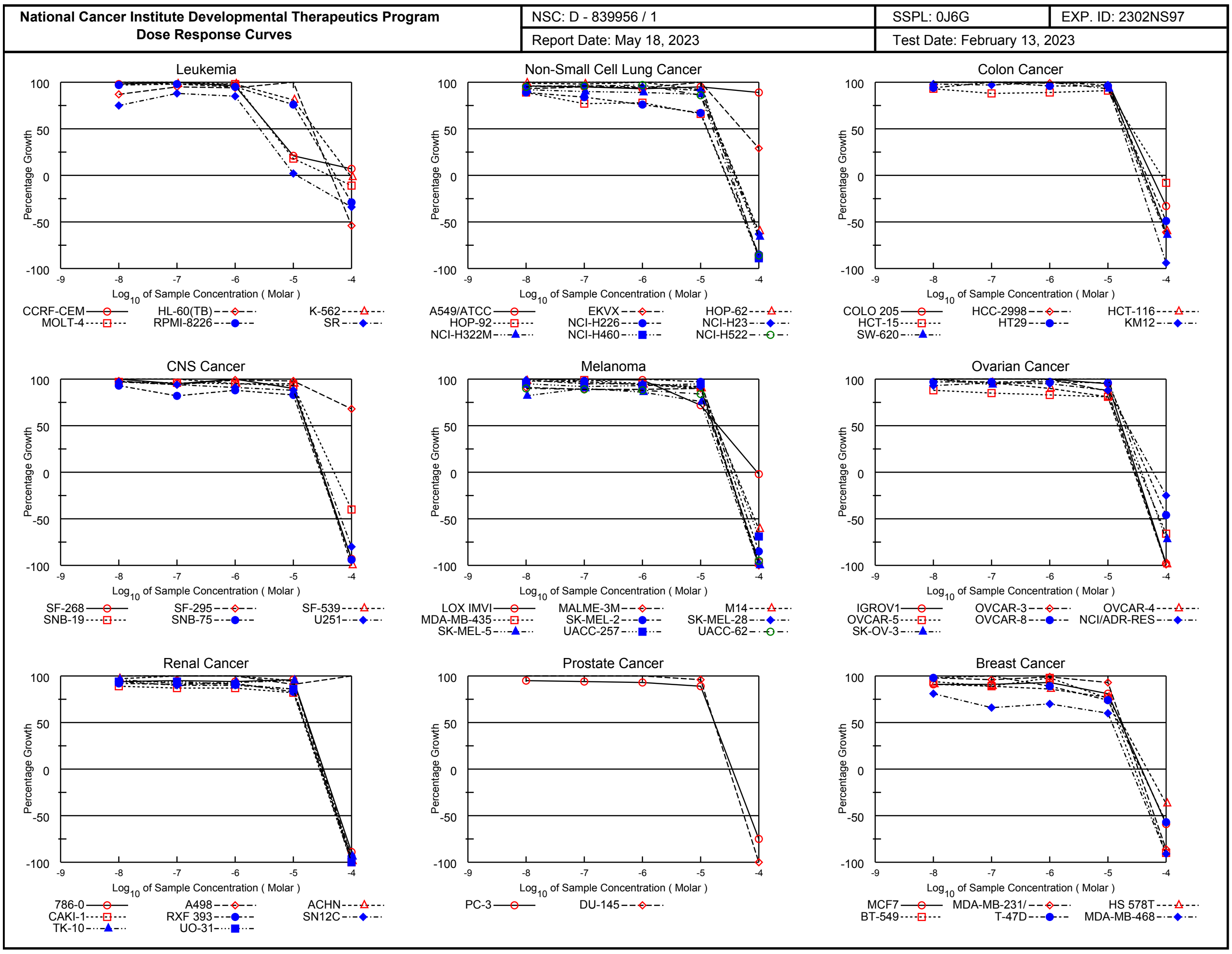

Figure S65. Dose response curves of compound **26** (NSC 839957).

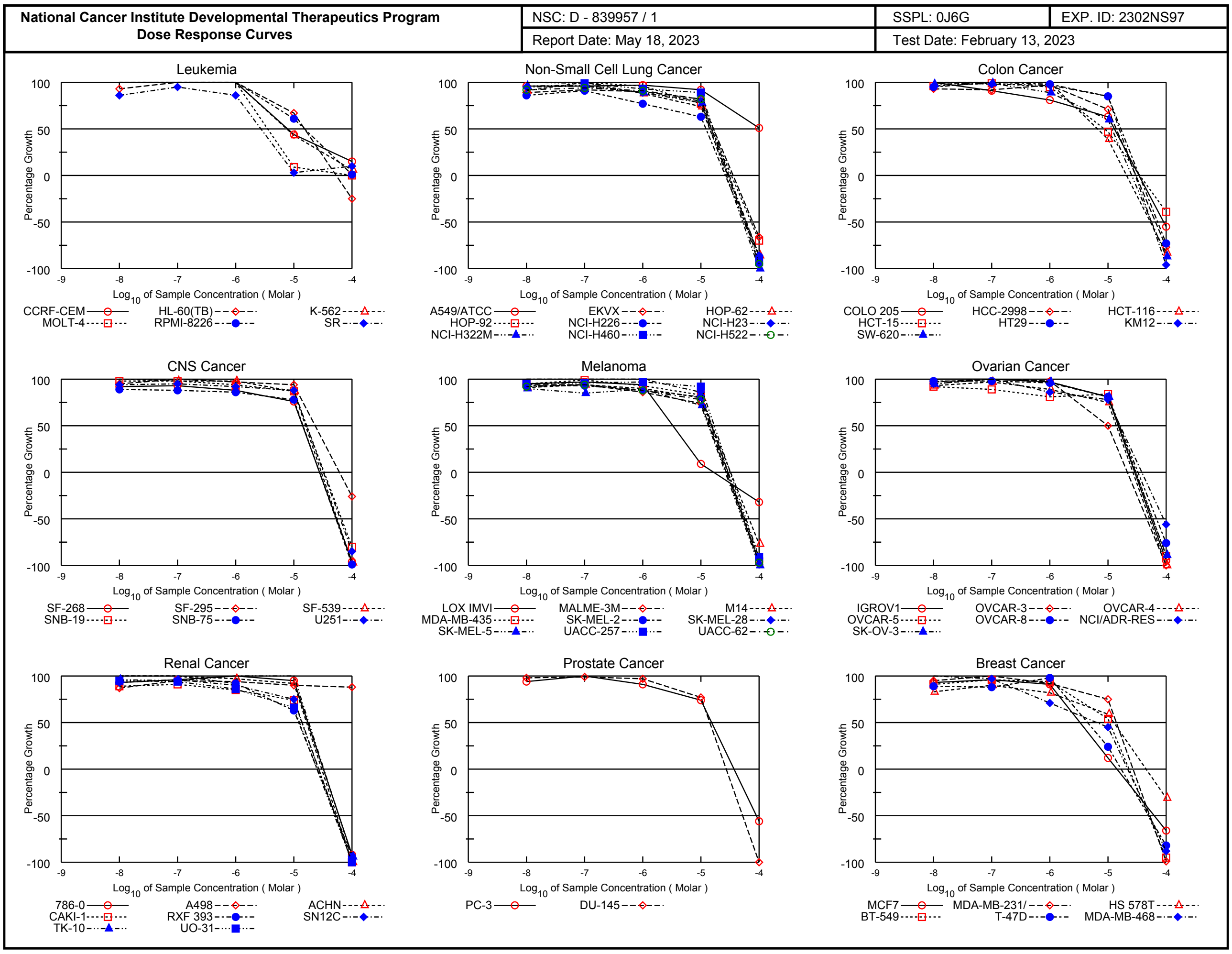

Table S1. Percentage of cytotoxicity of compounds 4, 5, 6, 8, 9, 10, 11, 13, 14, 22, 23, 24, 25 and 26 (10 µM) on NB4 cells viability, assayed with MTS test, after 24 h, 48 h and 72 h at 10 µM concentration.

Compound	% Cytotoxicity		
	**24h**	**48h**	**72h**
**4**	22.0 ± 2.4	70.7 ± 17.7*	61.0 ± 15.7*
**5**	2.3 ± 0.1	35.3 ± 5.7	14.7 ± 1.0
**6**	7.7 ± 0.9	62.3 ± 2.2*	40.0 ± 5.4*
**8**	51.0 ± 0.6*	58.0 ± 20.0*	64.0 ± 13.3*
**9**	8.3 ± 0.8	72.3 ± 18.0*	54.3 ± 5.0*
**10**	22.7 ± 1.7	64.0 ± 13.3*	25.0 ± 3.5
**11**	-[a]	32.7 ± 10.2	13.3 ± 1.4
**13**	7.0 ± 0.7	32.3 ± 3.9	40.0 ± 1.2*
**14**	40.0 ± 2.1*	54.3 ± 19.4*	49.7 ± 1.7*
**22**	-[a]	20.0 ± 3.7	22.3 ± 2.7
**23**	25.0 ± 2.1	62.3 ± 20.1*	26.3 ± 4.8
**24**	55.3 ± 3.4*	75.0 ± 16.5*	59.7 ± 14.2*
**25**	54.7 ± 3.1*	54.3 ± 18.4*	37.3 ± 0.7
**26**	51.7 ± 5.3*	76.7 ± 14.5*	72.7 ± 10.0*

*Data represent mean ± standard error of three independent experiments performed in duplicate. *$P<0.05$ versus control (CTR) cells. Analysis was ANOVA followed by Sidak's multiple comparison test. a no cytotoxicity.*

Table S2. Percentage of cytotoxicity for compounds 4, 8, 9, 24, 25 and 26 on NB4 cells viability, assayed with MTS test, after 72 h at 100 nM and 1 μM concentrations.

Compound	% Cytotoxicity after 72h	
	**100 nM**	**1 μM**
**4**	-[a]	-[a]
**8**	24.3 ± 0.5	24.0 ± 0.4
**9**	-[a]	-[a]
**24**	23.0 ± 0.9	27.0 ± 0.2
**25**	20.0 ± 1.7	21.3 ± 2.7
**26**	9.3 ± 0.7	20.3 ± 0.4

Data represent mean ± standard error of three independent experiments performed in duplicate. Analysis was ANOVA followed by Sidak's multiple comparison test. a no cytotoxicity.

Table S3. Percentage of cytotoxicity of compounds 4, 5, 6, 8, 9, 10, 11, 13, 14, 22, 23, 24, 25 and 26 (10 μM) on JURKAT cells viability, assayed with MTS test, after 24 h, 48 h and 72 h at 10 μM concentration.

**Compound**	**% Cytotoxicity**		
	**24h**	**48h**	**72h**
**4**	5.3 ± 0.1	17.0 ± 1.7	6.3 ± 0.2
**5**	26.0 ± 1.0*	30.3 ± 3.9	17.0 ± 1.7
**6**	20.0 ± 0.7	16.0 ± 0.5	-[a]
**8**	14.3 ± 0.4	3.3 ± 0.1	14.7 ± 0.2
**9**	35.3 ± 0.8*	56.3 ± 1.9*	50.0 ± 1.2*
**10**	41.0 ± 4.0*	53.7 ± 4.7*	38.7 ± 2.9
**11**	48.3 ± 1.4*	65.3 ± 12.2*	56.0 ± 11.8*
**13**	17.0 ± 0.8	-[a]	-[a]
**14**	14.0 ± 0.3	-[a]	-[a]
**22**	30.0 ± 0.5*	23.3 ± 1.8	2.0 ± 0.1
**23**	73.0 ± 4.7*	74.0 ± 17.3*	69.3 ± 2.0*
**24**	67.7 ± 11.2*	42.0 ± 10.7*	32.8 ± 6.2
**25**	68.7 ± 11.8*	66.3 ± 22.8*	39.0 ± 12.2
**26**	65.0 ± 14.7*	50.5 ± 9.8*	38.5 ± 12.5

*Data represent mean ± standard error of three independent experiments performed in duplicate. *P<0.05 versus control (CTR) cells. Analysis was ANOVA followed by Sidak's multiple comparison test. a no cytotoxicity.*

Table S4. Percentage of cytotoxicity of compounds **4**, **5**, **6**, **8**, **9**, **10**, **11**, **13**, **14**, **22,** **23**, **24**, **25** and **26** (10 μM) on SH-SY5Y cells viability, assayed with MTS test, after 24 h, 48 h and 72 h at 10 μM concentration.

**Compound**	**% Cytotoxicity**		
	**24h**	**48h**	**72h**
**4**	8.3 ± 0.4	11.3 ± 0.1	-[a]
**5**	2.3 ± 0.1	5.0 ± 0.1	-[a]
**6**	12.3 ± 0.5	7.3 ± 0.2	-[a]
**8**	10.0 ± 0.1	9.7 ± 0.3	-[a]
**9**	4.0 ± 0.3	27.3 ± 3.1	8.0 ± 1.5
**10**	8.3 ± 0.2	18.0 ± 1.5	24.0 ± 0.7
**11**	14.5 ± 1.3	23.0 ± 4.7	15.7 ± 4.0
**13**	18.3 ± 0.9	13.0 ± 0.5	-[a]
**14**	5.0 ± 0.1	11.7 ± 0.2	-[a]
**22**	16.3 ± 1.0	9.7 ± 0.1	-[a]
**23**	35.8 ± 4.1*	24.5 ± 5.6	43.7 ± 4.0
**24**	44.6 ± 6.7*	33.7 ± 5.2	46.3 ± 15.7
**25**	40.0 ± 5.1*	34.0 ± 6.2	35.0 ± 5.1
**26**	35.3 ± 2.4*	12.0 ± 2.8	-[a]

*Data represent mean ± standard error of three independent experiments performed in duplicate. *P<0.05 versus control (CTR) cells. Analysis was ANOVA followed by Sidak's multiple comparison test. [a] no cytotoxicity.*

Table S5. Percentage of cytotoxicity of compounds **4**, **5**, **6**, **8**, **9**, **10**, **11**, **13**, **14**, **22,** **23**, **24**, **25** and **26** (10 µM) on A375 cells viability, assayed with MTS test, after 24 h, 48 h and 72 h at 10 µM concentration.

**Compound**	**% Cytotoxicity**		
	**24h**	**48h**	**72h**
**4**	16.0 ± 0.7*	15.7 ± 1.3*	12.7 ± 0.5
**5**	6.7 ± 0.1	1.0 ± 0.1	11.3 ± 0.1
**6**	13.0 ± 0.1*	-[a]	7.3 ± 0.1
**8**	33.3 ± 0.4*	26.0 ± 0.2*	-[a]
**9**	18.3 ± 0.3*	6.3 ± 0.1	-[a]
**10**	16.0 ± 0.9*	-[a]	-[a]
**11**	16.3 ± 0.7*	-[a]	-[a]
**13**	16.3 ± 0.1*	9.3 ± 0.8	9.7 ± 0.4
**14**	15.0 ± 0.1*	14.0 ± 0.6	25 ± 2.9*
**22**	9.7 ± 0.1	-[a]	3.7 ± 0.2
**23**	32.0 ± 1.4*	-[a]	-[a]
**24**	60.3 ± 2.2*	73.7 ± 0.9*	56.7 ± 2.1*
**25**	23.3 ± 0.1*	-[a]	-[a]
**26**	28.7 ± 0.1*	-[a]	-[a]

Data represent mean ± standard error of three independent experiments performed in duplicate. *P<0.05 versus control (CTR) cells. Analysis was ANOVA followed by Sidak's multiple comparison test. [a] no cytotoxicity.

Table S6. Percentage of cytotoxicity of compounds **4**, **5**, **6**, **8**, **9**, **10**, **11**, **13**, **14**, **22**, **23**, **24**, **25** and **26** (10 µM) on MAHLAVU cells viability, assayed with MTS test, after 24 h, 48 h and 72 h at 10 µM concentration.

Compound	% Cytotoxicity		
	24h	48h	72h
**4**	-[a]	-[a]	-[a]
**5**	-[a]	-[a]	-[a]
**6**	-[a]	-[a]	-[a]
**8**	-[a]	4.7 ± 0.1	-[a]
**9**	-[a]	16.0 ± 0.1	8.3 ± 0.4
**10**	-[a]	5.3 ± 0.1	-[a]
**11**	-[a]	11.3 ± 0.3	-[a]
**13**	-[a]	-[a]	-[a]
**14**	-[a]	6 ± 0.2	4.0 ± 0.0
**22**	-[a]	-[a]	-[a]
**23**	-[a]	18.3 ± 0.2	17.7 ± 0.6
**24**	16.8 ± 1.5*	39.5 ± 9.4*	32.8 ± 6.1*
**25**	9.7 ± 0.1	20.0 ± 0.5	3.0 ± 0.1
**26**	-[a]	0.7 ± 0.1	-[a]

Data represent mean ± standard error of three independent experiments performed in duplicate. *P<0.05 versus control (CTR) cells. Analysis was ANOVA followed by Sidak's multiple comparison test. [a] no cytotoxicity.

Table S7. Percentage of cytotoxicity of compounds **11**, **24**, **25** and **26** (10 µM) on lymphocytes cells viability, assayed with MTS test, after 24 h of treatment at 10 µM concentration.

Compound	% Cytotoxicity
	24h
**11**	19.3 ± 0.9*
**24**	33.4 ± 2.2*
**25**	26.5 ± 1.7*
**26**	24.8 ± 1.1*

*Data represent mean ± standard error of three independent experiments performed in duplicate. *P<0.05 versus control (CTR) cells. Analysis was ANOVA followed by Sidak's multiple comparison test.* [a] *no cytotoxicity.*
